# Axial spondyloarthritis patients have altered mucosal IgA response to oral and fecal microbiota

**DOI:** 10.3389/fimmu.2022.965634

**Published:** 2022-09-28

**Authors:** Tejpal Gill, Patrick Stauffer, Mark Asquith, Ted Laderas, Tammy M. Martin, Sean Davin, Matthew Schleisman, Claire Ramirez, Kimberly Ogle, Ingrid Lindquist, Justine Nguyen, Stephen R. Planck, Carley Shaut, Sarah Diamond, James T. Rosenbaum, Lisa Karstens

**Affiliations:** ^1^ Division of Arthritis and Rheumatic Diseases, Department of Medicine, Oregon Health & Science University, Portland, OR, United States; ^2^ Casey Eye Institute/Department of Ophthalmology, School of Medicine, Oregon Health & Science University, Portland, OR, United States; ^3^ Division of Bioinformatics and Computational Biomedicine, Department of Medical Informatics and Clinical Epidemiology, Oregon Health & Science University, Portland, OR, United States; ^4^ Department of Molecular Microbiology & Immunology, Oregon Health & Science University, Portland, OR, United States; ^5^ Department of Medicine, Oregon Health & Science University, Portland, OR, United States; ^6^ Laboratory of Immunogenetics, Oregon Health & Science University, Portland, OR, United States; ^7^ Department of Cell Biology, Oregon Health & Science University, Portland, OR, United States; ^8^ Legacy Devers Eye Institute, Portland, OR, United States; ^9^ Division of Urogynecology, Department of Obstetrics and Gynecology Oregon Health & Science University, Portland, OR, United States

**Keywords:** HLA-B27, fecal microbiome, salivary microbiome, predictive metabolomics, axial spondyloarthritis (AxSpA)

## Abstract

Axial spondyloarthritis (axSpA) is an inflammatory arthritis involving the spine and the sacroiliac joint with extra-articular manifestations in the eye, gut, and skin. The intestinal microbiota has been implicated as a central environmental component in the pathogenesis of various types of spondyloarthritis including axSpA. Additionally, alterations in the oral microbiota have been shown in various rheumatological conditions, such as rheumatoid arthritis (RA). Therefore, the aim of this study was to investigate whether axSpA patients have an altered immunoglobulin A (IgA) response in the gut and oral microbial communities. We performed 16S rRNA gene (16S) sequencing on IgA positive (IgA^+^) and IgA negative (IgA^-^) fractions (IgA-SEQ) from feces (n=17 axSpA; n=14 healthy) and saliva (n=14 axSpA; n=12 healthy), as well as on IgA-unsorted fecal and salivary samples. PICRUSt2 was used to predict microbial metabolic potential in axSpA patients and healthy controls (HCs). IgA-SEQ analyses revealed enrichment of several microbes in the fecal (*Akkermansia*, *Ruminococcaceae*, *Lachnospira*) and salivary (*Prevotellaceae*, *Actinobacillus*) microbiome in axSpA patients as compared with HCs. Fecal microbiome from axSpA patients showed a tendency towards increased alpha diversity in IgA^+^ fraction and decreased diversity in IgA^-^ fraction in comparison with HCs, while the salivary microbiome exhibits a significant decrease in alpha diversity in both IgA^+^ and IgA^-^ fractions. Increased IgA coating of *Clostridiales Family XIII* in feces correlated with disease severity. Inferred metagenomic analysis suggests perturbation of metabolites and metabolic pathways for inflammation (oxidative stress, amino acid degradation) and metabolism (propanoate and butanoate) in axSpA patients. Analyses of fecal and salivary microbes from axSpA patients reveal distinct populations of immunoreactive microbes compared to HCs using the IgA-SEQ approach. These bacteria were not identified by comparing their relative abundance alone. Predictive metagenomic analysis revealed perturbation of metabolites/metabolic pathways in axSpA patients. Future studies on these immunoreactive microbes may lead to better understanding of the functional role of IgA in maintaining microbial structure and human health.

## Introduction

Axial spondyloarthritis (axSpA) is an immune-mediated inflammatory arthritis, which affects the sacroiliac and spinal joints, and is associated with microscopic lesions or inflammation in the gut (1). It includes ankylosing spondylitis (AS), in which imaging evidence of inflammation in spine or sacroiliac joints is observed, and non-radiographic axSpA in which radiographic changes are not observed. While the genetic association of AS with the major histocompatibility molecule HLA-B27 has been known for almost five decades (2), the mechanistic link remains elusive. Like several other complex polygenic diseases such as inflammatory bowel disease (IBD), diabetes mellitus and multiple sclerosis, gene-environment interactions and particularly host-microbiota interactions have been centrally implicated in pathogenesis (reviewed in (3)). The latter is well supported by the observations that patients with AS have a dysbiotic gut microbiota and over half exhibit subclinical bowel inflammation (4, 5). Furthermore, a recent study found that gut microbial dysbiosis is associated with worst disease activity in patients with axSpA (6).

Previously, we have shown that HLA-B27 expression perturbs the gut microbiota in an experimental model of spondyloarthritis (SpA) (7–9). In this model, transgenic rats expressing multiple copies of human HLA-B27 in conjunction with human β2-microglobulin (HLA-B27 TG) develop spontaneous bowel and joint disease (10). Enhanced mucosal immune responses to the gut microbiota strongly correlate with disease severity in these animals. For instance, HLA-B27 TG rats with arthritis elicit a stronger microbiota-specific IgA response (increased frequency of IgA-coated microbes) than control HLA-B7 TG animals without joint disease (8). Recently, we have shown that healthy individuals carrying the HLA-B27 allele also have altered gut microbial composition (11). While multiple studies employing 16S rRNA (16S) and metagenomic sequencing have revealed gut microbial dysbiosis associated with various spondyloarthropathies (4, 5, 12–15), identification of a causal microbe has been elusive. Compositional analysis of the microbial community may not be sufficient to determine the interactions between microbes and the host immune response. To identify such bacteria that preferentially affect disease susceptibility and severity, we employed IgA-sequencing (IgA-SEQ), which couples flow cytometry sorting of IgA-coated bacteria and 16S sequencing, to identify IgA coated microbes (16). This technique has been used to determine pathologically relevant IgA coated microbes in Crohn’s disease (CD)-associated SpA (17). Colonization of germ-free mice with IgA coated microbes, particularly with adherent-invasive *E. coli* (AIEC) pathotype, induced Th17 immunity through microbial metabolic enzyme propanediol dehydratase (17), which induced IL-1β production by mononuclear phagocytes, thus perpetuating inflammation (18). These findings identified immune cells as metabolic sensors and highlight the importance of IgA coated microbes and their metabolites as potential therapeutic targets in CD treatment. AS and CD share several aspects of clinical overlap including bowel, eye, and joint disease (19). However, this type of investigation of the mucosal immune response and metabolic function of IgA coated microbes and has not yet been reported in axSpA patients.

The gut is the largest immune organ, facilitating interaction between the gut microbiota and local mucosal immune response. We hypothesize that other mucosal sites that are home to microbes contribute significantly to systemic rheumatic disease, particularly the oral cavity. It has been shown that patients with rheumatoid arthritis (RA) have a higher prevalence of severe periodontitis as compared to healthy controls (20). Disease activity in RA also tracks with periodontal disease activity with a corresponding partial resolution of microbial dysbiosis upon treatment (21, 22). A recent study reported increased incidence of periodontitis in axSpA patients in comparison with the healthy controls; however, they did not observe differences between the bacterial richness and diversity or community structure (23). Furthermore, it has not been elucidated if perturbation of the oral cavity mucosal barrier is also altered in those with axSpA. Our hypothesis thus extends beyond the gut and investigates if immune modulation of the mucosal barrier occurs in the oral cavity of patients with axSpA.

In this study, we evaluated IgA coating (IgA-SEQ) and traditional 16S sequencing of microbes in the saliva and feces from axSpA patients and healthy controls (HCs). IgA-SEQ analyses identified potentially immune-targeted microbes that were not observed to be differentially abundant using traditional 16S sequencing alone. In addition, differential IgA coating on some fecal and salivary microbes was associated with disease activity. Using a computational approach to predict the microbial function, we found that the IgA^+^ fraction in the fecal and salivary samples showed perturbation of many predictive metabolites and metabolic pathways in axSpA patients. These potentially immune reactive microbes may reveal novel host-microbial interactions in health and disease. Taken together, this study adds to our understanding of HLA-B27-associated host immune response and its interaction with the gut microbes in axSpA.

## Patients and methods

### Study design and participants

We performed a prospective cohort study at the Oregon Health and Science University (OHSU). Subjects were excluded from all cohorts if they were younger than age 18 years, pregnant, had a history of prior intestinal surgery or colon cancer, or had antibiotic use 6 months prior to sample collection. This study was approved by the Institutional Review Board at OHSU and written informed consent was obtained from all participants.

*AxSpA cohort*: Subjects with a diagnosis of axSpA were recruited from either the OHSU rheumatology clinic or *via* the Spondylitis Association of America (SAA). The SAA is a patient advocacy group with physicians on its board, and members with AS are highly engaged and informed. **Supplementary Table 1** lists the detailed location and recruitment status of axSpA patients and HCs (local vs SAA) for the stool and saliva cohort. Medical records were reviewed by a rheumatologist (JTR) to confirm a diagnosis of AS/axSpA based on the modified NY (New York) Criteria and the ASAS (Assessment of Spondyloarthritis International Society) classification respectively (24, 25). A total of 26 subjects with AS/axSpA were enrolled (12 subjects provided fecal samples, 9 provided salivary samples and 5 subjects provided both fecal and salivary samples). Of these, 22 patients had a diagnosis of AS in their rheumatological medical records (11 of those were confirmed with imaging review), while 4 had limited external medical records but were sufficient to verify axSpA, thus our patient cohort is classified as axSpA in this study even though AS is highly represented. All of the patient medical information was reviewed again to confirm axSpA diagnosis during study design. While none of the axSpA subjects had psoriasis or peripheral arthritis, one axSpA patient in the salivary cohort was also diagnosed with IBD. The Bath ankylosing spondylitis disease activity index (BASDAI) was administered to subjects with axSpA. Their current medications, including biologics, non-steroidal anti-inflammatory drugs (NSAIDs) as well as clinical HLA-B27 testing, if known, was also documented.

*Samples*: All subjects enrolled at OHSU provided a blood sample for genomic DNA extraction and subsequent HLA-B typing. Subjects recruited through the SAA shipped fecal and/or salivary samples to our lab using next-day shipping packets. For microbiome analysis, fecal and salivary samples were snap frozen upon collection and stored at -80°C until further analyses. All processing thereafter was performed blinded to patient phenotype. **Supplementary Figure 1** illustrates the study experimental overview.

*HLA-B typing*: Genomic DNA was extracted from anti-coagulated blood by the salting out method performed in a shared core resource at the Casey Eye Institute, OHSU. The LABType XR HLA-B SSO typing kit from One Lambda Thermo Fisher (RSSOX1B) was used according to manufacturer’s instructions in the OHSU Laboratory of Immunogenetics and Transplantation.

### Microbial community analysis

To determine the microbial community structure, genomic DNA from the salivary and fecal samples was isolated using the methods described previously (9). Briefly, genomic DNA was isolated using the Power Soil DNA Isolation Kit (MoBio Laboratories Inc., Carlsbad, CA) according to manufacturer’s instructions. Amplification of the 16S ribosomal RNA genes was performed using the 515–806 primers specified by the Earth Microbiome Project (https://www.earthmicrobiome.org/). Sequencing of the V4 region was accomplished with Illumina MiSeq using barcoded primers (26). For IgA-SEQ, fecal samples were homogenized in phosphate buffered saline (PBS), supernatant was collected and washed in PBS with 1% fetal bovine serum (FBS) and incubated with blocking buffer (PBS with 20% rat serum) for 20 min. The IgA^+^ fraction was stained with phycoerythrin (PE) labeled mouse anti-human IgA (Miltenyi 130-093-128), and enriched using anti-PE magnetic activated cell sorting (MACS) beads, and the negative fraction was collected for IgA^-^ microbes. IgA^+^ fraction was further purified through a FACS Aria (BD Biosciences). Genomic DNA from IgA^+^ and IgA^-^ microbial fractions was isolated and the V4 region of the16S rRNA gene was sequenced as described above.

Sequencing data were demultiplexed and processed using the DADA2 package in R (27). This included quality filtering, denoising using the DADA2 algorithm, chimera detection and removal, and tabulation into amplicon sequence variants (ASVs). Taxonomy assignment was performed using the RDP classifier (for Phylum to Genus annotations) and exact matching (for Species level annotations) with the Silva database (v. 132). Phylogenetic trees were generated using phangorn and decipher. Further analyses were performed using microbiome data analysis packages in R (phyloseq, vegan, MicrobiotaProcess) (28–30). IgA-SEQ data were subjected to decontam (31), a software tool to identify and remove contaminating sequence features in 16S sequencing data, and analyses were performed using in-house written scripts (32), available on Github (https://github.com/KarstensLab/igaseq). Samples that had 7,500 reads per sample in both the IgA^-^ and IgA^+^ fractions were used. The IgA index was calculated as the log ratio of the difference between IgA coated and IgA uncoated bacteria over the sum of IgA coated and IgA uncoated bacteria (IgA index= - (log(IgA^+^ taxon)- log(IgA^-^ taxon))/(log(IgA^+^ taxon) + log(IgA^-^ taxon))) (33). Alpha diversity was evaluated by using multiple indices to determine richness and evenness in the community. While the number of observed genera/ASVs evaluates richness only using counts, the Shannon Index contains a logarithmic values including both richness and evenness, and Inverse Simpson Index focuses mostly on evenness using sum of squared proportions (34). Beta diversity was evaluated using the phylogenetic tree-based metric, UniFrac metric (35) and visualized with Principal Coordinate Analysis (PCoA).

### Predictive metagenomic analysis

To infer the metagenome of oral and gut microbiota from 16S rRNA amplicon sequences obtained, we employed PICRUSt2 (Phylogenetic Investigation of Communities by Reconstruction of Unobserved States 2) (36). PICRUSt2 estimates the metagenomic contribution of gene families in bacteria and archaea using 16S rRNA amplicon sequencing data, while taking into account the copy number of 16S rRNA gene. Functional potential was estimated by mapping 16S rRNA sequencing data to predicted functional annotation *via* alignments and hidden state prediction models (31), using default parameters. We used the quantitative abundance counts that mapped the Kyoto Encyclopedia of Genes and Genomes (KEGG) ortholog (KO) functional categories and focused our analysis on pathways using the multi-organism database MetaCyc (37) and individual metabolites (KO), which are both default functional annotations of PICRUSt2. Overlap between KO is determined between various groups, and area proportional Euler plots were created using Eulerr package in R (38).

### Statistical analysis

All statistical analyses were performed in R. For 16S sequencing and IgA-SEQ analyses, non-parametric tests (Wilcoxon Rank Sum) were used to assess within and between group differences for the alpha diversity measures. Differences in individual microbial relative abundances at the genus and ASV level were assessed with non-parametric tests (Wilcoxon Rank Sum) and corrected for multiple tests using the Benjamini-Hochberg method (39). Differences in overall microbial community structure were evaluated using Permutational Multivariate Analysis of Variance (PERMANOVA). Correlation between relative abundance and disease activity (BASDAI) was performed using Spearman correlation with adjusted p-values after correcting for multiple comparisons using the Benjamini-Hochberg method (39). LEfSe was used to determine the presence of significant discriminant taxa by pairwise comparison (40). For PICRUSt2 analyses, LEfSe was used to identify candidate biomarker features among both individual metabolites (KO) and pathways (MetaCyc) (40). The LEfSe method performs a series of three tests: (1) at the class level (representing diagnosis axSpA or HC) an ANOVA (Kruskal-Wallis sum-rank test) with an alpha of <0.05 was applied (2) at the subclass level (representing patient sex) a Wilcoxon test with an alpha of <0.05 was applied and (3) a linear discriminant analysis (LDA) is finally applied to features to estimate their effect size and provides a ranking order of features most likely to explain group differences. Comparisons were made between IgA^+^ and IgA^-^ fractions from salivary and fecal microbiome data from axSpA patients and HCs.

## Results

### Clinical characteristics defining axSpA patients

To analyze microbial differences between axSpA subjects and HCs, we performed 16S sequencing on the unsorted microbial community and IgA-SEQ to identify IgA^+^ and IgA^-^ sorted fractions from the fecal and salivary samples. The fecal cohort had 31 subjects (17 axSpA patients and 14 HCs), and the salivary cohort had 26 subjects (14 axSpA patients and 12 HCs). While there were no significant differences in age or gender (*P>* 0.2 and 0.6 respectively) between the axSpA patients and HCs for the fecal samples, the HCs were younger (*P=* 0.018), and with less female representation (*P=* 0.038) than the axSpA patients in the salivary cohort. However, both axSpA patients and HCs had similar BMI in the fecal (*P =* 0.5) and salivary (*P =* 0.4) cohort. AxSpA patients had a mean disease activity (BASDAI) of 3.5 in the fecal cohort, and 2.0 in the salivary cohort. The fecal cohort was comprised of 47% axSpA patients on biologics such as adalimumab, etanercept or secukinumab, while 36% of the salivary axSpA cohort were on biologics (**Table 1**). Furthermore, 47% of the fecal cohort and 14% of the salivary cohort of axSpA patients were on NSAIDs. In comparison, 21% of fecal cohort and 17% of salivary cohort healthy individuals reported NSAIDs use (**Table 1**).

**Table 1:** General characteristics of the subjects providing stool and saliva samples for IgA-Seq analyses.

	axSpA vs. HC analysis						
	Stool cohort^*^			Saliva cohort^*^		
	axSpA(n=17)	HC(n=14)	*P* ^†^	axSpA(n=14)	HC(n=12)	*P*
Age, years^‡^	58 (49, 69)	55 (37, 62)	0.2	57 (52, 64)	36 (30, 56)	0.018
Female	10 (59%)	6 (43%)	0.6	12 (86%)	5 (42%)	0.038
Race			0.032			0.13
Asian	0 (0%)	2 (14%)		0 (0%)	2 (17%)	
Black	1 (6%)	0 (0%)		1 (7%)	0 (0%)	
White	16 (94%)	10 (71%)		12 (86%)	7 (58%)	
>1 race	0 (0%)	2 (14%)		1 (7)	3 (25%)	
Unknown	0	0		0	0	
Hispanic or Latino	2 (12%)	1 (7%)	1	0 (0%)	1 (8%)	0.5
BMI, kg/m^2^	27 (23, 32)	26 (21, 30)	0.5	26 (21, 31)	23 (22, 25)	0.4
BASDAI	3.5 (2.4, 5.5)	0.8 (0.5, 1.3)	<0.001	2.0 (1.5, 2.8)	0.6 (0.1, 0.9)	0.003
Unknown	1	1		0	1	
BASFI	3.2 (1.4, 4.2)	0.1 (0.0, 0.5)	0.002	2.1 (1.1, 3.6)	0.0 (0.0, 0.2)	<0.001
Unknown	1	1		0	1	
HLA-B27^+^	16 (94%)	0 (0%)	<0.001	13 (93%)	0 (0%)	<0.001
Unknown	0	7		0	4	
Biologics^§^			0.003			0.039
Yes	8 (47%)	0 (0%)		5 (36%)	0 (0%)	
No	8 (47%)	14 (100%)		8 (57%)	12 (100%)	
Unknown	1 (6%)	0		1 (7%)	0	
NSAIDs^ψ^			0.14			0.06
Yes	8 (47%)	3 (21%)		2 (14%)	2 (14%)	
No	8 (47%)	11 (79%)		10 (71%)	12 (86%)	
Unknown	1 (6%)	0		2 (14%)	0	
Uveitis^¶^	10 (59%)	NA		9 (64%)	NA	
IBD^ω^	0	NA		1 (7%)	NA	

*Five subjects with axial spondyloarthritis (axSpA) and four subjects in the healthy control (HC) category provided both stool and saliva samples.

^†^P values were calculated using the Wilcoxon rank-sum test for continuous numeric values, the Fisher’s exact test for categorical variables with 2 values, and the chi-square test of independence for categorical variables with 3 or more values.

^‡^All statistics are presented as either Median (IQR) or n (%).

^§^Biologics taken within 6 months of providing samples included adalimumab, etanercept or secukinumab.

ψNon-steroidal anti-inflammatory drugs (NSAIDs) taken within 6 months of providing samples included ibuprofen, aspirin, naproxen.

^¶^Uveitis secondary to axSpA. No subjects were known to have active ocular inflammation at the time of providing samples, except one individual with mild uveitis in the Saliva/axSpA group. NA, not applicable in the HC group by definition.

^ω^IBD secondary to axSpA. Only 1 subject in the salivary cohort were known to have IBD. NA, not applicable in the HC group by definition.

### AxSpA patients have altered microbial composition in saliva

**Figure 1:**
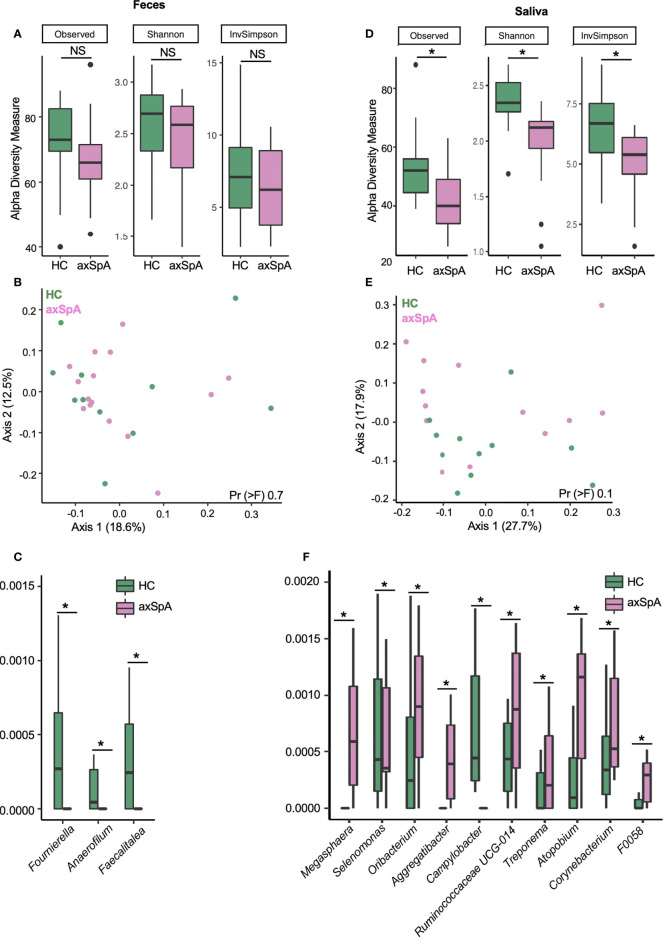
16S sequencing of fecal and salivary samples in axSpA patients and HCs at the genus level. **(A–C)** represent fecal samples and **(D–F)** are salivary samples from axSpA patients (pink) and HC (green). Alpha diversity plots with Observed, Shannon and Inverse Simpson (InvSimpson) indices for **(A)** fecal and **(D)** salivary samples. Microbial composition of **(B)** fecal and **(E)** salivary samples analyzed using the unweighted Unifrac distance represented as a principal coordinate analysis (PCoA) plot. Relative abundance of genus level microbes in **(C)** fecal and **(F)** salivary samples that are different between axSpA patients and HC. *P<0.05; NS not significant.

To characterize microbial diversity in the fecal and salivary samples from patients with axSpA in comparison with the HCs, we performed 16S sequencing on both fecal (**Figures 1A–C**) and salivary (**Figures 1D–F**) samples. We did not observe any differences between the microbial diversity (alpha diversity, **Figure 1A**) and community structure (beta diversity, **Figure 1B**) in the fecal samples from axSpA patients in comparison to HCs. In contrast, salivary samples from axSpA patients showed a decrease in all (Shannon *P* = 0.016, *P_FDR_* = 0.048, observed *P*=0.047, *P_FDR_* = 0.094, and Inverse Simpson *P*= 0.048, *P_FDR_* = 0.094) measures of alpha diversity (**Figure 1D**), however the microbial composition (beta diversity, **Figure 1E**) of axSpA patients was not significantly different than HCs at the genus level. We also compared individual microbial differences between axSpA patients and HCs at the genus level for fecal and salivary samples. We observed many microbes trended towards altered relative abundance in the fecal (e.g., *Anaerofilum*, *Fecalitalea*, *Fournierella; P* < 0.05, *P_FDR <_* 0.79; **Supplementary Table 2**) (**Figure 1C**) as well as in the salivary samples (e.g., *Megasphaera*, *Selenomonas, Oribacterium, Aggregatibacter*, *Campylobacter*, *Ruminococcaceae*_UCG-014, *Treponema*, *Atopobium*, *Corynebacterium* and *F0058*) (**Figure 1F**) of axSpA patients in comparison to HCs (*P* < 0.05, *P_FDR <_* 0.52; **Supplementary Table 2**). These differences no longer reached statistical significance after multiple testing correction. Similar results were seen in both fecal and salivary samples at the species level as measured using ASVs (**Supplementary Figures 2A–F**). Notably, there was a significant difference between the salivary alpha diversity in axSpA patients and HCs (Shannon’s Index, *P=* 0.0006, *P_FDR_* = 0.002), however microbial composition as assessed by beta diversity was not significantly different between axSpA subjects and HCs (*permanova, P* = 0.06) (**Supplementary Figures 2D, E**). We did not observe these differences in the fecal microbial diversity or community structure in axSpA patients in comparison with HCs (**Supplementary Figures 2A, B**). Despite this, relative abundance comparisons of individual microbes at the ASV level showed a trend towards increased abundance (*P*<0.05, *P_FDR_* = 0.74; **Supplementary Table 2**) of *Bacteroides vulgatus*, *Holdemanella biformis* and *Lachnospiraceae* spp. in the fecal microbiome (**Supplementary Figure 2C**). The salivary microbiome showed a trend towards decrease (*P* < 0.05, *P_FDR_ <*0.57; **Supplementary Table 2**). in the abundance of multiple microbes (e.g., *Prevotella histicola, Megasphaera micronuciformis, Prevotella maculosa, Campylobacter concisus, Dialister invisus etc.)* in the axSpA patients as compared with HCs (**Supplementary Figure 2F**).

### Enrichment of IgA-coated microbes in fecal samples of axSpA patients

**Figure 2:**
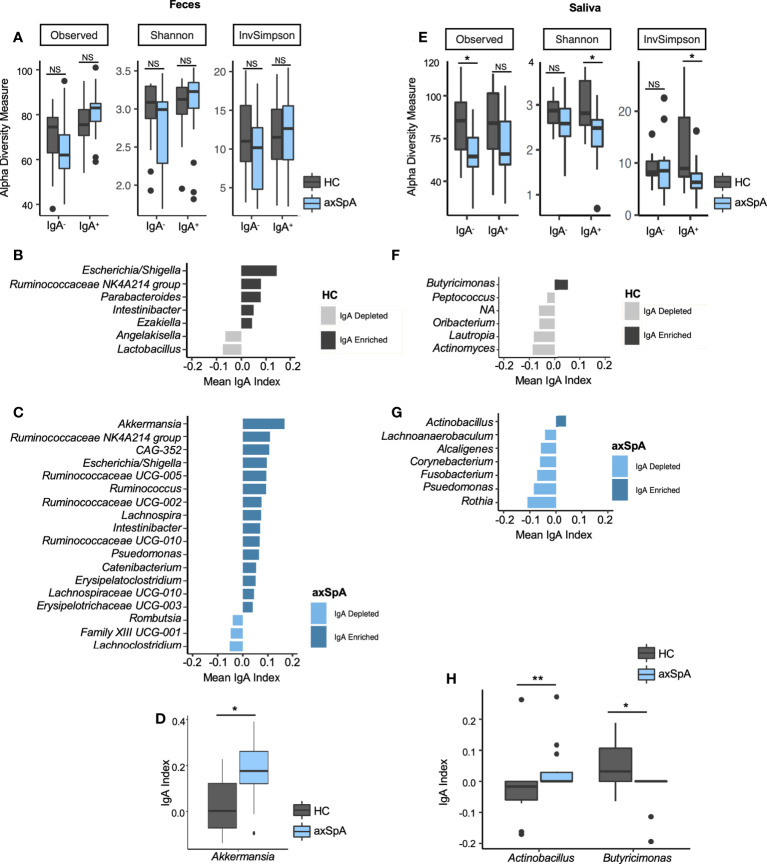
IgA-SEQ analyses of fecal and salivary samples in axSpA patients and healthy controls (HC) at the genus level. **(A–D)** represent fecal samples and **(E–H)** are salivary samples from axSpA patients (blue) and HC (gray). Alpha diversity plots for IgA^-^ and IgA^+^ fractions with observed, Shannon and Inverse Simpson (InvSimpson) indices for **(A)** fecal and **(E)** salivary samples. IgA coating index was calculated for all taxa and significant taxa are shown for fecal samples for HCs **(B)** and patients with AxSpA **(C)**. Similarly, IgA coating index was calculated for all taxa and significant taxa, and significant taxa are shown for salivary samples from HCs **(F)** and axSpA patients **(G)**. IgA index for differentially abundant microbes in fecal **(D)** and salivary **(H)** microbes in axSpA patients and HCs are shown. *P<0.05, **P<0.01; NS not significant.

Although we did not observe differences in the overall microbial composition in fecal samples as measured by 16S sequencing, we suspected that there may be differences in how the host immune system interacted with these microbes. To determine whether microbiota from the fecal and saliva samples can elicit an immune response, we performed IgA-SEQ on IgA^+^ and IgA^-^ microbes sorted from saliva and fecal microbial communities of the axSpA patients and HCs. This analysis revealed a tendency towards increased microbial diversity of IgA^+^ fraction in the fecal microbiome of axSpA patients as compared to the HCs, whereas the IgA^-^ fraction showed a tendency towards loss of diverse in axSpA patients as compared with HCs (**Figure 2A**). On the other hand, salivary microbiome of axSpA patients showed a significant decrease in microbial richness as measured by alpha diversity in both IgA^-^ as well as IgA^+^ fractions as compared to the HCs (**Figure 2E**). The beta diversity comparison (IgA^+^ and IgA^-^ fractions) between axSpA and HCs did not yield any significant differences (data not shown).

Further analyses of IgA-enriched (Increased relative abundance in the IgA^+^ fraction as compared with the IgA^-^ fraction) and depleted (decreased relative abundance of that microbe in IgA^+^ fraction as compared with IgA^-^ fraction) bacteria from patients with axSpA identified a greater number of unique IgA-coated microbial genera in fecal (**Figure 2C**) and salivary (**Figure 2G**) samples. Similarly, there was an increased number of IgA enriched microbes at the ASV level in both fecal and salivary samples from axSpA patients (**Supplementary Figures 3C, G**, respectively). *Akkermansia* has the highest IgA index score among fecal microbes in axSpA patients followed by *Ruminococcaceae* and CAG-352. In addition, axSpA patients had many microbes with IgA enrichment such as *Klebsiella*, *Ruminococcus*, *Lachnospira*, *Pseudomonas* and other members of family *Ruminococcaceae* and *Lachnospiraceae* (**Figure 2C**). Among fecal samples, the phylum Firmicutes represent the majority of microbes coated with IgA in axSpA patients. The IgA^+^ fractions in HCs (**Figure 2B**) also showed some microbes with increased IgA coating in the fecal samples (*Escherichia/Shigella*, *Ruminococcaceae*-NK4A214, *Parabacteroides*). Interestingly, some of the IgA enriched microbes overlapped between axSpA patients and HCs, such as those belonging to genus *Escherichia/Shigella*, *Ruminococcaceae*-NK4A214, *Intestinibacter* (**Figures 2B, C**). Despite significant changes in microbial diversity and community structure in the saliva of axSpA patients in comparison to HCs (**Figures 1B, D**), it had a smaller number of IgA enriched microbes in both HCs and axSpA patients (**Figures 2D, E** respectively) in comparison to the fecal samples in both HCs and axSpA patients (**Figures 2B, C** respectively). The majority of salivary microbes from axSpA patients and HCs were IgA-depleted, with only the genus *Butyricimonas* being enriched in IgA coating in HCs and *Actinobacillus* (**Figures 2F, G**) being enriched in axSpA patients. Comparing the IgA index for individual microbes, *Akkermansia* was significantly enriched in the IgA^+^ fraction in axSpA patients (**Figure 2D**). In the salivary microbiome, IgA coating of *Actinobacillus* was increased while that of *Butyricimonas* was decreased when compared to HCs (**Figure 2H**). These analyses were also performed at the ASV level and similar results were observed (**Supplementary Figure 3**). Some of the IgA enriched fecal microbes such as members of *Lachnospiraceae* and *Escherichia/Shigella* overlapped between axSpA patients and HCs (**Supplementary Figures 3B, C**). However, there was no overlap between the IgA enriched salivary microbes between axSpA patients and HCs (**Supplementary Figures 3F, G**).

### IgA-SEQ reveals immunoreactive microbes correlating with disease activity

**Figure 3:**
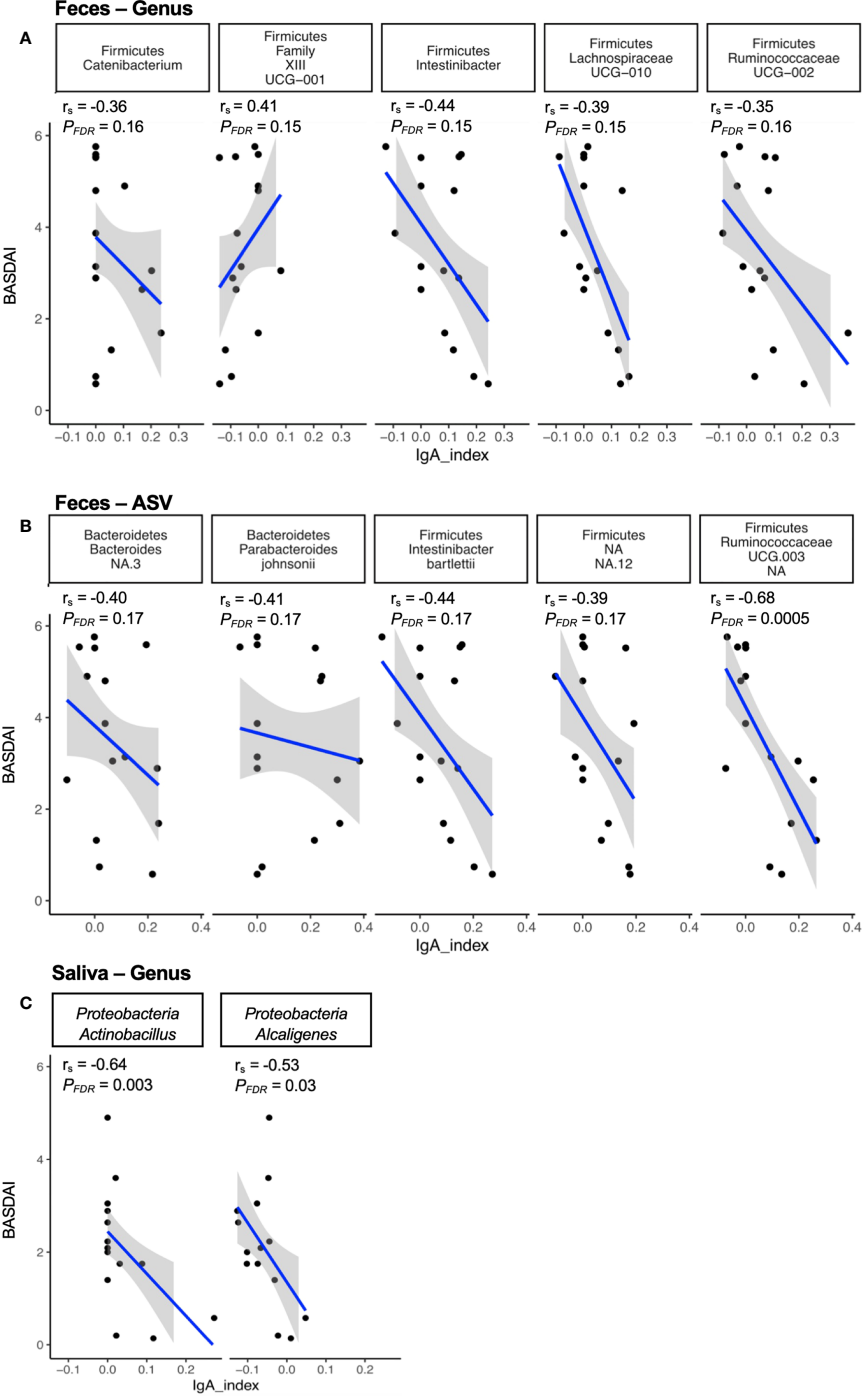
Correlation of immune reactive microbes with disease activity. The IgA index of the immune reactive microbes is correlated with the disease index (BASDAI score) at the genus **(A)** and ASV **(B)** level for fecal microbes, and at the genus **(C)** level for salivary microbes. The strength of each linear association is measured with a correlation coefficient r. The value of r=1 indicates perfect correlation; a positive value denotes a positive correlation and a negative value signifies an inverse correlation. In addition, the adjusted p-value (PFDR) of the correlations after multiple tests correction are also depicted on the plot.

To determine whether IgA enriched microbes in feces from axSpA patients were associated with disease, we correlated genus and ASV level microbes with the disease activity score (measured by BASDAI). The analysis showed a significant negative correlation between *Ruminococcaceae UCG-003* (ASV level, **Figure 3B**) in the fecal community, and a significant negative correlation between genus *Actinobacillus* and *Alcaligenes* in the salivary microbes (**Figure 3C**). In addition, we found many microbes trending towards a correlation between IgA enrichment and disease activity. Of these, genus *Clostridiales Family XIII* correlated positively, while the genera *Catenibacterium*, *Intestinibacter* and members of family *Lachnospiraceae* (UCG010) and *Ruminococcaceae* (UCG-002) trended towards a negative correlation [**Figure 3A**, (*P* < 0.05, *P_FDR_* < 0.20)]. At the ASV level (**Figure 3B**), we observed a trend towards negative correlation with [*Bacteroides* spp., *Parabacteroides johnsonii*, *Intestinibacter bartelettii*, *Firimicutes* spp.; (*P* < 0.05, *P_FDR_* < 0.20)].

### Perturbation in microbial metabolites in axSpA patients and healthy subjects

**Figure 4:**
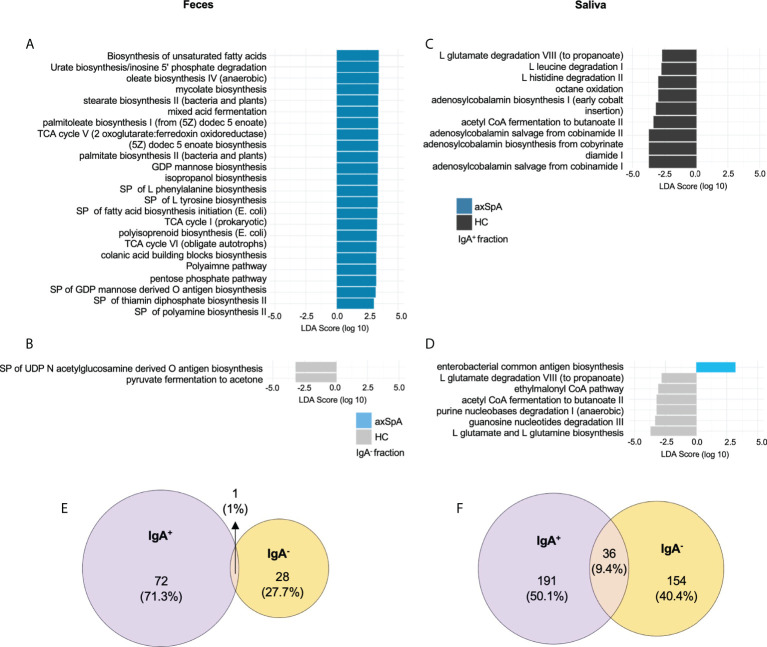
PICRUSt2 pathways and metabolites enriched in axSpA patients and healthy individuals. Linear Discriminant analysis (LDA) effect size analysis showing differentially abundant MetaCyc metabolic pathways between various groups shown in a bar plot in **(A)** IgA enriched and **(B)** IgA depleted fecal fractions comparing axSpA patients with HCs. The bar plots for salivary **(C)** IgA enriched and **(D)** IgA depleted fractions from axSpA patients in comparison with HCs are also shown. Predicted microbial metabolites (KO) in the **(E)** feces and **(F)** saliva. The data for both IgA^+^ and IgA^-^ fractions and their overlap as shown in area proportional Euler plots. Briefly, the numbers represent KOs significantly altered in their LDA score in axSpA patients in comparison with the HC in the IgA^+^ fraction (purple) and IgA^-^ fraction (yellow). MetaCyc pathways and KEGG metabolites (KOs) with a class level alpha<0.05 and subclass alpha<0.05 are considered significant. SP (superpathways), TCA (tricarboxylic acid), GDP (guanosine diphosphate).

To infer the microbial metagenome and metabolic pathways, we employed PICRUSt2, which uses marker gene data (16S sequencing) to predict microbial metabolic potential. Previous inferred microbial metabolic profiling of SpA patients (41) and HLA-B27 TG rats (9) using PICRUST has revealed perturbation of inflammatory pathways associated with disease and/or HLA-B27 status. Therefore, in this manuscript, based on our findings from HLA-B27 TG rats, we further investigated the contribution of IgA^+^ or IgA^-^ fractions in the perturbation of microbial metabolic function. We found multiple metabolic pathways (MetaCyc) significantly increased (alpha<0.05 for class and subclass analysis) in the fecal IgA^+^ fraction of axSpA patients (**Figure 4A**) including biosynthesis of unsaturated fatty acids, isopropanol biosynthesis, polyamine biosynthesis, and pathways for amino acids biosynthesis (phenylalanine, tyrosine), and colonic acid biosynthesis with a decrease in superpathway of UDP-N acetylglucosamine derived O antigen biosynthesis and pyruvate fermentation to acetone in the IgA^-^ fractions (**Figure 4B**). On the contrary, salivary IgA^+^ fractions (**Figures 4C, D**) showed decreased pathways for L glutamate degradation VIII (to propanoate), acetyl CoA fermentation to butanoate II, and degradation of amino acid pathways (leucine, histidine) in axSpA patients as compared with HCs. We also observed alteration in nucleotide biosynthesis pathways (pyrimidine deoxyribonucleotides *de novo* biosynthesis I, 5 aminoimidazole ribonucleotide biosynthesis I, guanosine ribonucleotides *de novo* biosynthesis) in both fecal and salivary fractions (**Supplementary Figures 4A, C**). In the salivary IgA^-^ fraction of axSpA patients, we observed an increase in enterobacterial common antigen biosynthesis, with a decrease in pathways for L glutamate and L glutamine biosynthesis, L glutamate degradation VIII (to propanoate), acetyl CoA fermentation to butanoate II, purine nucleobases degradation I (anaerobic), guanosine nucleotides degradation (**Figures 4C, D**). Additional exploratory analyses of metabolic pathways were also performed (alpha <0.1 for class and alpha 0.05 for subclass analysis), which revealed alteration in pathways for various amino acids biosynthesis, fatty acid biosynthesis, propanoate degradation etc. (**Supplementary Figures 4A–D**).

In addition, LEfSE analysis at the KEGG ortholog (KO) level for predictive genes/metabolite revealed perturbation of 72 predictive genes in the IgA^+^ fraction and 28 genes in the IgA^-^ fraction of axSpA fecal samples, with a minimal overlap of 1 gene (**Figure 4E**). Meanwhile, salivary IgA^+^ and IgA^-^ fractions had higher numbers of predictive genes (191 and 154 respectively) perturbed in axSpA patients, with 36 shared genes between the IgA^+^ and IgA^-^ fractions (**Figure 4F**). However, minimal overlap of the oral and fecal microbial genes was observed (data not shown). In the fecal IgA^+^ fraction, genes from inflammatory pathways such as lipopolysaccharide biosynthesis (K19353, K03271, K02847), glutathione metabolism (K00036, K00033, K00432), oxidative phosphorylation (K00239), and biofilm formation (K01791, K00640) were perturbed. The IgA^-^ fraction showed a decreased abundance of predicted genes from metabolic pathways such as propanoate and (K01035, K01034), and butanoate metabolism (K01035, K01034, K14534) in axSpA patients. The salivary IgA^+^ fraction from axSpA patients had decreased abundance of genes from butanoate (K01907, K17865, K03821, K01692, K01028, K07246, K01640), and propanoate (K01962, K01965, K01026, K00822, K01692) metabolism. Multiple genes for other inflammatory pathways such as tryptophan metabolism (K01692, K04103), oxidative phosphorylation (K08738, K02122, K02121, K02119), flagellar assembly (K02408, K02406, K02407), and glutathione metabolism (K07232) had altered abundances in the IgA^+^ fraction of the saliva samples. We also performed additional exploratory analyses (class analysis alpha < 0.1, subclass analysis alpha < 0.05), which revealed additional KOs belonging to pathways mentioned above (**Supplementary Figures 4E, F**). The predictive metabolites significantly altered in the IgA^+^ and IgA^-^ fractions from fecal and salivary samples are detailed in **Supplementary Table 3**.

## Discussion

In this study, we examined the IgA coating of microbes in the fecal and salivary microbiome from axSpA patients and HCs. To our knowledge, this is the first study to focus on IgA-enriched and depleted microbes and their predictive metabolic contributions in axSpA patients. We found an enrichment of IgA coated microbes in the fecal samples from axSpA patients. Also, the salivary microbiome from axSpA patients had a lower number of IgA coated microbes despite showing significant microbial dysbiosis. Some of these IgA coated microbes showed an inverse correlation with the BASDAI. In addition, predictive microbial function revealed various inflammatory and metabolic pathways perturbed in fecal and salivary samples from axSpA patients. Taken together, our data suggest that IgA selectively marks inflammatory or immune reactive microbes, resulting in an altered microbial community structure and function in axSpA patients.

The present study combining 16S sequencing of the oral and fecal microbiome and the IgA coated microbes proved useful in deciphering whether dysbiotic microbes are differentially coated with IgA. Despite minor changes in the overall microbial diversity and composition in axSpA patients and HCs, we observed a significant change in the IgA coating of oral and fecal microbes in these patients as compared to HCs. Notably, IgA-SEQ revealed many microbes that were not differentially abundant using the 16S sequencing alone, e.g., *Akkermansia*, and members of family *Ruminococcaceae* and *Lachnospiraceae*. These microbes have been shown to be associated with clinical studies in SpA and IBD (12, 42) as well as in experimental models of SpA (7–9). Interestingly, *Akkermansia* has been implicated as a pathogenic microbe in SpA and IBD (12, 42); and a protective microbe in metabolic diseases and obesity studies (43). We also observed other IgA coated microbes in the fecal (e.g., *Lachnospiraceae, Ruminococcaceae*) and salivary (e.g., *Prevotellaceae, Actinobacillus*) samples from axSpA patients, which have been shown to be associated with various spondyloarthropathies (12, 14). Furthermore, in a recent study on IgA coated microbes in CD-SpA patients also reported increased IgA coating of *Escherichia/Shigella*, *Lachnospiraceae* (17), as seen in axSpA patients from our study. Almost 50-70% of axSpA patients have reported to have subclinical gut inflammation (44), which is further supported by increased calprotectin levels reported in SpA patients (45). This could explain the enrichment of *Escherichia/Shigella* similar to that seen in CD-SpA patients (17). Therefore, our study highlights *Akkermansia, Escherichia/Shigella* and members of *Lachnospiraceae, Ruminococcaceae and Prevotellaceae* for eliciting an IgA response which suggests immunologic significance. Since these organisms are able to activate the host immune response as indicated by an IgA response, the targeted micro-organisms should be studied further for functional analysis to determine host-microbe interactions in disease development.

Recent studies have focused on specific microbes as a disease biomarker, patients with AS and SpA have shown correlation of disease activity (BASDAI) with increased relative abundance of *Ruminococcus gnavus* and *Dialister*, respectively in two different studies (12, 13). Other studies have reported gut dysbiosis correlating with the fecal calprotectin levels (6, 46), suggesting that using multiple markers of disease activity (e.g., BASDAI, fecal calprotectin) in future studies may improve correlation between disease and dysbiotic microbiota. Our current study correlates various IgA coated microbes with the disease activity (BASDAI), and has revealed many microbes with negative correlation between IgA coating and BASDAI score in the fecal and saliva fractions. However, a member of class *Clostridiales Family XIII* showed positive correlation between IgA coating and increased BASDAI score, without a significant alteration in its relative abundance in axSpA patients (data not shown). While IgA coated microbes have shown to exacerbate gut inflammation (16, 17), IgA coated microbes from healthy individuals have been shown to protect from gut inflammation (47) and regulates microbial composition (48). Additionally, a gut commensal microbe *Bacteroides fragilis* can alter its cell wall to enhance IgA coating, allowing it to adhere to the gut epithelial cells, and discouraging colonization by potential pathobionts (49). These opposing roles for IgA may be due to the T cell dependent IgA production affected by Th-17 cells or Regulatory T cells (17, 47). Furthermore, our studies correlating microbial relative abundance with dysregulated inflammatory genes in HLA-B27 TG rats revealed that the relative abundance of only a few microbes correlated with disease severity, and most microbes may increase or decrease in response to gut inflammation, and therefore may not be the causal microbes (50). Together, these studies suggest a potential for using IgA coating as a microbial biomarker for disease activity, since many of the microbial perturbations in axSpA patients might be in response to altered gut microenvironment.

Our results demonstrate alterations in the salivary microbial community associated with axSpA patients. While the salivary microbes in axSpA patients showed significant alterations in the microbial community and structure, we observed a smaller number of IgA coated microbes in comparison with the fecal samples. However, there are significant changes in the predictive microbial function in both IgA^+^ and IgA^-^ fractions from saliva samples of axSpA patients. A possible explanation is that perturbation of oral mucosa may be a result of disease instead of driving the disease in axSpA patients.

To evaluate the functional potential of IgA enriched microbes in axSpA patients, we employed PICRUSt2 to predict microbial metabolites altered in IgA^+^ and IgA^-^ fractions in feces and saliva. Despite secretory IgA being the most abundant immunoglobulin produced in our body (51), only 5-10% of total microbes are IgA coated (16, 52). Despite the difference in abundance, most of the microbial metabolic perturbations in the fecal samples were associated with the IgA^+^ fractions, which further highlight their importance in axSpA pathogenesis. We found that most microbial metabolites perturbed in axSpA patients belong to inflammatory pathways [e.g., isopropanol biosynthesis, degradation of amino acid pathways (leucine, histidine, tyrosine), degradation of various sugars (mannan, glucose, xylose)], nucleotide biosynthesis pathways (50, 53) and pathways for short chain fatty acid (SCFA) metabolism (e.g., glutamate degradation VIII (to propanoate), acetyl CoA fermentation to butanoate II) (8, 50, 53). We also found alterations in the biosynthesis of tetrahydrofolate, a cofactor required for the synthesis of amino acids and nucleic acids, which is interesting since we also saw altered biosynthesis of both amino acids and nucleotides in axSpA patients as compared to the HCs. Additionally, alteration in biotin [vitamin B7] biosynthesis pathway is seen in IgA^+^ fractions of fecal samples in axSpA patients. Biotin deficiency was recently reported in IBD patients (54) and mice deficient in biotin have exacerbation in colitis, which is alleviated by biotin supplementation (55). These results show a perturbation in the metabolic biosynthesis and degradation pathways, which could be involved in disease development and progression.

While the pathways for tryptophan biosynthesis were not significantly different, various genes/metabolites in that pathways were significantly altered in axSpA patients in comparison with HCs. Recent studies from Berlinberg and colleagues also show increased gene abundance for various metabolites in the tryptophan pathways in patients with axSpA and axSpA-CD (42). Alterations in tryptophan pathway are also reported by Stoll and colleagues to be associated with pediatric SpA (41). Metabolomic analysis of the plasma samples from AS patients also found a decrease in tryptophan metabolites (56), whereas another study found increase of tryptophan metabolites in AS patients (57). These studies indicate that decrease in tryptophan or increase in tryptophan metabolism might play an important role in AS pathogenesis. Also, multiple genes belonging to SCFA metabolism were perturbed in the saliva and fecal microbiome in axSpA patients. Previous studies by Shao and colleagues (58) have shown that AS and RA patients have decreased production of SCFAs - butanoate and propanoate. In addition, Asquith and colleagues performed fecal metabolomic analysis in HLA-B27 TG rats and found many inflammatory and SCFA metabolism pathways were altered in these animals (59). Gut inflammation in these HLA-B27 TG rats was ameliorated by adding propanoate in their drinking water (59), further emphasizing the protective role of SCFAs. These studies suggest the need for an in-depth analysis of fecal and blood metabolites and their association with AS. Furthermore, metagenomic analysis of the IgA coated microbes will allow studying host-microbe interactions at the species or strain level.

Our study has some limitations. First, we did not observe loss of fecal microbial diversity in axSpA patients in comparison with HCs, which could be due to our small sample size and increased variability due to our cohort being recruited from across the US. Additionally, this may be indicative of axSpA being a true dysbiotic model, instead of a being associated with loss in microbial diversity as reported in IBD (60). For example, a large cohort of patients with juvenile idiopathic arthritis (JIA) (75 JIA patients and 32 controls) did not find significant differences in the alpha or beta diversity of gut microbes when compared to controls (61), despite having significant changes at the level of individual microbes. Another study performed metagenomic sequencing on fecal samples from patients with CD, axSpA, axSpA-CD and healthy controls did not observe any differences in the microbial alpha diversity between these groups (42). Second, our observations were limited to fecal samples, and a more severe microbial dysbiosis may be present in the intestinal mucosal microbiome as shown in our previous study in HLA-B27 TG rats (9). Despite the lack of/smaller changes observed in the microbial composition, we were intrigued by the ability of IgA-SEQ to reflect the immunological impact of these microbes.

One more limitation is the inherent microbial diversity which complicates microbiome comparisons between patients and healthy individuals. We are still in the phase of exploring the microbiome’s relationship to human health. Therefore, microbiome analyses test several hypotheses to identify disease specific alterations, which inflates the chance of false positive findings. To account for this, while also not excluding interesting observations for researchers in the similar field, we report both p-values (*P*) and adjusted p-values (*P_FDR_*). Similarly, for the inferred microbial metabolic pathway analysis, we performed a stringent and relaxed cutoff as done in our previous microbiome studies (9).

Another limitation is the use of biologics in almost 50% of the axSpA patients in our stool and saliva cohorts. Treatment with various biologics have shown to be associated with alteration in the microbial community. In SpA patients, treatment with IL-17 inhibitors correlated with features of sub-clinical gut inflammation, associated with changes in the relative abundance of certain microbial and fungal taxa (62). Conversely, TNF inhibitors have shown to shift the microbial community similar to the healthy controls (63), which may also account for the lack of alpha and beta diversity measures in axSpA patients by using 16S sequencing alone. Additionally, 47% of axSpA patients were on some form of NSAIDs as compared to 21% NSAID use in HCs. Studies on AS patients have shown association of NSAID use with alteration of the gut microbial community (64), increased levels of fecal calprotectin (46), as well as with increased number of bacterial species (65). However, many of these patients still had an active disease as measured by the BASDAI scores, and our studies revealed distinct IgA coated microbes associated with axSpA despite the limitation of biologics and NSAIDs used in our cohort. Further studies with increased sample size will allow us to dissect the effect of biologic and NSAIDs use on the microbial community structure in axSpA patients. However, our essential observation that IgA-coating of bacteria identifies a potentially pathogenic subset of the microbiome is supported by multiple observations reported herein as well as by prior publications (16, 17).

The etiology of axSpA is complex involving an interplay of genetic, environmental and microbial factors in disease development and severity. Our results highlight the value of identifying the IgA coated microbes, which are immune-targeted microbes, based on 16S sequencing of the IgA^+^ and IgA^-^ fractions. Further in-depth examination of IgA coated microbes using shotgun metagenomic sequencing of IgA^+^ and IgA^-^ fractions to determine potentially inflammatory microbes at a strain specific level is warranted. Our results suggest that IgA coated microbes in axSpA patients indicate a stimulated immune response to gut commensal microbes, which might contribute to the development of axSpA. Future studies to determine the mechanistic links between IgA coated microbiota and disease will further highlight the functional implications of IgA coated bacteria in axSpA.

## Data availability statement

The data presented in the study are deposited in Sequence Read Archive (SRA), accession number under bioproject PRJNA873789.

## Ethics statement

This study was reviewed and approved by OHSU Institutional Review Board. The patients/participants provided their written informed consent to participate in this study.

## Author contributions

MA (†) was involved in the study design and data generation for the current manuscript. TG, TM, JR and LK had access to all the data and patient records and share the responsibly for data integrity and its accurate analysis. All authors contributed to the article and approved the submitted version.

## Funding

This study was supported by NIH R01EY029266 (JR-PI), NIH P30EY010572 (Casey Eye Institute), Research to Prevent Blindness (Casey Eye Institute), the Rheumatology Research Foundation, the Spondylitis Association of America (Jane Bruckel ECI Award to TG), NIH K01DK116706 (LK), NIH T32HL083808 (IL) and NIH National Library of Medicine Training Grant T15-LM007088 (JN). JR receives support from the Grandmaison Fund for Autoimmunity Research, the Stan and Madelle Rosenfeld Family Trust, and the William and Mary Bauman Foundation.

## Acknowledgments

The authors wish to thank Lindsey Watson and Puthyda Keath for human subjects’ recruitment and sample processing, Manny Rodriguez for laboratory assistance, Erin Dahl for help with data visualization, and the Spondylitis Association of America for allowing us to advertise the study to its members.

## Conflict of interest

The authors declare that the research was conducted in the absence of any commercial or financial relationships that could be construed as a potential conflict of interest.

## Publisher’s note

All claims expressed in this article are solely those of the authors and do not necessarily represent those of their affiliated organizations, or those of the publisher, the editors and the reviewers. Any product that may be evaluated in this article, or claim that may be made by its manufacturer, is not guaranteed or endorsed by the publisher.
