# Supplementary figures and images for: Axial spondyloarthritis patients have altered mucosal IgA response to oral and fecal microbiota
Source: Front Immunol. 2022 Sep 28;13:965634. doi: 10.3389/fimmu.2022.965634 (PMC9556278; doi:10.3389/fimmu.2022.965634)

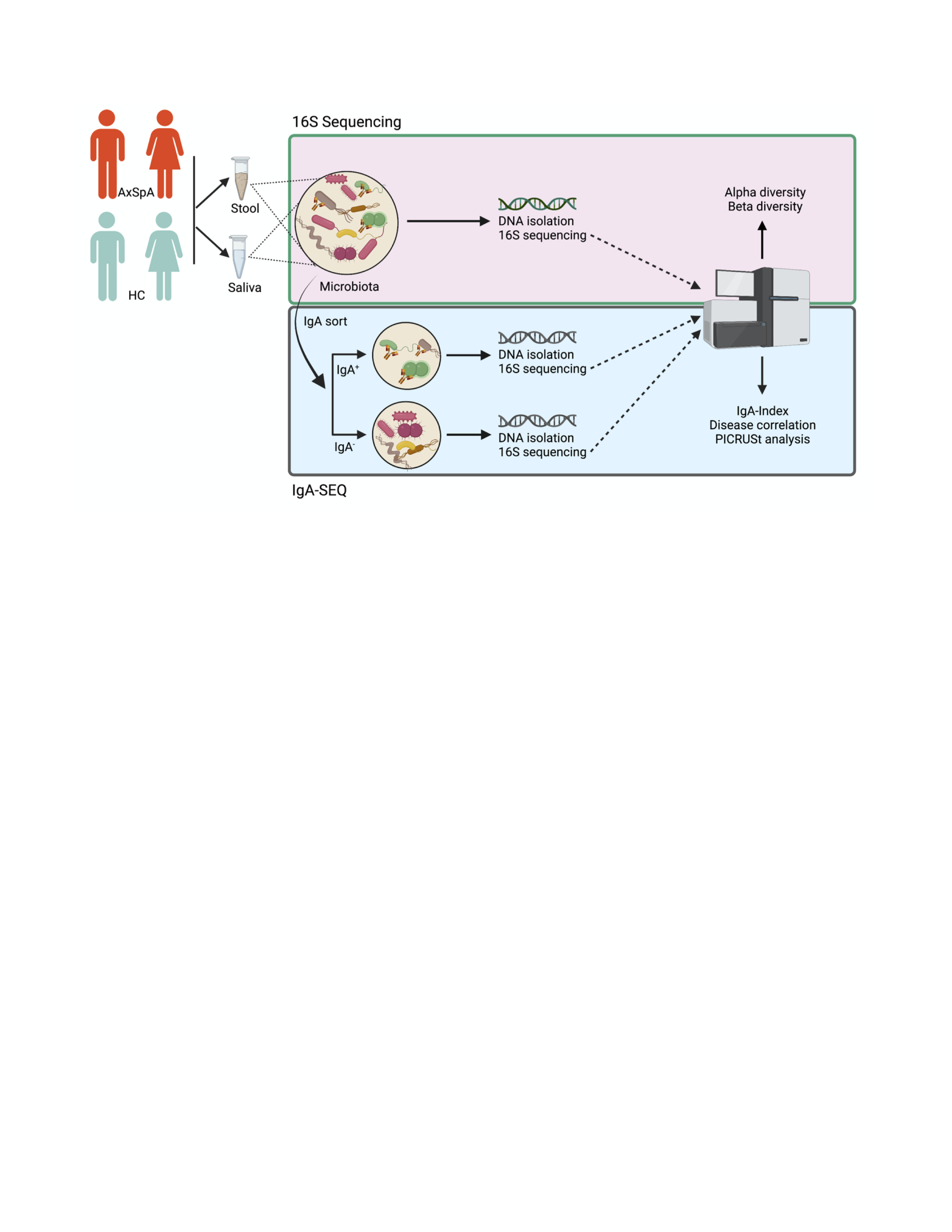

Supplement: Supplementary file 1 [file Image_1.tiff]

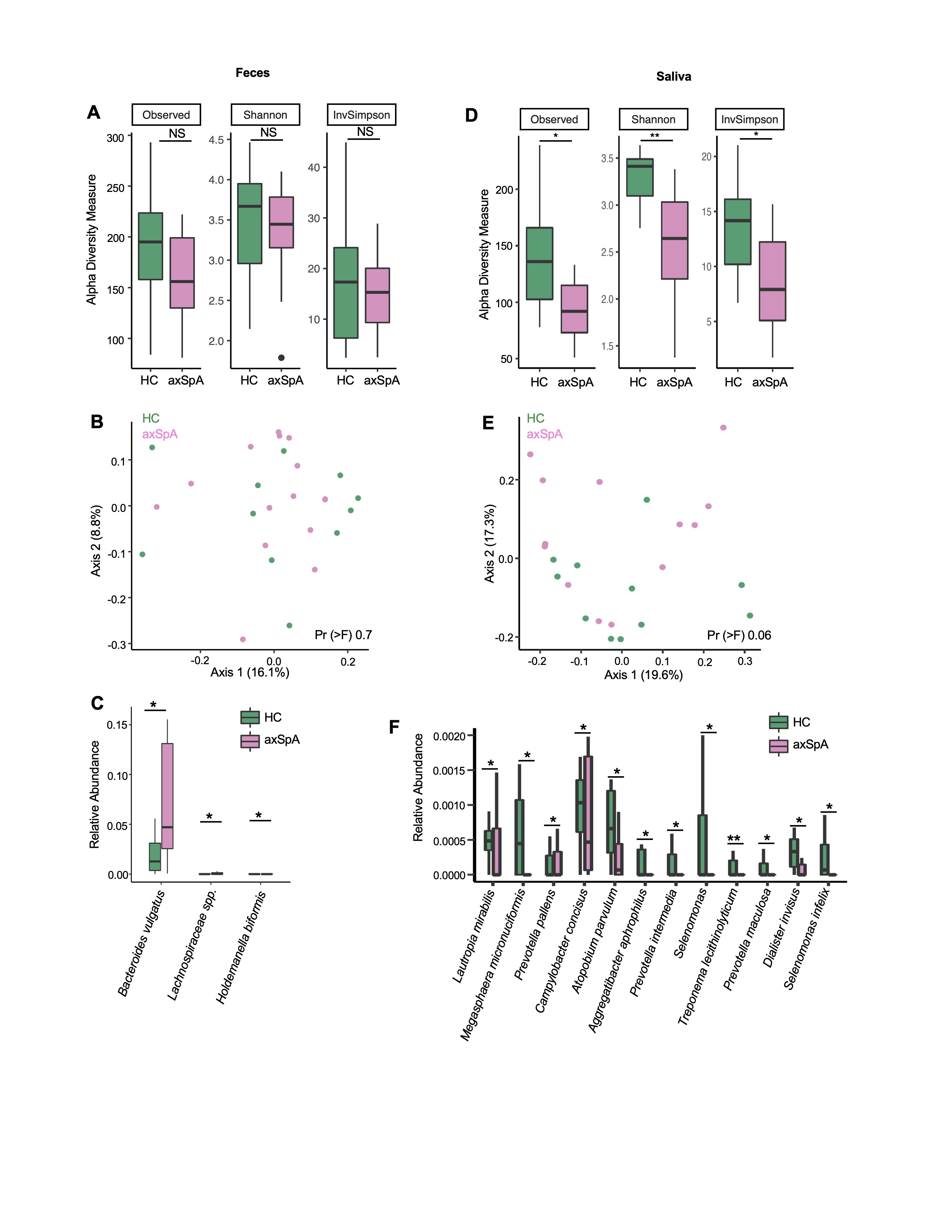

Supplement: Supplementary file 2 [file Image_2.tiff]

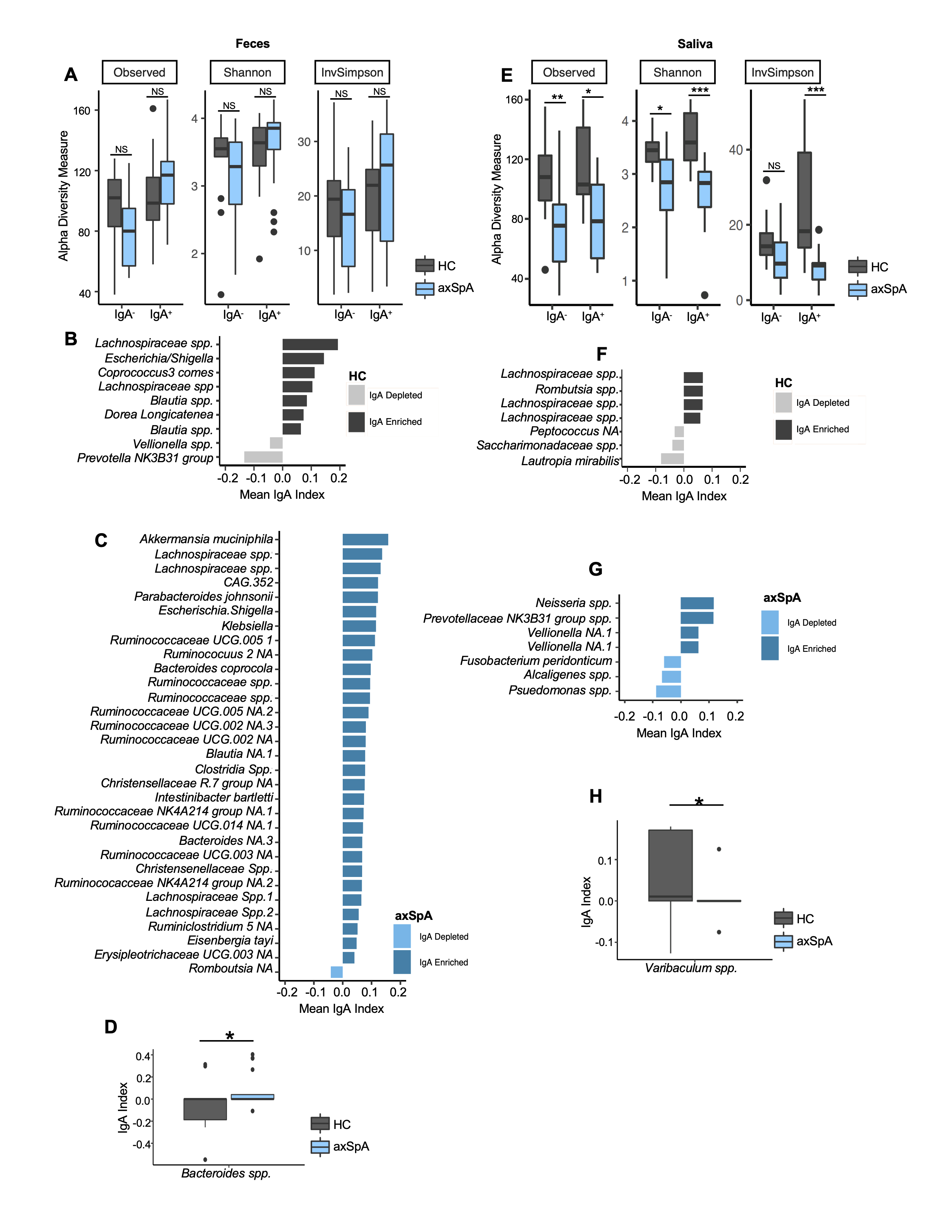

Supplement: Supplementary file 3 [file Image_3.tiff]

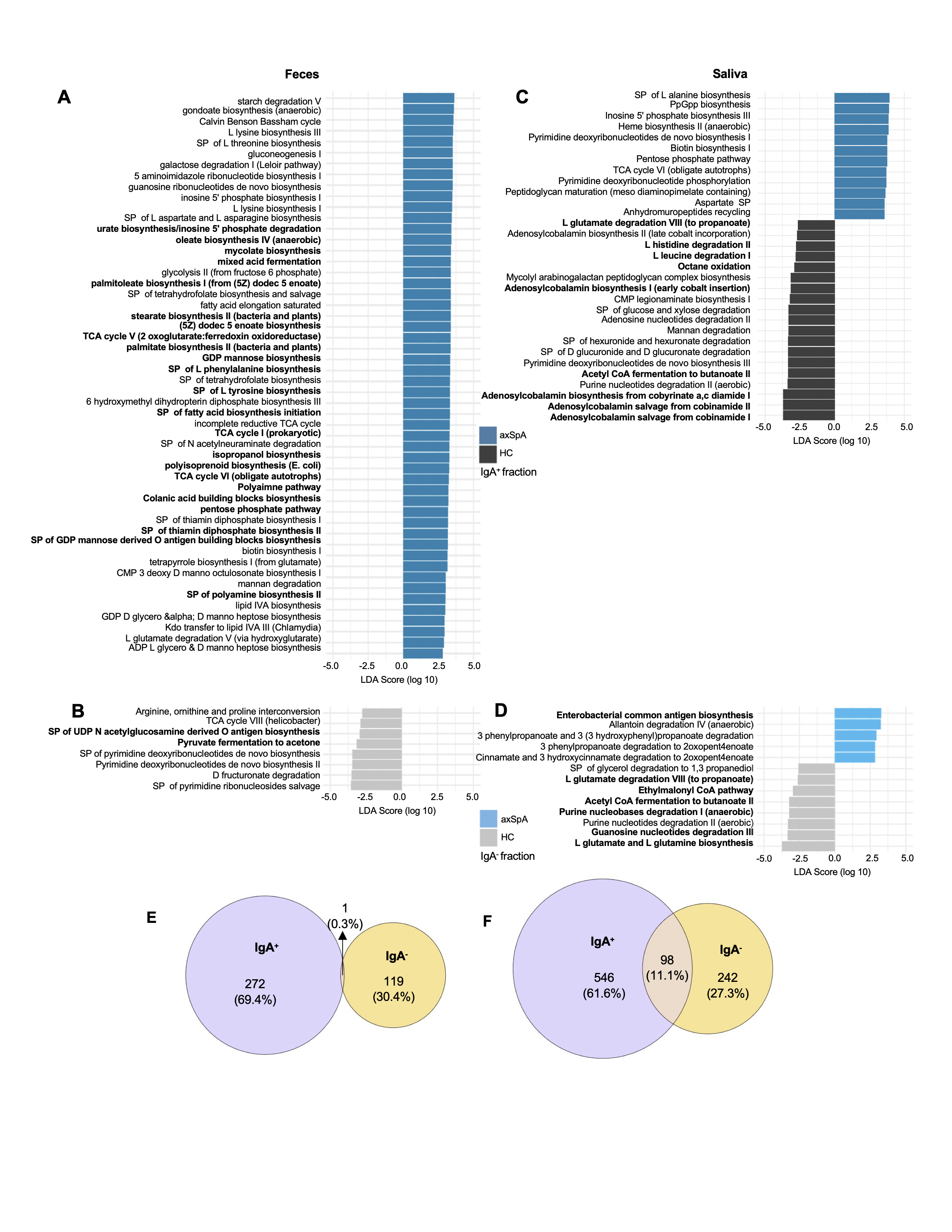

Supplement: Supplementary file 4 [file Image_4.tiff]
