# Supplementary material for: Axial spondyloarthritis patients have altered mucosal IgA response to oral and fecal microbiota
Source: Front Immunol. 2022 Sep 28;13:965634. doi: 10.3389/fimmu.2022.965634 (PMC9556278; doi:10.3389/fimmu.2022.965634)
Supplement: Supplementary file 5 [file Table_1.docx]

**Supplementary Table 1:** Location and recruitment status of axSpA patients and healthy individuals at the time of sample collection for stool and saliva cohort

|  | **AxSpA Local^*^** | **AxSpA SAA^#^** | **HC Local** | **HC SAA** |
| --- | --- | --- | --- | --- |
| **Stool cohort** | 8 Oregon  1 Washington | 3 California  1 Florida  1 North Carolina  1 New Mexico  1 Oregon  1 Washington | 7 Oregon | 1 Alaska  1 California  1 Connecticut  1 Massachusetts  2 Texas  1 Washington |
| **Saliva cohort** | 3 Oregon | 3 California  1 Florida  1 Idaho  2 Maryland  1 Minnesota  1 New Mexico  1 Texas  1 Washington | 9 Oregon | 1 Oregon  1 California  1 Wisconsin |

*Local: Rheumatology or Ophthalmology clinics at OHSU, or Devers Eye Clinic, Portland, OR; or flyer posted in clinics; or word-of-mouth

^#^SAA: Spondylitis Association of America study advertisement on website or newsletter sent to members
