# Supplementary material for: Axial spondyloarthritis patients have altered mucosal IgA response to oral and fecal microbiota
Source: Front Immunol. 2022 Sep 28;13:965634. doi: 10.3389/fimmu.2022.965634 (PMC9556278; doi:10.3389/fimmu.2022.965634)
Supplement: Supplementary file 6 [file Table_2.docx]

**Supplementary Table 2: Genus and ASV level microbial dysbiosis in the fecal and salivary samples from axSpA patients in comparison with the healthy controls**

| **Genus Level** | | | |
| --- | --- | --- | --- |
| **Fecal Fraction: (Figure 1C)** | | | |
|  |  | ***Pval*** | ***P_FDR_*** |
|  | *Fournierella* | 0.02 | 0.79 |
|  | *Anaerofilum* | 0.03 | 0.79 |
|  | *Faecalitalea* | 0.03 | 0.79 |
| **Salivary Fraction: (Figure 1F)** | | | |
|  |  | ***Pval*** | ***P_FDR_*** |
|  | *F0058* | 0.04 | 0.52 |
|  | *Corynebacterium* | 0.01 | 0.41 |
|  | *Atopobium* | 0.01 | 0.41 |
|  | *Treponema* | 0.03 | 0.52 |
|  | *Ruminococcaceae_UCG-014* | 0.02 | 0.51 |
|  | *Campylobacter* | 0.01 | 0.41 |
|  | *Aggregatibacter* | 0.01 | 0.41 |
|  | *Oribacterium* | 0.03 | 0.52 |
|  | *Selenomonas* | 0.03 | 0.52 |
|  | *Megasphaera* | 0.01 | 0.41 |
| **ASV Level** | | | |
| **Fecal Fraction: (Supplementary Figure 2C)** | | | |
|  |  | ***Pval*** | ***P_FDR_*** |
|  | *Bacteroides vulgatus* | 0.02 | 0.74 |
|  | *Lachnospiraceae spp.* | 0.005 | 0.74 |
|  | *Holdemanella biformis* | 0.04 | 0.74 |
| **Salivary Fraction: (Supplementary Figure 2F)** | | | |
|  |  | ***Pval*** | ***P_FDR_*** |
|  | *Selenomonas_3 infelix* | 0.02 | 0.57 |
|  | *Dialister invisus* | 0.01 | 0.57 |
|  | *Prevotella maculosa* | 0.03 | 0.57 |
|  | *Treponema_2 lecithinolyticum* | 0.01 | 0.57 |
|  | *NA sputigena_g__Selenomonas* | 0.03 | 0.57 |
|  | *Prevotella intermedia* | 0.02 | 0.57 |
|  | *Aggregatibacter aphrophilus* | 0.02 | 0.57 |
|  | *Atopobium parvulum* | 0.01 | 0.57 |
|  | *Campylobacter concisus* | 0.02 | 0.57 |
|  | *Prevotella pallens* | 0.01 | 0.57 |
|  | *Megasphaera micronuciformis* | 0.02 | 0.57 |
|  | *Lautropia mirabilis* | 0.03 | 0.57 |
