# Supplementary material for: Axial spondyloarthritis patients have altered mucosal IgA response to oral and fecal microbiota
Source: Front Immunol. 2022 Sep 28;13:965634. doi: 10.3389/fimmu.2022.965634 (PMC9556278; doi:10.3389/fimmu.2022.965634)
Supplement: Supplementary file 7 [file Table_3.docx]

**Supplementary Table 3**. Predictive microbial metabolites (KEGG level 4) significantly altered in axSpA patients in comparison with HCs from IgA+ and IgA- fractions of feces and saliva samples.

| **Feces_AxSpA vs HC_IgA+_a0.01_w0.05_l2_minc.default.res.sig.txt** | | | | |
| --- | --- | --- | --- | --- |
| **Level D (KO) _Description** | **KO** | **Class with highest mean** | **Log LDA score** | **p-value (KW for class)** |
| tcyL;_L-cystine_transport_system_permease_protein | K16958 | AxSpA | 2.484 | 0.0006 |
| E3.2.1.8,_xynA;_endo-1,4-beta-xylanase_[EC:3.2.1.8] | K01181 | AxSpA | 2.833 | 0.0015 |
| HMOX1;_heme_oxygenase_1_[EC:1.14.14.18] | K00510 | AxSpA | 2.829 | 0.0020 |
| cphA;_cyanophycin_synthetase_[EC:6.3.2.29_6.3.2.30] | K03802 | AxSpA | 2.829 | 0.0020 |
| lonB;_ATP-dependent_Lon_protease_[EC:3.4.21.53] | K04076 | AxSpA | 2.829 | 0.0020 |
| phaF;_multicomponent_K+:H+_antiporter_subunit_F | K05563 | AxSpA | 2.829 | 0.0020 |
| phaG;_multicomponent_K+:H+_antiporter_subunit_G | K05564 | AxSpA | 2.829 | 0.0020 |
| yfkQ;_spore_germination_protein | K06307 | AxSpA | 2.829 | 0.0020 |
| spoVR;_stage_V_sporulation_protein_R | K06415 | AxSpA | 2.829 | 0.0020 |
| entB;_probable_enterotoxin_B | K11060 | AxSpA | 2.829 | 0.0020 |
| cphB;_cyanophycinase_[EC:3.4.15.6] | K13282 | AxSpA | 2.829 | 0.0020 |
| HYDIN;_hydrocephalus-inducing_protein | K17570 | AxSpA | 2.829 | 0.0020 |
| K10120,_msmE;_fructooligosaccharide_transport_system_substrate-binding_protein | K10120 | AxSpA | 2.611 | 0.0025 |
| tcyK;_L-cystine_transport_system_substrate-binding_protein | K16957 | AxSpA | 2.447 | 0.0033 |
| tcyM;_L-cystine_transport_system_permease_protein | K16959 | AxSpA | 2.433 | 0.0043 |
| SLC13A2_3_5;_solute_carrier_family_13_(sodium-dependent_dicarboxylate_transporter),_member_2/3/5 | K14445 | AxSpA | 2.934 | 0.0043 |
| mexK;_multidrug_efflux_pump | K18303 | AxSpA | 2.503 | 0.0044 |
| gpx,_btuE,_bsaA;_glutathione_peroxidase_[EC:1.11.1.9] | K00432 | AxSpA | 3.211 | 0.0055 |
| pabAB;_para-aminobenzoate_synthetase_[EC:2.6.1.85] | K13950 | AxSpA | 2.646 | 0.0062 |
| cld;_chlorite_dismutase_[EC:1.13.11.49] | K09162 | AxSpA | 2.876 | 0.0067 |
| nrfA;_nitrite_reductase_(cytochrome_c-552)_[EC:1.7.2.2] | K03385 | AxSpA | 2.787 | 0.0078 |
| fabB;_3-oxoacyl-[acyl-carrier-protein]_synthase_I_[EC:2.3.1.41] | K00647 | AxSpA | 3.059 | 0.0088 |
| abnA;_arabinan_endo-1,5-alpha-L-arabinosidase_[EC:3.2.1.99] | K06113 | AxSpA | 3.295 | 0.0088 |
| tcyN;_L-cystine_transport_system_ATP-binding_protein_[EC:7.4.2.1] | K16960 | AxSpA | 2.511 | 0.0111 |
| E4.2.1.2B,_fumC,_FH;_fumarate_hydratase,_class_II_[EC:4.2.1.2] | K01679 | AxSpA | 2.751 | 0.0124 |
| ATOX1,_ATX1,_copZ,_golB;_copper_chaperone | K07213 | AxSpA | 2.674 | 0.0124 |
| wecB;_UDP-N-acetylglucosamine_2-epimerase_(non-hydrolysing)_[EC:5.1.3.14] | K01791 | AxSpA | 3.542 | 0.0155 |
| SMARCAL1,_HARP;_SWI/SNF-related_matrix-associated_actin-dependent_regulator_of_chromatin_subfamily_A-like_protein_1_[EC:3.6.4.12] | K14440 | AxSpA | 2.476 | 0.0157 |
| K02475;_two-component_system,_CitB_family,_response_regulator | K02475 | AxSpA | 2.516 | 0.0163 |
| adc;_acetoacetate_decarboxylase_[EC:4.1.1.4] | K01574 | AxSpA | 2.476 | 0.0172 |
| araN;_arabinosaccharide_transport_system_substrate-binding_protein | K17234 | AxSpA | 2.476 | 0.0172 |
| araP;_arabinosaccharide_transport_system_permease_protein | K17235 | AxSpA | 2.476 | 0.0172 |
| tarJ;_ribitol-5-phosphate_2-dehydrogenase_(NADP+)_[EC:1.1.1.405] | K05352 | AxSpA | 2.988 | 0.0185 |
| aguA;_alpha-glucuronidase_[EC:3.2.1.139] | K01235 | AxSpA | 2.908 | 0.0192 |
| eptC;_heptose-I-phosphate_ethanolaminephosphotransferase_[EC:2.7.8.-] | K19353 | AxSpA | 2.915 | 0.0192 |
| pgpA;_phosphatidylglycerophosphatase_A_[EC:3.1.3.27] | K01095 | AxSpA | 2.904 | 0.0213 |
| terD;_tellurium_resistance_protein_TerD | K05795 | AxSpA | 2.736 | 0.0213 |
| dinD;_DNA-damage-inducible_protein_D | K14623 | AxSpA | 3.052 | 0.0213 |
| blt;_MFS_transporter,_DHA1_family,_multidrug_resistance_protein | K08153 | AxSpA | 2.709 | 0.0230 |
| pcp;_pyroglutamyl-peptidase_[EC:3.4.19.3] | K01304 | AxSpA | 2.504 | 0.0237 |
| PPOX,_hemY;_protoporphyrinogen/coproporphyrinogen_III_oxidase_[EC:1.3.3.4_1.3.3.15] | K00231 | AxSpA | 2.695 | 0.0262 |
| tag;_DNA-3-methyladenine_glycosylase_I_[EC:3.2.2.20] | K01246 | AxSpA | 3.116 | 0.0262 |
| manA,_MPI;_mannose-6-phosphate_isomerase_[EC:5.3.1.8] | K01809 | AxSpA | 3.255 | 0.0290 |
| dacB;_serine-type_D-Ala-D-Ala_carboxypeptidase/endopeptidase_(penicillin-binding_protein_4)_[EC:3.4.16.4_3.4.21.-] | K07259 | AxSpA | 3.124 | 0.0290 |
| E1.1.1.67,_mtlK;_mannitol_2-dehydrogenase_[EC:1.1.1.67] | K00045 | AxSpA | 2.351 | 0.0311 |
| BLMH,_pepC;_bleomycin_hydrolase_[EC:3.4.22.40] | K01372 | AxSpA | 3.179 | 0.0321 |
| xylS,_yicI;_alpha-D-xyloside_xylohydrolase_[EC:3.2.1.177] | K01811 | AxSpA | 3.499 | 0.0321 |
| proX;_glycine_betaine/proline_transport_system_substrate-binding_protein | K02002 | AxSpA | 2.553 | 0.0321 |
| tolA;_colicin_import_membrane_protein | K03646 | AxSpA | 2.824 | 0.0321 |
| hipA;_serine/threonine-protein_kinase_HipA_[EC:2.7.11.1] | K07154 | AxSpA | 3.448 | 0.0321 |
| aspA;_aspartate_ammonia-lyase_[EC:4.3.1.1] | K01744 | AxSpA | 3.209 | 0.0354 |
| K02476;_two-component_system,_CitB_family,_sensor_kinase_[EC:2.7.13.3] | K02476 | AxSpA | 2.594 | 0.0354 |
| cyaB;_adenylate_cyclase,_class_2_[EC:4.6.1.1] | K05873 | AxSpA | 2.741 | 0.0373 |
| cpo;_non-heme_chloroperoxidase_[EC:1.11.1.10] | K00433 | AxSpA | 2.321 | 0.0390 |
| treR2,_treR;_GntR_family_transcriptional_regulator,_trehalose_operon_transcriptional_repressor | K03486 | AxSpA | 2.533 | 0.0390 |
| fic;_cell_filamentation_protein | K04095 | AxSpA | 3.182 | 0.0390 |
| K09940;_uncharacterized_protein | K09940 | HC | 2.285 | 0.0409 |
| epsG;_transmembrane_protein_EpsG | K19419 | AxSpA | 2.613 | 0.0411 |
| cbiL;_nickel_transport_protein | K16915 | AxSpA | 2.426 | 0.0428 |
| ltrA;_RNA-directed_DNA_polymerase_[EC:2.7.7.49] | K00986 | AxSpA | 2.870 | 0.0429 |
| assT;_arylsulfate_sulfotransferase_[EC:2.8.2.22] | K01023 | AxSpA | 2.264 | 0.0429 |
| E3.2.1.4;_endoglucanase_[EC:3.2.1.4] | K01179 | AxSpA | 3.476 | 0.0429 |
| lemA;_LemA_protein | K03744 | AxSpA | 3.179 | 0.0429 |
| ihfA,_himA;_integration_host_factor_subunit_alpha | K04764 | AxSpA | 2.737 | 0.0429 |
| pqqL;_zinc_protease_[EC:3.4.24.-] | K07263 | AxSpA | 3.098 | 0.0429 |
| acm;_lysozyme | K07273 | AxSpA | 3.284 | 0.0429 |
| dptF;_DNA_phosphorothioation-dependent_restriction_protein_DptF | K19173 | AxSpA | 2.391 | 0.0446 |
| waaL,_rfaL;_O-antigen_ligase_[EC:2.4.1.-] | K02847 | AxSpA | 2.602 | 0.0472 |
| gmhA,_lpcA;_D-sedoheptulose_7-phosphate_isomerase_[EC:5.3.1.28] | K03271 | AxSpA | 2.989 | 0.0472 |
| lctP;_lactate_permease | K03303 | AxSpA | 2.698 | 0.0472 |
| fklB;_FKBP-type_peptidyl-prolyl_cis-trans_isomerase_FklB_[EC:5.2.1.8] | K03773 | AxSpA | 3.463 | 0.0472 |
| K07217;_Mn-containing_catalase | K07217 | AxSpA | 2.396 | 0.0472 |
| K09155;_uncharacterized_protein | K09155 | AxSpA | 2.768 | 0.0472 |
| fitB;_toxin_FitB_[EC:3.1.-.-] | K07062 | AxSpA | 2.528 | 0.0502 |
| K07025;_putative_hydrolase_of_the_HAD_superfamily | K07025 | AxSpA | 3.759 | 0.0518 |
| mcrB;_5-methylcytosine-specific_restriction_enzyme_B_[EC:3.1.21.-] | K07452 | AxSpA | 2.531 | 0.0518 |
| vanRB,_vanR,_vanRD;_two-component_system,_OmpR_family,_response_regulator_VanR | K18344 | AxSpA | 2.605 | 0.0518 |
| vanSB,_vanS,_vanSD;_two-component_system,_OmpR_family,_sensor_histidine_kinase_VanS_[EC:2.7.13.3] | K18345 | AxSpA | 2.596 | 0.0518 |
| ihk;_two-component_system,_OmpR_family,_sensor_kinase_Ihk_[EC:2.7.13.3] | K18986 | AxSpA | 2.526 | 0.0518 |
| sdhA,_frdA;_succinate_dehydrogenase_/_fumarate_reductase,_flavoprotein_subunit_[EC:1.3.5.1_1.3.5.4] | K00239 | AxSpA | 3.124 | 0.0567 |
| lip,_TGL2;_triacylglycerol_lipase_[EC:3.1.1.3] | K01046 | AxSpA | 2.375 | 0.0567 |
| araA;_L-arabinose_isomerase_[EC:5.3.1.4] | K01804 | AxSpA | 3.218 | 0.0567 |
| waaF,_rfaF;_heptosyltransferase_II_[EC:2.4.-.-] | K02843 | AxSpA | 2.793 | 0.0567 |
| TC.KEF;_monovalent_cation:H+_antiporter-2,_CPA2_family | K03455 | AxSpA | 3.081 | 0.0567 |
| ftsI;_cell_division_protein_FtsI_(penicillin-binding_protein_3)_[EC:3.4.16.4] | K03587 | AxSpA | 3.138 | 0.0567 |
| eta;_exfoliative_toxin_A/B | K11041 | AxSpA | 2.300 | 0.0567 |
| tcyC,_yecC;_L-cystine_transport_system_ATP-binding_protein_[EC:7.4.2.1] | K10010 | AxSpA | 2.552 | 0.0621 |
| G6PD,_zwf;_glucose-6-phosphate_1-dehydrogenase_[EC:1.1.1.49_1.1.1.363] | K00036 | AxSpA | 2.953 | 0.0621 |
| cysE;_serine_O-acetyltransferase_[EC:2.3.1.30] | K00640 | AxSpA | 3.549 | 0.0621 |
| cpdB;_2',3'-cyclic-nucleotide_2'-phosphodiesterase_/_3'-nucleotidase_[EC:3.1.4.16_3.1.3.6] | K01119 | AxSpA | 3.062 | 0.0621 |
| cynT,_can;_carbonic_anhydrase_[EC:4.2.1.1] | K01673 | AxSpA | 2.950 | 0.0621 |
| ABC.X2.A;_putative_ABC_transport_system_ATP-binding_protein | K02068 | AxSpA | 2.918 | 0.0621 |
| ftnA,_ftn;_ferritin_[EC:1.16.3.2] | K02217 | AxSpA | 3.213 | 0.0621 |
| TC.OOP;_OmpA-OmpF_porin,_OOP_family | K03286 | AxSpA | 2.834 | 0.0621 |
| htpX;_heat_shock_protein_HtpX_[EC:3.4.24.-] | K03799 | AxSpA | 2.890 | 0.0621 |
| sotB;_MFS_transporter,_DHA1_family,_L-arabinose/isopropyl-beta-D-thiogalactopyranoside_export_protein | K08159 | AxSpA | 2.545 | 0.0621 |
| fno;_8-hydroxy-5-deazaflavin:NADPH_oxidoreductase_[EC:1.5.1.40] | K06988 | AxSpA | 2.898 | 0.0628 |
| coxM,_cutM;_aerobic_carbon-monoxide_dehydrogenase_medium_subunit_[EC:1.2.5.3] | K03519 | AxSpA | 2.044 | 0.0646 |
| PGD,_gnd,_gntZ;_6-phosphogluconate_dehydrogenase_[EC:1.1.1.44_1.1.1.343] | K00033 | AxSpA | 2.956 | 0.0679 |
| pdxA;_4-hydroxythreonine-4-phosphate_dehydrogenase_[EC:1.1.1.262] | K00097 | AxSpA | 3.071 | 0.0679 |
| E2.4.1.20;_cellobiose_phosphorylase_[EC:2.4.1.20] | K00702 | AxSpA | 2.978 | 0.0679 |
| manC,_cpsB;_mannose-1-phosphate_guanylyltransferase_[EC:2.7.7.13] | K00971 | AxSpA | 3.314 | 0.0679 |
| iadA;_beta-aspartyl-dipeptidase_(metallo-type)_[EC:3.4.19.-] | K01305 | AxSpA | 2.688 | 0.0679 |
| psd,_PISD;_phosphatidylserine_decarboxylase_[EC:4.1.1.65] | K01613 | AxSpA | 3.232 | 0.0679 |
| asnA;_aspartate--ammonia_ligase_[EC:6.3.1.1] | K01914 | AxSpA | 3.398 | 0.0679 |
| ABC.X2.P;_putative_ABC_transport_system_permease_protein | K02069 | AxSpA | 2.972 | 0.0679 |
| TSTA3,_fcl;_GDP-L-fucose_synthase_[EC:1.1.1.271] | K02377 | AxSpA | 3.076 | 0.0679 |
| lacI,_galR;_LacI_family_transcriptional_regulator | K02529 | AxSpA | 4.124 | 0.0679 |
| pabC;_4-amino-4-deoxychorismate_lyase_[EC:4.1.3.38] | K02619 | AxSpA | 3.023 | 0.0679 |
| dps;_starvation-inducible_DNA-binding_protein | K04047 | AxSpA | 2.961 | 0.0679 |
| phnX;_phosphonoacetaldehyde_hydrolase_[EC:3.11.1.1] | K05306 | AxSpA | 2.706 | 0.0679 |
| rluA;_tRNA_pseudouridine32_synthase_/_23S_rRNA_pseudouridine746_synthase_[EC:5.4.99.28_5.4.99.29] | K06177 | AxSpA | 3.008 | 0.0679 |
| ppnN;_pyrimidine/purine-5'-nucleotide_nucleosidase_[EC:3.2.2.10_3.2.2.-] | K06966 | AxSpA | 3.143 | 0.0679 |
| nadX,_ASPDH;_aspartate_dehydrogenase_[EC:1.4.1.21] | K06989 | AxSpA | 2.552 | 0.0679 |
| K07098;_uncharacterized_protein | K07098 | AxSpA | 3.538 | 0.0679 |
| K07133;_uncharacterized_protein | K07133 | AxSpA | 3.937 | 0.0679 |
| recT;_recombination_protein_RecT | K07455 | AxSpA | 2.387 | 0.0679 |
| togB;_oligogalacturonide_transport_system_substrate-binding_protein | K10192 | AxSpA | 2.421 | 0.0679 |
| yafQ;_mRNA_interferase_YafQ_[EC:3.1.-.-] | K19157 | AxSpA | 2.827 | 0.0679 |
| bcrC;_undecaprenyl-diphosphatase_[EC:3.6.1.27] | K19302 | AxSpA | 3.498 | 0.0679 |
| dasC;_N,N'-diacetylchitobiose_transport_system_permease_protein | K17331 | AxSpA | 2.441 | 0.0707 |
| E3.2.1.58;_glucan_1,3-beta-glucosidase_[EC:3.2.1.58] | K01210 | AxSpA | 2.384 | 0.0708 |
| pdaD;_arginine_decarboxylase_[EC:4.1.1.19] | K02626 | AxSpA | 2.786 | 0.0720 |
| PGLS,_pgl,_devB;_6-phosphogluconolactonase_[EC:3.1.1.31] | K01057 | AxSpA | 2.824 | 0.0741 |
| gmd,_GMDS;_GDPmannose_4,6-dehydratase_[EC:4.2.1.47] | K01711 | AxSpA | 3.156 | 0.0741 |
| dnaB;_replicative_DNA_helicase_[EC:3.6.4.12] | K02314 | AxSpA | 3.504 | 0.0741 |
| dnaQ;_DNA_polymerase_III_subunit_epsilon_[EC:2.7.7.7] | K02342 | AxSpA | 3.358 | 0.0741 |
| menD;_2-succinyl-5-enolpyruvyl-6-hydroxy-3-cyclohexene-1-carboxylate_synthase_[EC:2.2.1.9] | K02551 | AxSpA | 2.985 | 0.0741 |
| ahpF;_NADH-dependent_peroxiredoxin_subunit_F_[EC:1.8.1.-] | K03387 | AxSpA | 2.975 | 0.0741 |
| wcaF;_putative_colanic_acid_biosynthesis_acetyltransferase_WcaF_[EC:2.3.1.-] | K03818 | AxSpA | 2.308 | 0.0741 |
| moxR;_MoxR-like_ATPase_[EC:3.6.3.-] | K03924 | AxSpA | 3.471 | 0.0741 |
| cbpA;_curved_DNA-binding_protein | K05516 | AxSpA | 2.993 | 0.0741 |
| crcB,_FEX;_fluoride_exporter | K06199 | AxSpA | 3.132 | 0.0741 |
| hddA;_D-glycero-alpha-D-manno-heptose-7-phosphate_kinase_[EC:2.7.1.168] | K07031 | AxSpA | 2.696 | 0.0741 |
| SAM50,_TOB55,_bamA;_outer_membrane_protein_insertion_porin_family | K07277 | AxSpA | 3.049 | 0.0741 |
| tri;_tricorn_protease_[EC:3.4.21.-] | K08676 | AxSpA | 2.592 | 0.0741 |
| yhhQ;_queuosine_precursor_transporter | K09125 | AxSpA | 3.020 | 0.0741 |
| thrA;_bifunctional_aspartokinase_/_homoserine_dehydrogenase_1_[EC:2.7.2.4_1.1.1.3] | K12524 | AxSpA | 2.993 | 0.0741 |
| MAN;_mannan_endo-1,4-beta-mannosidase_[EC:3.2.1.78] | K19355 | AxSpA | 2.728 | 0.0741 |
| TC.DASS;_divalent_anion:Na+_symporter,_DASS_family | K03319 | AxSpA | 2.465 | 0.0773 |
| gluC;_glutamate_transport_system_permease_protein | K10006 | AxSpA | 2.290 | 0.0806 |
| gluD;_glutamate_transport_system_permease_protein | K10007 | AxSpA | 2.290 | 0.0806 |
| gluA;_glutamate_transport_system_ATP-binding_protein_[EC:7.4.2.1] | K10008 | AxSpA | 2.290 | 0.0806 |
| higB;_mRNA_interferase_HigB_[EC:3.1.-.-] | K19166 | AxSpA | 2.467 | 0.0807 |
| mdh;_malate_dehydrogenase_[EC:1.1.1.37] | K00024 | AxSpA | 3.198 | 0.0807 |
| DPM1;_dolichol-phosphate_mannosyltransferase_[EC:2.4.1.83] | K00721 | AxSpA | 3.164 | 0.0807 |
| glxK,_garK;_glycerate_2-kinase_[EC:2.7.1.165] | K00865 | AxSpA | 3.328 | 0.0807 |
| thiD;_hydroxymethylpyrimidine/phosphomethylpyrimidine_kinase_[EC:2.7.1.49_2.7.4.7] | K00941 | AxSpA | 3.390 | 0.0807 |
| ispD;_2-C-methyl-D-erythritol_4-phosphate_cytidylyltransferase_[EC:2.7.7.60] | K00991 | AxSpA | 3.557 | 0.0807 |
| pulA;_pullulanase_[EC:3.2.1.41] | K01200 | AxSpA | 3.067 | 0.0807 |
| E3.5.5.1;_nitrilase_[EC:3.5.5.1] | K01501 | AxSpA | 2.561 | 0.0807 |
| menB;_naphthoate_synthase_[EC:4.1.3.36] | K01661 | AxSpA | 2.916 | 0.0807 |
| pabB;_para-aminobenzoate_synthetase_component_I_[EC:2.6.1.85] | K01665 | AxSpA | 3.047 | 0.0807 |
| ENO,_eno;_enolase_[EC:4.2.1.11] | K01689 | AxSpA | 3.532 | 0.0807 |
| ispF;_2-C-methyl-D-erythritol_2,4-cyclodiphosphate_synthase_[EC:4.6.1.12] | K01770 | AxSpA | 3.384 | 0.0807 |
| hemL;_glutamate-1-semialdehyde_2,1-aminomutase_[EC:5.4.3.8] | K01845 | AxSpA | 2.953 | 0.0807 |
| kdtA,_waaA;_3-deoxy-D-manno-octulosonic-acid_transferase_[EC:2.4.99.12_2.4.99.13_2.4.99.14_2.4.99.15] | K02527 | AxSpA | 3.002 | 0.0807 |
| mrp,_NUBPL;_ATP-binding_protein_involved_in_chromosome_partitioning | K03593 | AxSpA | 3.004 | 0.0807 |
| purR;_LacI_family_transcriptional_regulator,_purine_nucleotide_synthesis_repressor | K03604 | AxSpA | 2.965 | 0.0807 |
| apbE;_FAD:protein_FMN_transferase_[EC:2.7.1.180] | K03734 | AxSpA | 3.486 | 0.0807 |
| K06950;_uncharacterized_protein | K06950 | AxSpA | 3.471 | 0.0807 |
| yaeR;_glyoxylase_I_family_protein | K08234 | AxSpA | 2.903 | 0.0807 |
| purT;_phosphoribosylglycinamide_formyltransferase_2_[EC:2.1.2.2] | K08289 | AxSpA | 2.985 | 0.0807 |
| PANK1_2_3,_CAB1,_coaW;_type_II_pantothenate_kinase_[EC:2.7.1.33] | K09680 | AxSpA | 2.872 | 0.0807 |
| bgaB,_lacA;_beta-galactosidase_[EC:3.2.1.23] | K12308 | AxSpA | 3.427 | 0.0807 |
| lldE;_L-lactate_dehydrogenase_complex_protein_LldE | K18928 | AxSpA | 2.803 | 0.0807 |
| lldF;_L-lactate_dehydrogenase_complex_protein_LldF | K18929 | AxSpA | 2.803 | 0.0807 |
| dctP;_C4-dicarboxylate-binding_protein_DctP | K11688 | AxSpA | 2.687 | 0.0877 |
| ina;_immune_inhibitor_A_[EC:3.4.24.-] | K09607 | AxSpA | 2.630 | 0.0877 |
| virB10,_lvhB10;_type_IV_secretion_system_protein_VirB10 | K03195 | AxSpA | 2.264 | 0.0878 |
| virB11,_lvhB11;_type_IV_secretion_system_protein_VirB11_[EC:7.4.2.8] | K03196 | AxSpA | 2.264 | 0.0878 |
| virB4,_lvhB4;_type_IV_secretion_system_protein_VirB4_[EC:7.4.2.8] | K03199 | AxSpA | 2.264 | 0.0878 |
| virB5,_lvhB5;_type_IV_secretion_system_protein_VirB5 | K03200 | AxSpA | 2.264 | 0.0878 |
| IMPDH,_guaB;_IMP_dehydrogenase_[EC:1.1.1.205] | K00088 | AxSpA | 3.456 | 0.0878 |
| asrC;_anaerobic_sulfite_reductase_subunit_C | K00385 | AxSpA | 2.244 | 0.0878 |
| E2.3.1.8,_pta;_phosphate_acetyltransferase_[EC:2.3.1.8] | K00625 | AxSpA | 3.349 | 0.0878 |
| fabH;_3-oxoacyl-[acyl-carrier-protein]_synthase_III_[EC:2.3.1.180] | K00648 | AxSpA | 3.488 | 0.0878 |
| lpxK;_tetraacyldisaccharide_4'-kinase_[EC:2.7.1.130] | K00912 | AxSpA | 3.014 | 0.0878 |
| lysC;_aspartate_kinase_[EC:2.7.2.4] | K00928 | AxSpA | 3.570 | 0.0878 |
| purU;_formyltetrahydrofolate_deformylase_[EC:3.5.1.10] | K01433 | AxSpA | 2.823 | 0.0878 |
| folB;_7,8-dihydroneopterin_aldolase/epimerase/oxygenase_[EC:4.1.2.25_5.1.99.8_1.13.11.81] | K01633 | AxSpA | 3.026 | 0.0878 |
| PGAM,_gpmA;_2,3-bisphosphoglycerate-dependent_phosphoglycerate_mutase_[EC:5.4.2.11] | K01834 | AxSpA | 3.062 | 0.0878 |
| pgm;_phosphoglucomutase_[EC:5.4.2.2] | K01835 | AxSpA | 3.489 | 0.0878 |
| ACSS1_2,_acs;_acetyl-CoA_synthetase_[EC:6.2.1.1] | K01895 | AxSpA | 3.068 | 0.0878 |
| ACSL,_fadD;_long-chain_acyl-CoA_synthetase_[EC:6.2.1.3] | K01897 | AxSpA | 3.622 | 0.0878 |
| wza,_gfcE;_polysaccharide_biosynthesis/export_protein | K01991 | AxSpA | 3.254 | 0.0878 |
| ABC.CD.TX;_HlyD_family_secretion_protein | K02005 | AxSpA | 3.502 | 0.0878 |
| tuf,_TUFM;_elongation_factor_Tu | K02358 | AxSpA | 3.472 | 0.0878 |
| glgX;_glycogen_debranching_enzyme_[EC:3.2.1.196] | K02438 | AxSpA | 3.283 | 0.0878 |
| nagB,_GNPDA;_glucosamine-6-phosphate_deaminase_[EC:3.5.99.6] | K02564 | AxSpA | 3.616 | 0.0878 |
| parC;_topoisomerase_IV_subunit_A_[EC:5.6.2.2] | K02621 | AxSpA | 3.002 | 0.0878 |
| parE;_topoisomerase_IV_subunit_B_[EC:5.6.2.2] | K02622 | AxSpA | 3.024 | 0.0878 |
| RP-L30,_MRPL30,_rpmD;_large_subunit_ribosomal_protein_L30 | K02907 | AxSpA | 3.421 | 0.0878 |
| RP-L5,_MRPL5,_rplE;_large_subunit_ribosomal_protein_L5 | K02931 | AxSpA | 3.407 | 0.0878 |
| secA;_preprotein_translocase_subunit_SecA_[EC:7.4.2.8] | K03070 | AxSpA | 3.426 | 0.0878 |
| ubiE;_demethylmenaquinone_methyltransferase_/_2-methoxy-6-polyprenyl-1,4-benzoquinol_methylase_[EC:2.1.1.163_2.1.1.201] | K03183 | AxSpA | 3.029 | 0.0878 |
| fruA;_fructan_beta-fructosidase_[EC:3.2.1.80] | K03332 | AxSpA | 3.056 | 0.0878 |
| E3.6.1.22,_NUDT12,_nudC;_NAD+_diphosphatase_[EC:3.6.1.22] | K03426 | AxSpA | 3.336 | 0.0878 |
| exbB;_biopolymer_transport_protein_ExbB | K03561 | AxSpA | 3.400 | 0.0878 |
| moeA;_molybdopterin_molybdotransferase_[EC:2.10.1.1] | K03750 | AxSpA | 2.803 | 0.0878 |
| ppiD;_peptidyl-prolyl_cis-trans_isomerase_D_[EC:5.2.1.8] | K03770 | AxSpA | 3.028 | 0.0878 |
| surE;_5'-nucleotidase_[EC:3.1.3.5] | K03787 | AxSpA | 3.041 | 0.0878 |
| mtgA;_monofunctional_glycosyltransferase_[EC:2.4.1.129] | K03814 | AxSpA | 3.005 | 0.0878 |
| dedA;_membrane-associated_protein | K03975 | AxSpA | 2.869 | 0.0878 |
| dsbC;_thiol:disulfide_interchange_protein_DsbC_[EC:5.3.4.1] | K03981 | AxSpA | 2.568 | 0.0878 |
| rarD;_chloramphenicol-sensitive_protein_RarD | K05786 | AxSpA | 2.640 | 0.0878 |
| phnP;_phosphoribosyl_1,2-cyclic_phosphate_phosphodiesterase_[EC:3.1.4.55] | K06167 | AxSpA | 2.998 | 0.0878 |
| K07139;_uncharacterized_protein | K07139 | AxSpA | 3.395 | 0.0878 |
| kptA;_putative_RNA_2'-phosphotransferase_[EC:2.7.1.-] | K07559 | AxSpA | 2.426 | 0.0878 |
| dcuA;_anaerobic_C4-dicarboxylate_transporter_DcuA | K07791 | AxSpA | 3.005 | 0.0878 |
| K08999;_uncharacterized_protein | K08999 | AxSpA | 2.914 | 0.0878 |
| K09768;_uncharacterized_protein | K09768 | AxSpA | 3.006 | 0.0878 |
| K09955;_uncharacterized_protein | K09955 | AxSpA | 3.519 | 0.0878 |
| asrA;_anaerobic_sulfite_reductase_subunit_A | K16950 | AxSpA | 2.244 | 0.0878 |
| asrB;_anaerobic_sulfite_reductase_subunit_B | K16951 | AxSpA | 2.244 | 0.0878 |
| casC,_cse4;_CRISPR_system_Cascade_subunit_CasC | K19124 | AxSpA | 2.332 | 0.0878 |
| senX3;_two-component_system,_OmpR_family,_sensor_histidine_kinase_SenX3_[EC:2.7.13.3] | K07768 | AxSpA | 2.399 | 0.0947 |
| glgM;_alpha-maltose-1-phosphate_synthase_[EC:2.4.1.342] | K16148 | AxSpA | 2.399 | 0.0947 |
|  | K02763 | AxSpA | 2.552 | 0.0951 |
| iorB;_indolepyruvate_ferredoxin_oxidoreductase,_beta_subunit_[EC:1.2.7.8] | K00180 | AxSpA | 3.139 | 0.0955 |
| sdhB,_frdB;_succinate_dehydrogenase_/_fumarate_reductase,_iron-sulfur_subunit_[EC:1.3.5.1_1.3.5.4] | K00240 | AxSpA | 3.036 | 0.0955 |
| sdhC,_frdC;_succinate_dehydrogenase_/_fumarate_reductase,_cytochrome_b_subunit | K00241 | AxSpA | 2.975 | 0.0955 |
| E2.2.1.1,_tktA,_tktB;_transketolase_[EC:2.2.1.1] | K00615 | AxSpA | 3.870 | 0.0955 |
| lpxA;_UDP-N-acetylglucosamine_acyltransferase_[EC:2.3.1.129] | K00677 | AxSpA | 3.272 | 0.0955 |
| lpxB;_lipid-A-disaccharide_synthase_[EC:2.4.1.182] | K00748 | AxSpA | 3.025 | 0.0955 |
| trpD;_anthranilate_phosphoribosyltransferase_[EC:2.4.2.18] | K00766 | AxSpA | 3.330 | 0.0955 |
| miaA,_TRIT1;_tRNA_dimethylallyltransferase_[EC:2.5.1.75] | K00791 | AxSpA | 3.522 | 0.0955 |
| glmS,_GFPT;_glutamine---fructose-6-phosphate_transaminase_(isomerizing)_[EC:2.6.1.16] | K00820 | AxSpA | 3.473 | 0.0955 |
| E2.7.1.4,_scrK;_fructokinase_[EC:2.7.1.4] | K00847 | AxSpA | 3.592 | 0.0955 |
| folK;_2-amino-4-hydroxy-6-hydroxymethyldihydropteridine_diphosphokinase_[EC:2.7.6.3] | K00950 | AxSpA | 3.270 | 0.0955 |
| pldB;_lysophospholipase_[EC:3.1.1.5] | K01048 | AxSpA | 2.806 | 0.0955 |
| E3.2.1.89;_arabinogalactan_endo-1,4-beta-galactosidase_[EC:3.2.1.89] | K01224 | AxSpA | 3.198 | 0.0955 |
| pepP;_Xaa-Pro_aminopeptidase_[EC:3.4.11.9] | K01262 | AxSpA | 3.551 | 0.0955 |
| kdsA;_2-dehydro-3-deoxyphosphooctonate_aldolase_(KDO_8-P_synthase)_[EC:2.5.1.55] | K01627 | AxSpA | 3.027 | 0.0955 |
| TPI,_tpiA;_triosephosphate_isomerase_(TIM)_[EC:5.3.1.1] | K01803 | AxSpA | 3.489 | 0.0955 |
| MARS,_metG;_methionyl-tRNA_synthetase_[EC:6.1.1.10] | K01874 | AxSpA | 3.444 | 0.0955 |
| SARS,_serS;_seryl-tRNA_synthetase_[EC:6.1.1.11] | K01875 | AxSpA | 3.431 | 0.0955 |
| FARSB,_pheT;_phenylalanyl-tRNA_synthetase_beta_chain_[EC:6.1.1.20] | K01890 | AxSpA | 3.435 | 0.0955 |
| NARS,_asnS;_asparaginyl-tRNA_synthetase_[EC:6.1.1.22] | K01893 | AxSpA | 3.384 | 0.0955 |
| menE;_o-succinylbenzoate---CoA_ligase_[EC:6.2.1.26] | K01911 | AxSpA | 2.888 | 0.0955 |
| mlaF,_linL,_mkl;_phospholipid/cholesterol/gamma-HCH_transport_system_ATP-binding_protein | K02065 | AxSpA | 3.038 | 0.0955 |
| mlaE,_linK;_phospholipid/cholesterol/gamma-HCH_transport_system_permease_protein | K02066 | AxSpA | 3.038 | 0.0955 |
| holB;_DNA_polymerase_III_subunit_delta'_[EC:2.7.7.7] | K02341 | AxSpA | 3.414 | 0.0955 |
| flgJ;_peptidoglycan_hydrolase_FlgJ | K02395 | AxSpA | 2.798 | 0.0955 |
| ispB;_octaprenyl-diphosphate_synthase_[EC:2.5.1.90] | K02523 | AxSpA | 3.001 | 0.0955 |
| pilA;_type_IV_pilus_assembly_protein_PilA | K02650 | AxSpA | 2.907 | 0.0955 |
| pyrR;_pyrimidine_operon_attenuation_protein_/_uracil_phosphoribosyltransferase_[EC:2.4.2.9] | K02825 | AxSpA | 2.548 | 0.0955 |
| waaC,_rfaC;_heptosyltransferase_I_[EC:2.4.-.-] | K02841 | AxSpA | 2.615 | 0.0955 |
| lepB;_signal_peptidase_I_[EC:3.4.21.89] | K03100 | AxSpA | 3.826 | 0.0955 |
| kdsC;_3-deoxy-D-manno-octulosonate_8-phosphate_phosphatase_(KDO_8-P_phosphatase)_[EC:3.1.3.45] | K03270 | AxSpA | 3.106 | 0.0955 |
| gmhB;_D-glycero-D-manno-heptose_1,7-bisphosphate_phosphatase_[EC:3.1.3.82_3.1.3.83] | K03273 | AxSpA | 2.832 | 0.0955 |
| mraW,_rsmH;_16S_rRNA_(cytosine1402-N4)-methyltransferase_[EC:2.1.1.199] | K03438 | AxSpA | 3.540 | 0.0955 |
| gidA,_mnmG,_MTO1;_tRNA_uridine_5-carboxymethylaminomethyl_modification_enzyme | K03495 | AxSpA | 3.413 | 0.0955 |
| E3.4.21.102,_prc,_ctpA;_carboxyl-terminal_processing_protease_[EC:3.4.21.102] | K03797 | AxSpA | 3.610 | 0.0955 |
| mraZ;_MraZ_protein | K03925 | AxSpA | 3.434 | 0.0955 |
| pnbA;_para-nitrobenzyl_esterase_[EC:3.1.1.-] | K03929 | AxSpA | 2.606 | 0.0955 |
| nrdG;_anaerobic_ribonucleoside-triphosphate_reductase_activating_protein_[EC:1.97.1.4] | K04068 | AxSpA | 3.468 | 0.0955 |
| groEL,_HSPD1;_chaperonin_GroEL | K04077 | AxSpA | 3.424 | 0.0955 |
| iscU,_nifU;_nitrogen_fixation_protein_NifU_and_related_proteins | K04488 | AxSpA | 3.381 | 0.0955 |
| xerD;_integrase/recombinase_XerD | K04763 | AxSpA | 3.435 | 0.0955 |
| kdsD,_kpsF;_arabinose-5-phosphate_isomerase_[EC:5.3.1.13] | K06041 | AxSpA | 3.025 | 0.0955 |
| lptB;_lipopolysaccharide_export_system_ATP-binding_protein_[EC:3.6.3.-] | K06861 | AxSpA | 3.025 | 0.0955 |
| pgpH;_cyclic-di-AMP_phosphodiesterase_PgpH_[EC:3.1.4.-] | K07037 | AxSpA | 3.040 | 0.0955 |
| K07052;_uncharacterized_protein | K07052 | AxSpA | 3.666 | 0.0955 |
| licD;_lipopolysaccharide_cholinephosphotransferase_[EC:2.7.8.-] | K07271 | AxSpA | 3.355 | 0.0955 |
| pepD;_putative_serine_protease_PepD_[EC:3.4.21.-] | K08372 | AxSpA | 2.256 | 0.0955 |
| GAE,_cap1J;_UDP-glucuronate_4-epimerase_[EC:5.1.3.6] | K08679 | AxSpA | 2.471 | 0.0955 |
| ybiA;_N-glycosidase_YbiA_[EC:3.2.2.-] | K09935 | AxSpA | 2.570 | 0.0955 |
| lptG;_lipopolysaccharide_export_system_permease_protein | K11720 | AxSpA | 3.025 | 0.0955 |
| npdA;_NAD-dependent_deacetylase_[EC:2.3.1.286] | K12410 | AxSpA | 3.451 | 0.0955 |
| hspR;_MerR_family_transcriptional_regulator,_heat_shock_protein_HspR | K13640 | AxSpA | 2.256 | 0.0955 |
| idsA;_geranylgeranyl_diphosphate_synthase,_type_I_[EC:2.5.1.1_2.5.1.10_2.5.1.29] | K13787 | AxSpA | 2.256 | 0.0955 |
| yfiC,_trmX;_tRNA1Val_(adenine37-N6)-methyltransferase_[EC:2.1.1.223] | K15460 | AxSpA | 3.218 | 0.0955 |

| **Feces_AxSpA vs HC_IgA-_a0.1_w0.05_l2_minc.default.res.sig.txt** | | | | |
| --- | --- | --- | --- | --- |
| **Level D (KO) _Description** | **KO** | **Class with highest mean** | **Log LDA score** | **p-value (KW for class)** |
| gldA;_glycerol_dehydrogenase_[EC:1.1.1.6] | K00005 | HC | 2.975 | 0.0955 |
| algD;_GDP-mannose_6-dehydrogenase_[EC:1.1.1.132] | K00066 | HC | 2.256 | 0.0801 |
| E1.1.1.219;_dihydroflavonol-4-reductase_[EC:1.1.1.219] | K00091 | HC | 2.127 | 0.0807 |
| hpaE,_hpcC;_5-carboxymethyl-2-hydroxymuconic-semialdehyde_dehydrogenase_[EC:1.2.1.60] | K00151 | HC | 2.185 | 0.0607 |
| dmsB;_dimethyl_sulfoxide_reductase_iron-sulfur_subunit | K00184 | HC | 2.587 | 0.0995 |
| DHODH,_pyrD;_dihydroorotate_dehydrogenase_[EC:1.3.5.2] | K00254 | HC | 2.935 | 0.0878 |
| gcvPA;_glycine_dehydrogenase_subunit_1_[EC:1.4.4.2] | K00282 | HC | 2.594 | 0.0213 |
| gcvPB;_glycine_dehydrogenase_subunit_2_[EC:1.4.4.2] | K00283 | HC | 2.587 | 0.0390 |
| E1.11.1.5;_cytochrome_c_peroxidase_[EC:1.11.1.5] | K00428 | HC | 2.946 | 0.0567 |
| ermC,_ermA;_23S_rRNA_(adenine-N6)-dimethyltransferase_[EC:2.1.1.184] | K00561 | HC | 2.945 | 0.0567 |
| panB;_3-methyl-2-oxobutanoate_hydroxymethyltransferase_[EC:2.1.2.11] | K00606 | HC | 3.449 | 0.0741 |
| aacC;_aminoglycoside_3-N-acetyltransferase_[EC:2.3.1.81] | K00662 | HC | 2.184 | 0.0878 |
| lldG;_L-lactate_dehydrogenase_complex_protein_LldG | K00782 | HC | 3.106 | 0.0878 |
| pct;_propionate_CoA-transferase_[EC:2.8.3.1] | K01026 | HC | 2.682 | 0.0354 |
| atoD;_acetate_CoA/acetoacetate_CoA-transferase_alpha_subunit_[EC:2.8.3.8_2.8.3.9] | K01034 | HC | 2.647 | 0.0518 |
| atoA;_acetate_CoA/acetoacetate_CoA-transferase_beta_subunit_[EC:2.8.3.8_2.8.3.9] | K01035 | HC | 2.649 | 0.0390 |
| gctA;_glutaconate_CoA-transferase,_subunit_A_[EC:2.8.3.12] | K01039 | HC | 2.323 | 0.0192 |
| gctB;_glutaconate_CoA-transferase,_subunit_B_[EC:2.8.3.12] | K01040 | HC | 2.323 | 0.0192 |
| NEU1;_sialidase-1_[EC:3.2.1.18] | K01186 | HC | 3.134 | 0.0429 |
| E3.2.1.86A,_celF;_6-phospho-beta-glucosidase_[EC:3.2.1.86] | K01222 | HC | 2.685 | 0.0679 |
| E3.5.1.4,_amiE;_amidase_[EC:3.5.1.4] | K01426 | HC | 2.632 | 0.0429 |
| arcA;_arginine_deiminase_[EC:3.5.3.6] | K01478 | HC | 2.453 | 0.0807 |
| dcd;_dCTP_deaminase_[EC:3.5.4.13] | K01494 | HC | 2.421 | 0.0955 |
| hisI;_phosphoribosyl-AMP_cyclohydrolase_[EC:3.5.4.19] | K01496 | HC | 2.858 | 0.0567 |
| kdpB;_potassium-trAxSpArting_ATPase_ATP-binding_subunit_[EC:7.2.2.6] | K01547 | HC | 2.965 | 0.0807 |
| E4.1.1.32,_pckA,_PCK;_phosphoenolpyruvate_carboxykinase_(GTP)_[EC:4.1.1.32] | K01596 | HC | 2.837 | 0.0518 |
| FBA,_fbaA;_fructose-bisphosphate_aldolase,_class_II_[EC:4.1.2.13] | K01624 | HC | 3.687 | 0.0955 |
| PPCS,_COAB;_phosphopantothenate---cysteine_ligase_(ATP)_[EC:6.3.2.51] | K01922 | HC | 2.472 | 0.0290 |
| PC,_pyc;_pyruvate_carboxylase_[EC:6.4.1.1] | K01958 | HC | 2.684 | 0.0290 |
| phnE;_phosphonate_trAxSpArt_system_permease_protein | K02042 | HC | 2.411 | 0.0878 |
| comFB;_competence_protein_ComFB | K02241 | HC | 2.407 | 0.0679 |
| cpaB,_rcpC;_pilus_assembly_protein_CpaB | K02279 | HC | 2.247 | 0.0955 |
| flbD;_flagellar_protein_FlbD | K02385 | HC | 2.932 | 0.0741 |
| paaA;_ring-1,2-phenylacetyl-CoA_epoxidase_subunit_PaaA_[EC:1.14.13.149] | K02609 | HC | 2.151 | 0.0607 |
| paaB;_ring-1,2-phenylacetyl-CoA_epoxidase_subunit_PaaB | K02610 | HC | 2.197 | 0.0607 |
| paaC;_ring-1,2-phenylacetyl-CoA_epoxidase_subunit_PaaC_[EC:1.14.13.149] | K02611 | HC | 2.195 | 0.0607 |
| paaD;_ring-1,2-phenylacetyl-CoA_epoxidase_subunit_PaaD | K02612 | HC | 2.229 | 0.0607 |
| paaE;_ring-1,2-phenylacetyl-CoA_epoxidase_subunit_PaaE | K02613 | HC | 2.269 | 0.0607 |
| paaZ;_oxepin-CoA_hydrolase_/_3-oxo-5,6-dehydrosuberyl-CoA_semialdehyde_dehydrogenase_[EC:3.3.2.12_1.2.1.91] | K02618 | HC | 2.159 | 0.0607 |
| celB,_chbC;_cellobiose_PTS_system_EIIC_component | K02761 | HC | 2.993 | 0.0807 |
| secD;_preprotein_translocase_subunit_SecD | K03072 | HC | 3.014 | 0.0955 |
| secF;_preprotein_translocase_subunit_SecF | K03074 | HC | 3.044 | 0.0807 |
| sigB;_RNA_polymerase_sigma-B_factor | K03090 | HC | 2.682 | 0.0741 |
| corA;_magnesium_trAxSpArter | K03284 | HC | 3.664 | 0.0955 |
| emrE,_qac,_mmr,_smr;_small_multidrug_resistance_pump | K03297 | HC | 2.358 | 0.0804 |
| iolC;_5-dehydro-2-deoxygluconokinase_[EC:2.7.1.92] | K03338 | HC | 2.493 | 0.0429 |
| cheX;_chemotaxis_protein_CheX | K03409 | HC | 2.542 | 0.0807 |
| ugtP;_processive_1,2-diacylglycerol_beta-glucosyltransferase_[EC:2.4.1.315] | K03429 | HC | 3.004 | 0.0390 |
| ERD6,_ESL1;_MFS_trAxSpArter,_SP_family,_ERD6-like_sugar_trAxSpArter | K03444 | HC | 2.887 | 0.0955 |
| chbR,_celD;_AraC_family_transcriptional_regulator,_dual_regulator_of_chb_operon | K03490 | HC | 2.245 | 0.0621 |
| ydhQ;_GntR_family_transcriptional_regulator | K03492 | HC | 2.232 | 0.0429 |
| MPG;_DNA-3-methyladenine_glycosylase_[EC:3.2.2.21] | K03652 | HC | 2.363 | 0.0807 |
| helD;_DNA_helicase_IV_[EC:3.6.4.12] | K03658 | HC | 2.416 | 0.0955 |
| ctsR;_transcriptional_regulator_of_stress_and_heat_shock_response | K03708 | HC | 2.938 | 0.0290 |
| lplA,_lplJ,_lipL1;_lipoate---protein_ligase_[EC:6.3.1.20] | K03800 | HC | 3.021 | 0.0741 |
| mnhB,_mrpB;_multicomponent_Na+:H+_antiporter_subunit_B | K05566 | HC | 2.118 | 0.0518 |
| mnhC,_mrpC;_multicomponent_Na+:H+_antiporter_subunit_C | K05567 | HC | 2.110 | 0.0678 |
| mnhD,_mrpD;_multicomponent_Na+:H+_antiporter_subunit_D | K05568 | HC | 2.556 | 0.0567 |
| mnhE,_mrpE;_multicomponent_Na+:H+_antiporter_subunit_E | K05569 | HC | 2.110 | 0.0621 |
| mnhF,_mrpF;_multicomponent_Na+:H+_antiporter_subunit_F | K05570 | HC | 2.110 | 0.0678 |
| mnhG,_mrpG;_multicomponent_Na+:H+_antiporter_subunit_G | K05571 | HC | 2.110 | 0.0678 |
| pdhR;_GntR_family_transcriptional_regulator,_transcriptional_repressor_for_pyruvate_dehydrogenase_complex | K05799 | HC | 3.063 | 0.0878 |
| dapH,_dapD;_tetrahydrodipicolinate_N-acetyltransferase_[EC:2.3.1.89] | K05822 | HC | 2.039 | 0.0807 |
| ppaX;_pyrophosphatase_PpaX_[EC:3.6.1.1] | K06019 | HC | 2.641 | 0.0390 |
| dkgA;_2,5-diketo-D-gluconate_reductase_A_[EC:1.1.1.346] | K06221 | HC | 2.424 | 0.0878 |
| gerKB;_spore_germination_protein_KB | K06296 | HC | 2.485 | 0.0038 |
| gerKC;_spore_germination_protein_KC | K06297 | HC | 2.519 | 0.0099 |
| spoIVFB;_stage_IV_sporulation_protein_FB_[EC:3.4.24.-] | K06402 | HC | 2.760 | 0.0472 |
| spoVS;_stage_V_sporulation_protein_S | K06416 | HC | 2.900 | 0.0679 |
| sspD;_small_acid-soluble_spore_protein_D_(minor_alpha/beta-type_SASP) | K06421 | HC | 2.855 | 0.0446 |
| K06888;_uncharacterized_protein | K06888 | HC | 2.426 | 0.0472 |
| K06973;_uncharacterized_protein | K06973 | HC | 3.547 | 0.0807 |
| K06976;_uncharacterized_protein | K06976 | HC | 2.111 | 0.0649 |
| cas3;_CRISPR-associated_endonuclease/helicase_Cas3_[EC:3.1.-.-_3.6.4.-] | K07012 | HC | 3.442 | 0.0878 |
| K07013;_uncharacterized_protein | K07013 | HC | 2.220 | 0.0867 |
| K07071;_uncharacterized_protein | K07071 | HC | 2.690 | 0.0741 |
| kynB;_arylformamidase_[EC:3.5.1.9] | K07130 | HC | 2.566 | 0.0354 |
| K07222;_putative_flavoprotein_involved_in_K+_trAxSpArt | K07222 | HC | 2.171 | 0.0607 |
| efeO;_iron_uptake_system_component_EfeO | K07224 | HC | 2.166 | 0.0676 |
| ybaZ;_methylated-DNA-protein-cysteine_methyltransferase_related_protein | K07443 | HC | 3.083 | 0.0807 |
| cas4;_CRISPR-associated_exonuclease_Cas4_[EC:3.1.12.1] | K07464 | HC | 3.335 | 0.0807 |
| mgtC;_putative_Mg2+_trAxSpArter-C_(MgtC)_family_protein | K07507 | HC | 3.390 | 0.0878 |
| ygaC;_uncharacterized_protein | K07586 | HC | 2.538 | 0.0679 |
| smvA,_qacA,_lfrA;_MFS_trAxSpArter,_DHA2_family,_multidrug_resistance_protein | K08167 | HC | 2.150 | 0.0607 |
| CRLS;_cardiolipin_synthase_(CMP-forming)_[EC:2.7.8.41] | K08744 | HC | 2.988 | 0.0878 |
| mtnA;_methylthioribose-1-phosphate_isomerase_[EC:5.3.1.23] | K08963 | HC | 2.982 | 0.0955 |
| patA;_putrescine_aminotransferase_[EC:2.6.1.82] | K09251 | HC | 2.441 | 0.0741 |
| K09704;_uncharacterized_protein | K09704 | HC | 3.226 | 0.0679 |
| K09797;_uncharacterized_protein | K09797 | HC | 3.126 | 0.0807 |
| K09925;_uncharacterized_protein | K09925 | HC | 2.147 | 0.0492 |
| cas2;_CRISPR-associated_protein_Cas2 | K09951 | HC | 3.496 | 0.0955 |
| cynR;_LysR_family_transcriptional_regulator,_cyn_operon_transcriptional_activator | K11921 | HC | 2.845 | 0.0445 |
| msrAB;_peptide_methionine_sulfoxide_reductase_msrA/msrB_[EC:1.8.4.11_1.8.4.12] | K12267 | HC | 3.351 | 0.0679 |
| HEXA_B;_hexosaminidase_[EC:3.2.1.52] | K12373 | HC | 3.868 | 0.0741 |
| wbpA;_UDP-N-acetyl-D-glucosamine_dehydrogenase_[EC:1.1.1.136] | K13015 | HC | 2.086 | 0.0878 |
| wbpD,_wlbB;_UDP-2-acetamido-3-amino-2,3-dideoxy-glucuronate_N-acetyltransferase_[EC:2.3.1.201] | K13018 | AxSpA | 2.283 | 0.0679 |
| wbpI,_wlbD;_UDP-GlcNAc3NAcA_epimerase_[EC:5.1.3.23] | K13019 | HC | 2.366 | 0.0192 |
| trpGD;_anthranilate_synthase/phosphoribosyltransferase_[EC:4.1.3.27_2.4.2.18] | K13497 | HC | 2.480 | 0.0741 |
| pksJ;_polyketide_synthase_PksJ | K13611 | HC | 2.335 | 0.0365 |
| cheBR;_two-component_system,_chemotaxis_family,_CheB/CheR_fusion_protein_[EC:2.1.1.80_3.1.1.61] | K13924 | HC | 2.137 | 0.0770 |
| yahK;_alcohol_dehydrogenase_(NADP+)_[EC:1.1.1.2] | K13979 | HC | 2.334 | 0.0213 |
| actP;_cation/acetate_symporter | K14393 | HC | 2.137 | 0.0607 |
| ICP;_inhibitor_of_cysteine_peptidase | K14475 | HC | 2.201 | 0.0618 |
| aldH;_NADP-dependent_aldehyde_dehydrogenase_[EC:1.2.1.4] | K14519 | HC | 2.162 | 0.0607 |
| abfD;_4-hydroxybutyryl-CoA_dehydratase_/_vinylacetyl-CoA-Delta-isomerase_[EC:4.2.1.120_5.3.3.3] | K14534 | HC | 2.584 | 0.0354 |
| ylbA,_UGHY;_(S)-ureidoglycine_aminohydrolase_[EC:3.5.3.26] | K14977 | HC | 2.220 | 0.0867 |
| cbe,_mbe;_cellobiose_epimerase_[EC:5.1.3.11] | K16213 | HC | 3.138 | 0.0518 |
| efeB;_deferrochelatase/peroxidase_EfeB_[EC:1.11.1.-] | K16301 | HC | 2.246 | 0.0515 |
| tcyL;_L-cystine_trAxSpArt_system_permease_protein | K16958 | AxSpA | 2.453 | 0.0285 |
| lysX1;_putative_lysine_trAxSpArt_system_substrate-binding_protein | K17073 | HC | 2.591 | 0.0955 |
| lysX2;_putative_lysine_trAxSpArt_system_permease_protein | K17074 | HC | 2.583 | 0.0518 |
| K17076,_lysY;_putative_lysine_trAxSpArt_system_ATP-binding_protein_[EC:3.6.3.-] | K17076 | HC | 2.613 | 0.0518 |
| SUPV3L1,_SUV3;_ATP-dependent_RNA_helicase_SUPV3L1/SUV3_[EC:3.6.4.13] | K17675 | HC | 2.140 | 0.0390 |
| wbiB;_dTDP-L-rhamnose_4-epimerase_[EC:5.1.3.25] | K17947 | HC | 2.241 | 0.0589 |
| vanT;_serine/alanine_racemase_[EC:5.1.1.18_5.1.1.1] | K18348 | HC | 2.161 | 0.0955 |
| mp2;_beta-1,4-mannooligosaccharide/beta-1,4-mannosyl-N-acetylglucosamine_phosphorylase_[EC:2.4.1.319_2.4.1.320] | K18785 | HC | 3.160 | 0.0518 |
| vanC,_vanE,_vanG;_D-alanine---D-serine_ligase_[EC:6.3.2.35] | K18856 | HC | 2.570 | 0.0807 |
| prdX,_proX;_Ala-tRNA(Pro)_deacylase_[EC:3.1.1.-] | K19055 | HC | 3.307 | 0.0878 |
| epsD;_glycosyltransferase_EpsD_[EC:2.4.-.-] | K19422 | HC | 2.639 | 0.0621 |
| epsJ;_glycosyltransferase_EpsJ_[EC:2.4.-.-] | K19427 | HC | 2.208 | 0.0552 |

| **Saliva_AxSpA vs HC_IgA+_a0.1_w0.05_l2_minc.default.res.sig.txt** | | | | |
| --- | --- | --- | --- | --- |
| **Level D (KO) _Description** | **KO** | **Class with highest mean** | **Log LDA score** | **p-value (KW for class)** |
| K09005;_uncharacterized_protein | K09005 | HC | 2.673 | 0.0007 |
| pel;_pectate_lyase_[EC:4.2.2.2] | K01728 | HC | 2.632 | 0.0020 |
| E4.1.3.4,_HMGCL,_hmgL;_hydroxymethylglutaryl-CoA_lyase_[EC:4.1.3.4] | K01640 | HC | 2.634 | 0.0022 |
| wapR;_alpha-1,3-rhamnosyltransferase_[EC:2.4.1.-] | K12988 | HC | 2.433 | 0.0023 |
| pksJ;_polyketide_synthase_PksJ | K13611 | HC | 2.505 | 0.0024 |
| yxeO;_putative_S-methylcysteine_transport_system_ATP-binding_protein | K16963 | HC | 2.185 | 0.0024 |
| nadM;_nicotinamide-nucleotide_adenylyltransferase_[EC:2.7.7.1] | K00952 | HC | 2.180 | 0.0028 |
| tupA,_vupA;_tungstate_transport_system_substrate-binding_protein | K05772 | HC | 2.214 | 0.0029 |
| tupB,_vupB;_tungstate_transport_system_permease_protein | K05773 | HC | 2.215 | 0.0029 |
| tupC,_vupC;_tungstate_transport_system_ATP-binding_protein_[EC:7.3.2.6] | K06857 | HC | 2.216 | 0.0029 |
| mqnC;_cyclic_dehypoxanthinyl_futalosine_synthase_[EC:1.21.98.1] | K11784 | HC | 2.234 | 0.0029 |
| mqnE;_aminodeoxyfutalosine_synthase_[EC:2.5.1.120] | K18285 | HC | 2.319 | 0.0029 |
| fabV,_ter;_enoyl-[acyl-carrier_protein]_reductase_/_trans-2-enoyl-CoA_reductase_(NAD+)_[EC:1.3.1.9_1.3.1.44] | K00209 | HC | 2.889 | 0.0034 |
| hoxN,_nixA;_nickel/cobalt_transporter_(NiCoT)_family_protein | K07241 | AxSpA | 2.287 | 0.0038 |
| ABCB1,_CD243;_ATP-binding_cassette,_subfamily_B_(MDR/TAP),_member_1_[EC:7.6.2.2] | K05658 | AxSpA | 2.215 | 0.0038 |
| lnuA_C_D_E,_lin;_lincosamide_nucleotidyltransferase_A/C/D/E | K19545 | AxSpA | 2.369 | 0.0042 |
| cobC1,_cobC;_cobalamin_biosynthesis_protein_CobC | K02225 | HC | 2.406 | 0.0047 |
| STE24;_STE24_endopeptidase_[EC:3.4.24.84] | K06013 | HC | 2.356 | 0.0047 |
| aqpZ;_aquaporin_Z | K06188 | AxSpA | 3.453 | 0.0047 |
| hoxH;_NAD-reducing_hydrogenase_large_subunit_[EC:1.12.1.2] | K00436 | HC | 2.199 | 0.0048 |
| rubB,_alkT;_rubredoxin---NAD+_reductase_[EC:1.18.1.1] | K05297 | HC | 2.588 | 0.0064 |
| aac6-I,_aacA7;_aminoglycoside_6'-N-acetyltransferase_I_[EC:2.3.1.82] | K18816 | HC | 2.896 | 0.0064 |
| ramB;_XRE_family_transcriptional_regulator,_fatty_acid_utilization_regulator | K07110 | HC | 2.139 | 0.0075 |
| algA,_xanB,_rfbA,_wbpW,_pslB;_mannose-1-phosphate_guanylyltransferase_/_mannose-6-phosphate_isomerase_[EC:2.7.7.13_5.3.1.8] | K16011 | HC | 2.415 | 0.0075 |
| cpaB,_rcpC;_pilus_assembly_protein_CpaB | K02279 | HC | 2.720 | 0.0075 |
| flgJ;_peptidoglycan_hydrolase_FlgJ | K02395 | HC | 2.868 | 0.0075 |
| mqnD;_1,4-dihydroxy-6-naphthoate_synthase_[EC:1.14.-.-] | K11785 | HC | 2.111 | 0.0075 |
| urtA;_urea_transport_system_substrate-binding_protein | K11959 | HC | 2.526 | 0.0075 |
| K16153;_glycogen_phosphorylase/synthase_[EC:2.4.1.1_2.4.1.11] | K16153 | HC | 2.306 | 0.0075 |
| pdc;_phenolic_acid_decarboxylase_[EC:4.1.1.-] | K13727 | AxSpA | 2.245 | 0.0078 |
| RYR2;_ryanodine_receptor_2 | K04962 | HC | 2.635 | 0.0085 |
| nifD;_nitrogenase_molybdenum-iron_protein_alpha_chain_[EC:1.18.6.1] | K02586 | HC | 2.098 | 0.0087 |
| terD;_tellurium_resistance_protein_TerD | K05795 | HC | 2.544 | 0.0087 |
| ttuC,_dmlA;_tartrate_dehydrogenase/decarboxylase_/_D-malate_dehydrogenase_[EC:1.1.1.93_4.1.1.73_1.1.1.83] | K07246 | HC | 2.573 | 0.0087 |
| E4.1.1.82;_phosphonopyruvate_decarboxylase_[EC:4.1.1.82] | K09459 | HC | 2.101 | 0.0087 |
| grdB;_glycine_reductase_complex_component_B_subunit_gamma_[EC:1.21.4.2] | K10672 | HC | 3.016 | 0.0087 |
| dgs,_bgsA;_1,2-diacylglycerol-3-alpha-glucose_alpha-1,2-glucosyltransferase_[EC:2.4.1.208] | K13677 | HC | 2.896 | 0.0087 |
| E2.6.1.18;_beta-alanine--pyruvate_transaminase_[EC:2.6.1.18] | K00822 | HC | 2.434 | 0.0094 |
| ATE1;_arginyl-tRNA---protein_transferase_[EC:2.3.2.8] | K00685 | HC | 2.442 | 0.0101 |
| cdh;_CDP-diacylglycerol_pyrophosphatase_[EC:3.6.1.26] | K01521 | AxSpA | 2.155 | 0.0101 |
| aroH;_chorismate_mutase_[EC:5.4.99.5] | K06208 | HC | 2.321 | 0.0101 |
| mprA;_two-component_system,_OmpR_family,_response_regulator_MprA | K07669 | HC | 2.119 | 0.0101 |
| aroKB;_shikimate_kinase_/_3-dehydroquinate_synthase_[EC:2.7.1.71_4.2.3.4] | K13829 | HC | 2.169 | 0.0101 |
| hemE,_UROD;_uroporphyrinogen_decarboxylase_[EC:4.1.1.37] | K01599 | AxSpA | 3.772 | 0.0117 |
| treS;_maltose_alpha-D-glucosyltransferase_/_alpha-amylase_[EC:5.4.99.16_3.2.1.1] | K05343 | HC | 2.906 | 0.0117 |
| K07157;_uncharacterized_protein | K07157 | HC | 2.397 | 0.0117 |
| K09925;_uncharacterized_protein | K09925 | HC | 2.269 | 0.0117 |
| assT;_arylsulfate_sulfotransferase_[EC:2.8.2.22] | K01023 | HC | 2.426 | 0.0136 |
| cpaE,_tadZ;_pilus_assembly_protein_CpaE | K02282 | HC | 2.243 | 0.0136 |
| cbiX;_sirohydrochlorin_cobaltochelatase_[EC:4.99.1.3] | K03795 | HC | 2.496 | 0.0136 |
| phbC,_phaC;_polyhydroxyalkanoate_synthase_subunit_PhaC_[EC:2.3.1.-] | K03821 | HC | 2.522 | 0.0136 |
| K07395;_putative_proteasome-type_protease | K07395 | HC | 2.501 | 0.0136 |
| CYC;_cytochrome_c | K08738 | HC | 2.644 | 0.0136 |
| K09805;_uncharacterized_protein | K09805 | HC | 2.848 | 0.0136 |
| tadC;_tight_adherence_protein_C | K12511 | HC | 2.835 | 0.0136 |
| PARK7;_protein_DJ-1_[EC:3.5.1.124] | K05687 | AxSpA | 2.527 | 0.0154 |
| pcaH;_protocatechuate_3,4-dioxygenase,_beta_subunit_[EC:1.13.11.3] | K00449 | HC | 2.154 | 0.0156 |
| HK;_hexokinase_[EC:2.7.1.1] | K00844 | HC | 2.499 | 0.0156 |
| NEU1;_sialidase-1_[EC:3.2.1.18] | K01186 | AxSpA | 3.671 | 0.0156 |
| E4.6.1.1;_adenylate_cyclase_[EC:4.6.1.1] | K01768 | HC | 2.849 | 0.0156 |
| rpiA;_ribose_5-phosphate_isomerase_A_[EC:5.3.1.6] | K01807 | AxSpA | 3.754 | 0.0156 |
| flgH;_flagellar_L-ring_protein_FlgH | K02393 | HC | 2.390 | 0.0156 |
| cheX;_chemotaxis_protein_CheX | K03409 | HC | 2.566 | 0.0156 |
| K07075;_uncharacterized_protein | K07075 | HC | 3.030 | 0.0156 |
| cdgJ;_c-di-GMP_phosphodiesterase_[EC:3.1.4.52] | K07181 | HC | 2.926 | 0.0156 |
| frlC;_fructoselysine_3-epimerase_[EC:5.1.3.41] | K10709 | HC | 2.775 | 0.0156 |
| urtB;_urea_transport_system_permease_protein | K11960 | HC | 2.521 | 0.0156 |
| urtC;_urea_transport_system_permease_protein | K11961 | HC | 2.522 | 0.0156 |
| gfrF;_fructoselysine-6-phosphate_deglycase | K19510 | HC | 2.281 | 0.0156 |
| ramA;_AraC_family_of_transcriptional_regulator,_multidrug_resistance_transcriptional_activator | K18325 | AxSpA | 2.293 | 0.0157 |
| COQ7;_3-demethoxyubiquinol_3-hydroxylase_[EC:1.14.99.60] | K06134 | HC | 2.413 | 0.0180 |
| soxD;_sarcosine_oxidase,_subunit_delta_[EC:1.5.3.1] | K00304 | HC | 2.310 | 0.0180 |
| pepM;_phosphoenolpyruvate_phosphomutase_[EC:5.4.2.9] | K01841 | HC | 2.085 | 0.0180 |
| flgI;_flagellar_P-ring_protein_FlgI | K02394 | HC | 2.391 | 0.0180 |
| gamP;_D-glucosamine_PTS_system_EIICBA_component_[EC:2.7.1.-] | K02765 | HC | 3.272 | 0.0180 |
| urtE;_urea_transport_system_ATP-binding_protein | K11963 | HC | 2.513 | 0.0180 |
| tehB;_tellurite_methyltransferase_[EC:2.1.1.265] | K16868 | AxSpA | 3.768 | 0.0180 |
| K17213;_inositol_transport_system_substrate-binding_protein | K17213 | HC | 2.898 | 0.0180 |
| CHAF1A;_chromatin_assembly_factor_1_subunit_A | K10750 | HC | 2.347 | 0.0182 |
| cpaC,_rcpA;_pilus_assembly_protein_CpaC | K02280 | HC | 2.368 | 0.0192 |
| ipdC;_indolepyruvate_decarboxylase_[EC:4.1.1.74] | K04103 | AxSpA | 2.165 | 0.0199 |
| E2.1.3.1-12S;_methylmalonyl-CoA_carboxyltransferase_12S_subunit_[EC:2.1.3.1] | K17489 | HC | 2.086 | 0.0202 |
| E2.6.1.11,_argD;_acetylornithine_aminotransferase_[EC:2.6.1.11] | K00818 | HC | 3.099 | 0.0206 |
| E4.1.1.18,_ldcC,_cadA;_lysine_decarboxylase_[EC:4.1.1.18] | K01582 | AxSpA | 3.260 | 0.0206 |
| pcaC;_4-carboxymuconolactone_decarboxylase_[EC:4.1.1.44] | K01607 | HC | 3.627 | 0.0206 |
| algL;_poly(beta-D-mannuronate)_lyase_[EC:4.2.2.3] | K01729 | HC | 2.156 | 0.0206 |
| nifH;_nitrogenase_iron_protein_NifH | K02588 | HC | 2.794 | 0.0206 |
| ureJ;_urease_accessory_protein | K03192 | HC | 2.172 | 0.0206 |
| hutF;_formimidoylglutamate_deiminase_[EC:3.5.3.13] | K05603 | HC | 2.096 | 0.0206 |
| xylH;_D-xylose_transport_system_permease_protein | K10544 | AxSpA | 2.920 | 0.0206 |
| dgcB;_diguanylate_cyclase_[EC:2.7.7.65] | K13590 | HC | 2.213 | 0.0206 |
| psuK;_pseudouridine_kinase_[EC:2.7.1.83] | K16328 | HC | 2.988 | 0.0206 |
| croR;_3-hydroxybutyryl-CoA_dehydratase_[EC:4.2.1.55] | K17865 | HC | 2.990 | 0.0206 |
| PTH2;_peptidyl-tRNA_hydrolase,_PTH2_family_[EC:3.1.1.29] | K04794 | HC | 2.922 | 0.0221 |
|  | K00183 | HC | 2.281 | 0.0221 |
| lmrS;_MFS_transporter,_DHA2_family,_multidrug_resistance_protein | K18934 | AxSpA | 2.027 | 0.0233 |
| K09701;_uncharacterized_protein | K09701 | HC | 2.147 | 0.0236 |
| mtnK;_5-methylthioribose_kinase_[EC:2.7.1.100] | K00899 | HC | 2.179 | 0.0236 |
| pct;_propionate_CoA-transferase_[EC:2.8.3.1] | K01026 | HC | 2.923 | 0.0236 |
| PCBD,_phhB;_4a-hydroxytetrahydrobiopterin_dehydratase_[EC:4.2.1.96] | K01724 | HC | 2.466 | 0.0236 |
| fliK;_flagellar_hook-length_control_protein_FliK | K02414 | HC | 3.074 | 0.0236 |
| kdgT;_2-keto-3-deoxygluconate_permease | K02526 | HC | 2.332 | 0.0236 |
| pvdE;_putative_pyoverdin_transport_system_ATP-binding/permease_protein | K06160 | HC | 2.115 | 0.0236 |
| K07317;_adenine-specific_DNA-methyltransferase_[EC:2.1.1.72] | K07317 | HC | 2.395 | 0.0236 |
| paiB;_transcriptional_regulator | K07734 | HC | 2.373 | 0.0236 |
| ABC-2.LPSE.P;_lipopolysaccharide_transport_system_permease_protein | K09690 | HC | 3.355 | 0.0236 |
| K09919;_uncharacterized_protein | K09919 | HC | 2.515 | 0.0236 |
| K09964;_uncharacterized_protein | K09964 | HC | 2.171 | 0.0236 |
| K09986;_uncharacterized_protein | K09986 | HC | 2.159 | 0.0236 |
| urtD;_urea_transport_system_ATP-binding_protein | K11962 | HC | 2.515 | 0.0236 |
| linN;_cholesterol_transport_system_auxiliary_component | K18480 | HC | 2.188 | 0.0236 |
| spuC;_putrescine---pyruvate_transaminase_[EC:2.6.1.113] | K12256 | HC | 2.104 | 0.0252 |
| K18335;_2-keto-3-deoxy-L-fuconate_dehydrogenase_[EC:1.1.1.-] | K18335 | HC | 2.119 | 0.0252 |
| ttuD;_hydroxypyruvate_reductase_[EC:1.1.1.81] | K00050 | HC | 2.995 | 0.0270 |
| allD;_ureidoglycolate_dehydrogenase_(NAD+)_[EC:1.1.1.350] | K00073 | AxSpA | 2.962 | 0.0270 |
| glcD;_glycolate_oxidase_[EC:1.1.3.15] | K00104 | HC | 3.182 | 0.0270 |
| E4.1.1.32,_pckA,_PCK;_phosphoenolpyruvate_carboxykinase_(GTP)_[EC:4.1.1.32] | K01596 | HC | 2.968 | 0.0270 |
| AACS,_acsA;_acetoacetyl-CoA_synthetase_[EC:6.2.1.16] | K01907 | HC | 2.119 | 0.0270 |
| K02477;_two-component_system,_LytTR_family,_response_regulator | K02477 | HC | 3.180 | 0.0270 |
| cyaB;_adenylate_cyclase,_class_2_[EC:4.6.1.1] | K05873 | AxSpA | 3.145 | 0.0270 |
| trbL;_type_IV_secretion_system_protein_TrbL | K07344 | HC | 2.174 | 0.0270 |
| K09928;_uncharacterized_protein | K09928 | HC | 2.112 | 0.0270 |
| aotM;_arginine/ornithine_transport_system_permease_protein | K10023 | HC | 2.115 | 0.0270 |
| aotQ;_arginine/ornithine_transport_system_permease_protein | K10024 | HC | 2.115 | 0.0270 |
| czcB,_cusB,_cnrB;_membrane_fusion_protein,_heavy_metal_efflux_system | K15727 | HC | 2.712 | 0.0270 |
| psuG;_pseudouridylate_synthase_[EC:4.2.1.70] | K16329 | HC | 2.978 | 0.0270 |
| catB;_chloramphenicol_O-acetyltransferase_type_B_[EC:2.3.1.28] | K00638 | HC | 2.233 | 0.0307 |
| K09124;_uncharacterized_protein | K09124 | HC | 2.481 | 0.0307 |
| pobR;_AraC_family_transcriptional_regulator,_transcriptional_activator_of_pobA | K18954 | HC | 2.411 | 0.0307 |
| gltB;_glutamate_synthase_(NADPH)_large_chain_[EC:1.4.1.13] | K00265 | HC | 3.142 | 0.0308 |
| dexB;_glucan_1,6-alpha-glucosidase_[EC:3.2.1.70] | K01215 | AxSpA | 3.275 | 0.0308 |
| paaF,_echA;_enoyl-CoA_hydratase_[EC:4.2.1.17] | K01692 | HC | 2.613 | 0.0308 |
| livK;_branched-chain_amino_acid_transport_system_substrate-binding_protein | K01999 | HC | 3.624 | 0.0308 |
| flgA;_flagellar_basal_body_P-ring_formation_protein_FlgA | K02386 | HC | 2.337 | 0.0308 |
| fliJ;_flagellar_protein_FliJ | K02413 | HC | 3.210 | 0.0308 |
| fliL;_flagellar_protein_FliL | K02415 | HC | 2.529 | 0.0308 |
| pabBC;_para-aminobenzoate_synthetase_/_4-amino-4-deoxychorismate_lyase_[EC:2.6.1.85_4.1.3.38] | K03342 | AxSpA | 3.581 | 0.0308 |
| cheY;_two-component_system,_chemotaxis_family,_chemotaxis_protein_CheY | K03413 | HC | 3.512 | 0.0308 |
| tarJ;_ribitol-5-phosphate_2-dehydrogenase_(NADP+)_[EC:1.1.1.405] | K05352 | AxSpA | 3.227 | 0.0308 |
| K07020;_uncharacterized_protein | K07020 | HC | 2.103 | 0.0308 |
| K07028;_uncharacterized_protein | K07028 | HC | 2.090 | 0.0308 |
| AARSD1,_ALAX;_misacylated_tRNA(Ala)_deacylase_[EC:3.1.1.-] | K07050 | HC | 2.089 | 0.0308 |
| vsr;_DNA_mismatch_endonuclease,_patch_repair_protein_[EC:3.1.-.-] | K07458 | HC | 3.167 | 0.0308 |
| uhpT;_MFS_transporter,_OPA_family,_hexose_phosphate_transport_protein_UhpT | K07784 | AxSpA | 2.267 | 0.0308 |
| CRLS;_cardiolipin_synthase_(CMP-forming)_[EC:2.7.8.41] | K08744 | HC | 3.116 | 0.0308 |
| K09948;_uncharacterized_protein | K09948 | HC | 2.090 | 0.0308 |
| aotJ;_arginine/ornithine_transport_system_substrate-binding_protein | K10022 | HC | 2.102 | 0.0308 |
| int;_integrase | K14059 | HC | 2.271 | 0.0308 |
| glpV;_glycerol_transport_system_substrate-binding_protein | K17321 | HC | 2.085 | 0.0308 |
| K17322,_glpP;_glycerol_transport_system_permease_protein | K17322 | HC | 2.085 | 0.0308 |
| glpQ;_glycerol_transport_system_permease_protein | K17323 | HC | 2.085 | 0.0308 |
| glpS;_glycerol_transport_system_ATP-binding_protein | K17324 | HC | 2.076 | 0.0308 |
| K17325,_glpT;_glycerol_transport_system_ATP-binding_protein | K17325 | HC | 2.076 | 0.0308 |
| gfrC;_fructoselysine/glucoselysine_PTS_system_EIIC_component | K19508 | HC | 2.738 | 0.0308 |
| phnS;_2-aminoethylphosphonate_transport_system_substrate-binding_protein | K11081 | AxSpA | 2.272 | 0.0342 |
| phnV;_2-aminoethylphosphonate_transport_system_permease_protein | K11082 | AxSpA | 2.219 | 0.0342 |
| phnU;_2-aminoethylphosphonate_transport_system_permease_protein | K11083 | AxSpA | 2.272 | 0.0342 |
| phnT;_2-aminoethylphosphonate_transport_system_ATP-binding_protein | K11084 | AxSpA | 2.272 | 0.0342 |
| PGD,_gnd,_gntZ;_6-phosphogluconate_dehydrogenase_[EC:1.1.1.44_1.1.1.343] | K00033 | AxSpA | 3.758 | 0.0350 |
| DAK,_TKFC;_triose/dihydroxyacetone_kinase_/_FAD-AMP_lyase_(cyclizing)_[EC:2.7.1.28_2.7.1.29_4.6.1.15] | K00863 | AxSpA | 3.183 | 0.0350 |
| ppc;_phosphoenolpyruvate_carboxylase_[EC:4.1.1.31] | K01595 | AxSpA | 3.752 | 0.0350 |
| ltaE;_threonine_aldolase_[EC:4.1.2.48] | K01620 | HC | 3.592 | 0.0350 |
| livF;_branched-chain_amino_acid_transport_system_ATP-binding_protein | K01996 | HC | 3.599 | 0.0350 |
| ATPVC,_ntpC,_atpC;_V/A-type_H+/Na+-transporting_ATPase_subunit_C | K02119 | HC | 3.454 | 0.0350 |
| ATPVF,_ntpF,_atpF;_V/A-type_H+/Na+-transporting_ATPase_subunit_F | K02122 | HC | 3.596 | 0.0350 |
| fliH;_flagellar_assembly_protein_FliH | K02411 | HC | 3.008 | 0.0350 |
| mcp;_methyl-accepting_chemotaxis_protein | K03406 | HC | 4.187 | 0.0350 |
| flhB2;_flagellar_biosynthesis_protein | K04061 | HC | 3.238 | 0.0350 |
| phaZ;_poly(3-hydroxybutyrate)_depolymerase_[EC:3.1.1.75] | K05973 | HC | 2.368 | 0.0350 |
| cutF,_nlpE;_copper_homeostasis_protein_(lipoprotein) | K06079 | HC | 2.416 | 0.0350 |
| flaG;_flagellar_protein_FlaG | K06603 | HC | 2.905 | 0.0350 |
| tadB;_tight_adherence_protein_B | K12510 | HC | 3.394 | 0.0350 |
| lhpA;_4-hydroxyproline_epimerase_[EC:5.1.1.8] | K12658 | HC | 2.082 | 0.0350 |
| uxuR;_GntR_family_transcriptional_regulator,_uxu_operon_transcriptional_repressor | K13637 | AxSpA | 2.951 | 0.0350 |
| K13652;_AraC_family_transcriptional_regulator | K13652 | HC | 2.651 | 0.0350 |
| liuC;_methylglutaconyl-CoA_hydratase_[EC:4.2.1.18] | K13766 | HC | 2.460 | 0.0350 |
| gfrA;_fructoselysine/glucoselysine_PTS_system_EIIA_component_[EC:2.7.1.-] | K19506 | HC | 2.661 | 0.0350 |
| cueR;_MerR_family_transcriptional_regulator,_copper_efflux_regulator | K19591 | HC | 2.067 | 0.0350 |
| mngR,_farR;_GntR_family_transcriptional_regulator,_mannosyl-D-glycerate_transport/metabolism_system_repressor | K11922 | HC | 2.138 | 0.0371 |
| K07161;_uncharacterized_protein | K07161 | HC | 2.083 | 0.0372 |
| csxA;_exo-1,4-beta-D-glucosaminidase_[EC:3.2.1.165] | K15855 | HC | 2.074 | 0.0372 |
| hpaX;_MFS_transporter,_ACS_family,_4-hydroxyphenylacetate_permease | K02511 | AxSpA | 2.204 | 0.0393 |
| parD1_3_4;_antitoxin_ParD1/3/4 | K07746 | HC | 2.349 | 0.0396 |
| PCCA,_pccA;_propionyl-CoA_carboxylase_alpha_chain_[EC:6.4.1.3] | K01965 | HC | 2.113 | 0.0396 |
| soxG;_sarcosine_oxidase,_subunit_gamma_[EC:1.5.3.1] | K00305 | HC | 2.083 | 0.0396 |
| aprA;_adenylylsulfate_reductase,_subunit_A_[EC:1.8.99.2] | K00394 | HC | 2.840 | 0.0396 |
| ATPVE,_ntpE,_atpE;_V/A-type_H+/Na+-transporting_ATPase_subunit_E | K02121 | HC | 3.374 | 0.0396 |
| comFB;_competence_protein_ComFB | K02241 | HC | 2.770 | 0.0396 |
| flgD;_flagellar_basal-body_rod_modification_protein_FlgD | K02389 | HC | 3.240 | 0.0396 |
| flgL;_flagellar_hook-associated_protein_3_FlgL | K02397 | HC | 3.214 | 0.0396 |
| flhF;_flagellar_biosynthesis_protein_FlhF | K02404 | HC | 3.014 | 0.0396 |
| fliO,_fliZ;_flagellar_protein_FliO/FliZ | K02418 | HC | 3.193 | 0.0396 |
| cheB;_two-component_system,_chemotaxis_family,_protein-glutamate_methylesterase/glutaminase_[EC:3.1.1.61_3.5.1.44] | K03412 | HC | 3.176 | 0.0396 |
| cheV;_two-component_system,_chemotaxis_family,_chemotaxis_protein_CheV | K03415 | HC | 3.200 | 0.0396 |
| bluB;_5,6-dimethylbenzimidazole_synthase_[EC:1.13.11.79] | K04719 | HC | 2.099 | 0.0396 |
| hutC;_GntR_family_transcriptional_regulator,_histidine_utilization_repressor | K05836 | HC | 2.114 | 0.0396 |
| MuB;_ATP-dependent_target_DNA_activator | K07132 | HC | 2.765 | 0.0396 |
| uhpA;_two-component_system,_NarL_family,_uhpT_operon_response_regulator_UhpA | K07686 | AxSpA | 2.263 | 0.0396 |
| K09153;_small_membrane_protein | K09153 | HC | 3.130 | 0.0396 |
| trpGD;_anthranilate_synthase/phosphoribosyltransferase_[EC:4.1.3.27_2.4.2.18] | K13497 | HC | 2.568 | 0.0396 |
| RTCB,_rtcB;_tRNA-splicing_ligase_RtcB_(3'-phosphate/5'-hydroxy_nucleic_acid_ligase)_[EC:6.5.1.8] | K14415 | HC | 2.628 | 0.0396 |
| qrtT;_energy-coupling_factor_transport_system_substrate-specific_component | K16923 | HC | 3.265 | 0.0396 |
| gfrD;_fructoselysine/glucoselysine_PTS_system_EIID_component | K19509 | HC | 2.666 | 0.0396 |
| ANK;_ankyrin | K10380 | AxSpA | 2.441 | 0.0399 |
| K16306;_fructose-bisphosphate_aldolase_/_2-amino-3,7-dideoxy-D-threo-hept-6-ulosonate_synthase_[EC:4.1.2.13_2.2.1.10] | K16306 | AxSpA | 2.713 | 0.0408 |
| meh;_3-methylfumaryl-CoA_hydratase_[EC:4.2.1.153] | K09709 | HC | 2.054 | 0.0420 |
| andAc;_anthranilate_1,2-dioxygenase_large_subunit_[EC:1.14.12.1] | K16319 | AxSpA | 2.380 | 0.0420 |
| dasC;_N,N'-diacetylchitobiose_transport_system_permease_protein | K17331 | AxSpA | 2.216 | 0.0421 |
| rfbF,_rhlC;_rhamnosyltransferase_[EC:2.4.1.-] | K12990 | HC | 2.188 | 0.0448 |
| cysNC;_bifunctional_enzyme_CysN/CysC_[EC:2.7.7.4_2.7.1.25] | K00955 | HC | 2.030 | 0.0449 |
| accA;_acetyl-CoA_carboxylase_carboxyl_transferase_subunit_alpha_[EC:6.4.1.2_2.1.3.15] | K01962 | AxSpA | 3.523 | 0.0449 |
| flgE;_flagellar_hook_protein_FlgE | K02390 | HC | 3.405 | 0.0449 |
| flhB;_flagellar_biosynthesis_protein_FlhB | K02401 | HC | 3.329 | 0.0449 |
| fliC,_hag;_flagellin | K02406 | HC | 3.619 | 0.0449 |
| fliF;_flagellar_M-ring_protein_FliF | K02409 | HC | 3.231 | 0.0449 |
| fliG;_flagellar_motor_switch_protein_FliG | K02410 | HC | 3.339 | 0.0449 |
| amt,_AMT,_MEP;_ammonium_transporter,_Amt_family | K03320 | HC | 3.738 | 0.0449 |
| TC.BCT;_betaine/carnitine_transporter,_BCCT_family | K03451 | HC | 2.772 | 0.0449 |
| thyX,_thy1;_thymidylate_synthase_(FAD)_[EC:2.1.1.148] | K03465 | HC | 3.170 | 0.0449 |
| cspA;_cold_shock_protein | K03704 | AxSpA | 3.544 | 0.0449 |
| flhG,_fleN;_flagellar_biosynthesis_protein_FlhG | K04562 | HC | 3.045 | 0.0449 |
| gntR;_LacI_family_transcriptional_regulator,_gluconate_utilization_system_Gnt-I_transcriptional_repressor | K06145 | AS | 2.951 | 0.0449 |
| K06915;_uncharacterized_protein | K06915 | AxSpA | 3.741 | 0.0449 |
| ABC-2.LPSE.A;_lipopolysaccharide_transport_system_ATP-binding_protein | K09691 | HC | 3.322 | 0.0449 |
| ppnP;_purine/pyrimidine-nucleoside_phosphorylase_[EC:2.4.2.1_2.4.2.2] | K09913 | HC | 2.392 | 0.0449 |
| allP;_allantoin_permease | K10975 | AxSpA | 2.306 | 0.0449 |
| cbiGH-cobJ;_cobalt-precorrin_5A_hydrolase_/_precorrin-3B_C17-methyltransferase_[EC:3.7.1.12_2.1.1.131] | K13541 | HC | 2.290 | 0.0449 |
| ynaI,_mscMJ;_MscS_family_membrane_protein | K16052 | HC | 2.874 | 0.0449 |
| K17214;_inositol_transport_system_permease_protein | K17214 | HC | 2.737 | 0.0449 |
| gfrB;_fructoselysine/glucoselysine_PTS_system_EIIB_component_[EC:2.7.1.-] | K19507 | HC | 2.679 | 0.0449 |
| dauA;_D-arginine_dehydrogenase_[EC:1.4.99.6] | K19746 | HC | 2.195 | 0.0449 |
| alsA;_D-allose_transport_system_ATP-binding_protein_[EC:7.5.2.8] | K10551 | HC | 2.139 | 0.0473 |
| clfB;_clumping_factor_B | K14192 | AxSpA | 2.340 | 0.0484 |
| drp35;_lactonase_[EC:3.1.1.-] | K02352 | AxSpA | 2.645 | 0.0489 |
| pht5;_4,5-dihydroxyphthalate_decarboxylase_[EC:4.1.1.55] | K04102 | AxSpA | 2.418 | 0.0499 |
| JEN;_MFS_transporter,_SHS_family,_lactate_transporter | K08178 | AxSpA | 2.265 | 0.0501 |
| vraR;_two-component_system,_NarL_family,_vancomycin_resistance_associated_response_regulator_VraR | K07694 | HC | 2.021 | 0.0504 |
| G6PD,_zwf;_glucose-6-phosphate_1-dehydrogenase_[EC:1.1.1.49_1.1.1.363] | K00036 | AxSpA | 3.732 | 0.0506 |
| E1.1.1.67,_mtlK;_mannitol_2-dehydrogenase_[EC:1.1.1.67] | K00045 | HC | 2.070 | 0.0506 |
| E3.1.3.41;_4-nitrophenyl_phosphatase_[EC:3.1.3.41] | K01101 | AxSpA | 3.292 | 0.0506 |
| GBA,_srfJ;_glucosylceramidase_[EC:3.2.1.45] | K01201 | HC | 2.819 | 0.0506 |
| pepN;_aminopeptidase_N_[EC:3.4.11.2] | K01256 | AxSpA | 3.699 | 0.0506 |
| thrH;_phosphoserine_/_homoserine_phosphotransferase_[EC:3.1.3.3_2.7.1.39] | K02203 | HC | 3.073 | 0.0506 |
| cobC,_phpB;_alpha-ribazole_phosphatase_[EC:3.1.3.73] | K02226 | HC | 3.756 | 0.0506 |
| cobQ,_cbiP;_adenosylcobyric_acid_synthase_[EC:6.3.5.10] | K02232 | HC | 3.601 | 0.0506 |
| coxA,_ctaD;_cytochrome_c_oxidase_subunit_I_[EC:7.1.1.9] | K02274 | HC | 2.649 | 0.0506 |
| coxB,_ctaC;_cytochrome_c_oxidase_subunit_II_[EC:7.1.1.9] | K02275 | HC | 2.649 | 0.0506 |
| flgK;_flagellar_hook-associated_protein_1 | K02396 | HC | 3.435 | 0.0506 |
| flgM;_negative_regulator_of_flagellin_synthesis_FlgM | K02398 | HC | 3.233 | 0.0506 |
| fliD;_flagellar_hook-associated_protein_2 | K02407 | HC | 3.339 | 0.0506 |
| ptsG;_glucose_PTS_system_EIICB_or_EIICBA_component_[EC:2.7.1.199] | K02779 | AxSpA | 3.496 | 0.0506 |
| TC.CITMHS;_citrate-Mg2+:H+_or_citrate-Ca2+:H+_symporter,_CitMHS_family | K03300 | HC | 2.525 | 0.0506 |
| ACR3,_arsB;_arsenite_transporter | K03325 | HC | 2.815 | 0.0506 |
| coaX;_type_III_pantothenate_kinase_[EC:2.7.1.33] | K03525 | HC | 3.650 | 0.0506 |
| katE,_CAT,_catB,_srpA;_catalase_[EC:1.11.1.6] | K03781 | AxSpA | 3.631 | 0.0506 |
| lysK;_lysyl-tRNA_synthetase,_class_I_[EC:6.1.1.6] | K04566 | HC | 2.156 | 0.0506 |
| cobD;_threonine-phosphate_decarboxylase_[EC:4.1.1.81] | K04720 | HC | 3.676 | 0.0506 |
| UMF1;_MFS_transporter,_UMF1_family | K06902 | HC | 3.215 | 0.0506 |
| aldA;_lactaldehyde_dehydrogenase_/_glycolaldehyde_dehydrogenase_[EC:1.2.1.22_1.2.1.21] | K07248 | AxSpA | 3.467 | 0.0506 |
| uhpB;_two-component_system,_NarL_family,_sensor_histidine_kinase_UhpB_[EC:2.7.13.3] | K07675 | AxSpA | 2.269 | 0.0506 |
| sstT;_serine/threonine_transporter | K07862 | AxSpA | 3.736 | 0.0506 |
| K08961;_chondroitin-sulfate-ABC_endolyase/exolyase_[EC:4.2.2.20_4.2.2.21] | K08961 | HC | 2.600 | 0.0506 |
| mutM,_fpg;_formamidopyrimidine-DNA_glycosylase_[EC:3.2.2.23_4.2.99.18] | K10563 | AxSpA | 3.720 | 0.0506 |
| nfsA;_nitroreductase_[EC:1.-.-.-] | K10678 | HC | 2.828 | 0.0506 |
| K11312;_cupin_2_domain-containing_protein | K11312 | HC | 2.472 | 0.0506 |
| regB,_regS,_actS;_two-component_system,_sensor_histidine_kinase_RegB_[EC:2.7.13.3] | K15011 | HC | 2.052 | 0.0506 |
| regA,_regR,_actR;_two-component_system,_response_regulator_RegA | K15012 | HC | 2.052 | 0.0506 |
| tcyM;_L-cystine_transport_system_permease_protein | K16959 | AxSpA | 3.532 | 0.0506 |
| tcyN;_L-cystine_transport_system_ATP-binding_protein_[EC:7.4.2.1] | K16960 | AxSpA | 3.314 | 0.0506 |
| hndC;_NADP-reducing_hydrogenase_subunit_HndC_[EC:1.12.1.3] | K18331 | HC | 3.406 | 0.0506 |
| hndD;_NADP-reducing_hydrogenase_subunit_HndD_[EC:1.12.1.3] | K18332 | HC | 3.425 | 0.0506 |
| legG,_neuC2;_GDP/UDP-N,N'-diacetylbacillosamine_2-epimerase_(hydrolysing)_[EC:3.2.1.184] | K18429 | HC | 2.944 | 0.0506 |
| legI,_neuB2;_N,N'-diacetyllegionaminate_synthase_[EC:2.5.1.101] | K18430 | HC | 2.976 | 0.0506 |
| patA,_rscA,_lmrC,_satA;_ATP-binding_cassette,_subfamily_B,_multidrug_efflux_pump | K18891 | AxSpA | 3.193 | 0.0506 |
| patB,_rscB,_lmrC,_satB;_ATP-binding_cassette,_subfamily_B,_multidrug_efflux_pump | K18892 | AxSpA | 3.189 | 0.0506 |
| K09700;_uncharacterized_protein | K09700 | HC | 2.070 | 0.0509 |
| badH;_2-hydroxycyclohexanecarboxyl-CoA_dehydrogenase_[EC:1.1.1.-] | K07535 | HC | 2.425 | 0.0529 |
| ndhG;_NAD(P)H-quinone_oxidoreductase_subunit_6_[EC:7.1.1.2] | K05578 | HC | 2.066 | 0.0545 |
| SPS,_sds;_all-trans-nonaprenyl-diphosphate_synthase_[EC:2.5.1.84_2.5.1.85] | K05356 | HC | 2.069 | 0.0545 |
| ndhB;_NAD(P)H-quinone_oxidoreductase_subunit_2_[EC:7.1.1.2] | K05573 | HC | 2.069 | 0.0545 |
| nblS;_two-component_system,_OmpR_family,_sensor_histidine_kinase_NblS_[EC:2.7.13.3] | K07769 | HC | 2.073 | 0.0545 |
| mgdA;_1,2-diacylglycerol_3-beta-glucosyltransferase_[EC:2.4.1.336] | K19003 | HC | 2.073 | 0.0545 |
| crtISO,_crtH;_prolycopene_isomerase_[EC:5.2.1.13] | K09835 | HC | 2.611 | 0.0564 |
| mdcF;_malonate_transporter_and_related_proteins | K13936 | AxSpA | 2.335 | 0.0566 |
| scrY;_sucrose_porin | K16077 | AxSpA | 2.318 | 0.0566 |
| oqxR;_Rrf2_family_transcriptional_regulator,_repressor_of_oqxAB | K19587 | AxSpA | 2.335 | 0.0566 |
| feaR;_AraC_family_transcriptional_regulator,_positive_regulator_of_tynA_and_feaB | K14063 | AxSpA | 2.306 | 0.0568 |
| matB;_malonyl-CoA/methylmalonyl-CoA_synthetase_[EC:6.2.1.-] | K18661 | HC | 2.059 | 0.0570 |
| paaH,_hbd,_fadB,_mmgB;_3-hydroxybutyryl-CoA_dehydrogenase_[EC:1.1.1.157] | K00074 | HC | 3.277 | 0.0570 |
| IVD,_ivd;_isovaleryl-CoA_dehydrogenase_[EC:1.3.8.4] | K00253 | HC | 2.079 | 0.0570 |
| K00375;_GntR_family_transcriptional_regulator_/_MocR_family_aminotransferase | K00375 | HC | 3.547 | 0.0570 |
| aprB;_adenylylsulfate_reductase,_subunit_B_[EC:1.8.99.2] | K00395 | HC | 2.897 | 0.0570 |
| DLAT,_aceF,_pdhC;_pyruvate_dehydrogenase_E2_component_(dihydrolipoamide_acetyltransferase)_[EC:2.3.1.12] | K00627 | AxSpA | 3.763 | 0.0570 |
| dapD;_2,3,4,5-tetrahydropyridine-2,6-dicarboxylate_N-succinyltransferase_[EC:2.3.1.117] | K00674 | AxSpA | 3.714 | 0.0570 |
| UGP2,_galU,_galF;_UTP--glucose-1-phosphate_uridylyltransferase_[EC:2.7.7.9] | K00963 | AxSpA | 3.707 | 0.0570 |
| E3.2.1.89;_arabinogalactan_endo-1,4-beta-galactosidase_[EC:3.2.1.89] | K01224 | HC | 3.211 | 0.0570 |
| PGAM,_gpmA;_2,3-bisphosphoglycerate-dependent_phosphoglycerate_mutase_[EC:5.4.2.11] | K01834 | AxSpA | 3.706 | 0.0570 |
| livG;_branched-chain_amino_acid_transport_system_ATP-binding_protein | K01995 | HC | 3.605 | 0.0570 |
| livM;_branched-chain_amino_acid_transport_system_permease_protein | K01998 | HC | 3.622 | 0.0570 |
| lamB;_maltoporin | K02024 | AxSpA | 3.008 | 0.0570 |
| ABC.SP.A;_putative_spermidine/putrescine_transport_system_ATP-binding_protein | K02052 | HC | 2.692 | 0.0570 |
| cobB-cbiA;_cobyrinic_acid_a,c-diamide_synthase_[EC:6.3.5.9_6.3.5.11] | K02224 | HC | 3.517 | 0.0570 |
| cobP,_cobU;_adenosylcobinamide_kinase_/_adenosylcobinamide-phosphate_guanylyltransferase_[EC:2.7.1.156_2.7.7.62] | K02231 | HC | 3.697 | 0.0570 |
| E2.7.8.26,_cobS,_cobV;_adenosylcobinamide-GDP_ribazoletransferase_[EC:2.7.8.26] | K02233 | HC | 3.592 | 0.0570 |
| cobW;_cobalamin_biosynthesis_protein_CobW | K02234 | HC | 2.290 | 0.0570 |
| cpaA,_tadV;_prepilin_peptidase_CpaA_[EC:3.4.23.43] | K02278 | HC | 3.030 | 0.0570 |
| flgB;_flagellar_basal-body_rod_protein_FlgB | K02387 | HC | 3.313 | 0.0570 |
| fliE;_flagellar_hook-basal_body_complex_protein_FliE | K02408 | HC | 3.333 | 0.0570 |
| fliR;_flagellar_biosynthesis_protein_FliR | K02421 | HC | 3.327 | 0.0570 |
| flp,_pilA;_pilus_assembly_protein_Flp/PilA | K02651 | HC | 2.634 | 0.0570 |
| cheA;_two-component_system,_chemotaxis_family,_sensor_kinase_CheA_[EC:2.7.13.3] | K03407 | HC | 3.305 | 0.0570 |
| licR;_lichenan_operon_transcriptional_antiterminator | K03491 | HC | 3.074 | 0.0570 |
| hfq;_host_factor-I_protein | K03666 | AxSpA | 3.515 | 0.0570 |
| dinG;_ATP-dependent_DNA_helicase_DinG_[EC:3.6.4.12] | K03722 | AxSpA | 3.663 | 0.0570 |
| arcD,_lysl,_lysP;_arginine:ornithine_antiporter_/_lysine_permease | K03758 | AxSpA | 3.066 | 0.0570 |
| ABCC-BAC;_ATP-binding_cassette,_subfamily_C,_bacterial | K06148 | AxSpA | 3.754 | 0.0570 |
| hr;_hemerythrin | K07216 | HC | 3.337 | 0.0570 |
| spsF;_spore_coat_polysaccharide_biosynthesis_protein_SpsF | K07257 | HC | 2.152 | 0.0570 |
| agrC,_blpH,_fsrC;_two-component_system,_LytTR_family,_sensor_histidine_kinase_AgrC_[EC:2.7.13.3] | K07706 | AxSpA | 3.617 | 0.0570 |
| peb1A,_glnH;_aspartate/glutamate/glutamine_transport_system_substrate-binding_protein | K10039 | AxSpA | 3.235 | 0.0570 |
| E2.7.13.3;_histidine_kinase_[EC:2.7.13.3] | K10819 | AxSpA | 3.289 | 0.0570 |
| slo;_thiol-activated_cytolysin | K11031 | AxSpA | 3.473 | 0.0570 |
| imuA;_protein_ImuA | K14160 | HC | 2.337 | 0.0570 |
| alaA;_alanine-synthesizing_transaminase_[EC:2.6.1.66_2.6.1.2] | K14260 | AxSpA | 3.687 | 0.0570 |
| mgp;_4-O-beta-D-mannosyl-D-glucose_phosphorylase_[EC:2.4.1.281] | K16212 | HC | 3.183 | 0.0570 |
| cbe,_mbe;_cellobiose_epimerase_[EC:5.1.3.11] | K16213 | HC | 3.072 | 0.0570 |
| hndA;_NADP-reducing_hydrogenase_subunit_HndA_[EC:1.12.1.3] | K18330 | HC | 3.418 | 0.0570 |
| algI;_alginate_O-acetyltransferase_complex_protein_AlgI | K19294 | HC | 3.668 | 0.0570 |
| chrR,_NQR;_chromate_reductase,_NAD(P)H_dehydrogenase_(quinone) | K19784 | AxSpA | 3.555 | 0.0570 |
| QPCT;_glutaminyl-peptide_cyclotransferase_[EC:2.3.2.5] | K00683 | AxSpA | 2.478 | 0.0601 |
| K16906;_fluoroquinolone_transport_system_permease_protein | K16906 | AxSpA | 2.178 | 0.0601 |
| thuG,_sugB;_trehalose/maltose_transport_system_permease_protein | K10238 | HC | 2.189 | 0.0602 |
| SHPK;_sedoheptulokinase_[EC:2.7.1.14] | K11214 | HC | 2.143 | 0.0604 |
| K07129;_uncharacterized_protein | K07129 | AxSpA | 2.144 | 0.0607 |
| andAd;_anthranilate_1,2-dioxygenase_small_subunit_[EC:1.14.12.1] | K16320 | AxSpA | 2.364 | 0.0611 |
| aroDE,_DHQ-SDH;_3-dehydroquinate_dehydratase_/_shikimate_dehydrogenase_[EC:4.2.1.10_1.1.1.25] | K13832 | HC | 2.174 | 0.0612 |
| ptcA;_putrescine_carbamoyltransferase_[EC:2.1.3.6] | K13252 | HC | 2.218 | 0.0638 |
| ERCC3,_XPB;_DNA_excision_repair_protein_ERCC-3_[EC:3.6.4.12] | K10843 | HC | 2.127 | 0.0640 |
| gldA;_glycerol_dehydrogenase_[EC:1.1.1.6] | K00005 | HC | 3.185 | 0.0641 |
| ALDH;_aldehyde_dehydrogenase_(NAD+)_[EC:1.2.1.3] | K00128 | HC | 3.250 | 0.0641 |
| GCDH,_gcdH;_glutaryl-CoA_dehydrogenase_[EC:1.3.8.6] | K00252 | HC | 2.377 | 0.0641 |
| gltD;_glutamate_synthase_(NADPH)_small_chain_[EC:1.4.1.13] | K00266 | HC | 3.989 | 0.0641 |
| K00666;_fatty-acyl-CoA_synthase_[EC:6.2.1.-] | K00666 | HC | 3.065 | 0.0641 |
| bioA;_adenosylmethionine---8-amino-7-oxononanoate_aminotransferase_[EC:2.6.1.62] | K00833 | AxSpA | 3.564 | 0.0641 |
| E2.8.3.5A,_scoA;_3-oxoacid_CoA-transferase_subunit_A_[EC:2.8.3.5] | K01028 | HC | 2.412 | 0.0641 |
| spoT;_GTP_diphosphokinase_/_guanosine-3',5'-bis(diphosphate)_3'-diphosphatase_[EC:2.7.6.5_3.1.7.2] | K01139 | AxSpA | 3.646 | 0.0641 |
| gmuG;_mannan_endo-1,4-beta-mannosidase_[EC:3.2.1.78] | K01218 | HC | 3.678 | 0.0641 |
| DNPEP;_aspartyl_aminopeptidase_[EC:3.4.11.21] | K01267 | HC | 3.468 | 0.0641 |
| hipO;_hippurate_hydrolase_[EC:3.5.1.32] | K01451 | HC | 3.065 | 0.0641 |
| E4.1.3.3,_nanA,_NPL;_N-acetylneuraminate_lyase_[EC:4.1.3.3] | K01639 | AxSpA | 3.416 | 0.0641 |
| xylA;_xylose_isomerase_[EC:5.3.1.5] | K01805 | HC | 2.903 | 0.0641 |
| proX;_glycine_betaine/proline_transport_system_substrate-binding_protein | K02002 | AxSpA | 3.171 | 0.0641 |
| bioH;_pimeloyl-[acyl-carrier_protein]_methyl_ester_esterase_[EC:3.1.1.85] | K02170 | AxSpA | 3.435 | 0.0641 |
| cbiB,_cobD;_adenosylcobinamide-phosphate_synthase_[EC:6.3.1.10] | K02227 | HC | 3.611 | 0.0641 |
| COX11,_ctaG;_cytochrome_c_oxidase_assembly_protein_subunit_11 | K02258 | HC | 2.077 | 0.0641 |
| COX15,_ctaA;_cytochrome_c_oxidase_assembly_protein_subunit_15 | K02259 | HC | 2.638 | 0.0641 |
| flgC;_flagellar_basal-body_rod_protein_FlgC | K02388 | HC | 3.327 | 0.0641 |
| flhA;_flagellar_biosynthesis_protein_FlhA | K02400 | HC | 3.316 | 0.0641 |
| fliA,_whiG;_RNA_polymerase_sigma_factor_FliA | K02405 | HC | 3.314 | 0.0641 |
| fliI;_flagellum-specific_ATP_synthase_[EC:7.4.2.8] | K02412 | HC | 3.327 | 0.0641 |
| fliM;_flagellar_motor_switch_protein_FliM | K02416 | HC | 3.320 | 0.0641 |
| fliP;_flagellar_biosynthesis_protein_FliP | K02419 | HC | 3.325 | 0.0641 |
| fliQ;_flagellar_biosynthesis_protein_FliQ | K02420 | HC | 3.326 | 0.0641 |
| fliS;_flagellar_secretion_chaperone_FliS | K02422 | HC | 3.444 | 0.0641 |
| GLPF;_glycerol_uptake_facilitator_protein | K02440 | AxSpA | 3.703 | 0.0641 |
| parC;_topoisomerase_IV_subunit_A_[EC:5.6.2.2] | K02621 | AxSpA | 3.787 | 0.0641 |
| parE;_topoisomerase_IV_subunit_B_[EC:5.6.2.2] | K02622 | AxSpA | 3.790 | 0.0641 |
| ulaE,_sgaU,_sgbU;_L-ribulose-5-phosphate_3-epimerase_[EC:5.1.3.22] | K03079 | HC | 2.404 | 0.0641 |
| phnW;_2-aminoethylphosphonate-pyruvate_transaminase_[EC:2.6.1.37] | K03430 | HC | 2.633 | 0.0641 |
| tlyC;_putative_hemolysin | K03699 | HC | 3.542 | 0.0641 |
| arsC;_arsenate_reductase_(thioredoxin)_[EC:1.20.4.4] | K03741 | HC | 3.154 | 0.0641 |
| E1.2.7.8;_indolepyruvate_ferredoxin_oxidoreductase_[EC:1.2.7.8] | K04090 | HC | 2.034 | 0.0641 |
| E3.1.3.15B;_histidinol-phosphatase_(PHP_family)_[EC:3.1.3.15] | K04486 | HC | 3.682 | 0.0641 |
| sohB;_serine_protease_SohB_[EC:3.4.21.-] | K04774 | AxSpA | 3.672 | 0.0641 |
| slyA;_MarR_family_transcriptional_regulator,_transcriptional_regulator_for_hemolysin | K06075 | HC | 2.670 | 0.0641 |
| phnA;_protein_PhnA | K06193 | AxSpA | 3.808 | 0.0641 |
| jag;_spoIIIJ-associated_protein | K06346 | HC | 3.323 | 0.0641 |
| K06973;_uncharacterized_protein | K06973 | HC | 3.553 | 0.0641 |
| FLOT;_flotillin | K07192 | HC | 3.109 | 0.0641 |
| K07577;_putative_mRNA_3-end_processing_factor | K07577 | HC | 2.013 | 0.0641 |
| zmpB;_zinc_metalloprotease_ZmpB_[EC:3.4.24.-] | K08643 | AxSpA | 3.465 | 0.0641 |
| K08884;_serine/threonine_protein_kinase,_bacterial_[EC:2.7.11.1] | K08884 | HC | 3.765 | 0.0641 |
| malK,_mtlK,_thuK;_multiple_sugar_transport_system_ATP-binding_protein_[EC:3.6.3.-] | K10111 | AxSpA | 3.009 | 0.0641 |
| eamB;_cysteine/O-acetylserine_efflux_protein | K11249 | HC | 2.243 | 0.0641 |
| comA;_ATP-binding_cassette,_subfamily_C,_bacterial,_competence_factor_transporting_protein_[EC:3.4.22.-] | K12292 | AxSpA | 3.171 | 0.0641 |
| PM20D1;_carboxypeptidase_PM20D1_[EC:3.4.17.-] | K13049 | HC | 3.109 | 0.0641 |
| cpoA;_1,2-diacylglycerol-3-alpha-glucose_alpha-1,2-galactosyltransferase_[EC:2.4.1.-] | K13678 | AxSpA | 3.161 | 0.0641 |
| ylbA,_UGHY;_(S)-ureidoglycine_aminohydrolase_[EC:3.5.3.26] | K14977 | AxSpA | 2.952 | 0.0641 |
| czcD,_zitB;_cobalt-zinc-cadmium_efflux_system_protein | K16264 | AxSpA | 3.625 | 0.0641 |
| tcyK;_L-cystine_transport_system_substrate-binding_protein | K16957 | AxSpA | 3.323 | 0.0641 |
| K18333;_L-fucose_dehydrogenase | K18333 | HC | 2.149 | 0.0641 |
| impE;_type_VI_secretion_system_protein_ImpE | K11898 | AxSpA | 2.341 | 0.0673 |
| amnD;_2-aminomuconate_deaminase_[EC:3.5.99.5] | K15067 | AxSpA | 2.451 | 0.0686 |
| tauD;_taurine_dioxygenase_[EC:1.14.11.17] | K03119 | HC | 2.169 | 0.0718 |
| phbB;_acetoacetyl-CoA_reductase_[EC:1.1.1.36] | K00023 | HC | 2.267 | 0.0718 |
| panE,_apbA;_2-dehydropantoate_2-reductase_[EC:1.1.1.169] | K00077 | HC | 3.471 | 0.0718 |
| trmA;_tRNA_(uracil-5-)-methyltransferase_[EC:2.1.1.35] | K00557 | AxSpA | 3.629 | 0.0718 |
| cheR;_chemotaxis_protein_methyltransferase_CheR_[EC:2.1.1.80] | K00575 | HC | 3.314 | 0.0718 |
| serB,_PSPH;_phosphoserine_phosphatase_[EC:3.1.3.3] | K01079 | AxSpA | 3.745 | 0.0718 |
| E3.1.3.25,_IMPA,_suhB;_myo-inositol-1(or_4)-monophosphatase_[EC:3.1.3.25] | K01092 | AxSpA | 3.738 | 0.0718 |
| ENGASE;_mannosyl-glycoprotein_endo-beta-N-acetylglucosaminidase_[EC:3.2.1.96] | K01227 | AxSpA | 3.257 | 0.0718 |
| pepQ;_Xaa-Pro_dipeptidase_[EC:3.4.13.9] | K01271 | AxSpA | 3.469 | 0.0718 |
| cpg;_glutamate_carboxypeptidase_[EC:3.4.17.11] | K01295 | AxSpA | 2.693 | 0.0718 |
| allA;_ureidoglycolate_lyase_[EC:4.3.2.3] | K01483 | HC | 2.457 | 0.0718 |
| purK;_5-(carboxyamino)imidazole_ribonucleotide_synthase_[EC:6.3.4.18] | K01589 | AxSpA | 3.750 | 0.0718 |
| E4.2.1.2AA,_fumA;_fumarate_hydratase_subunit_alpha_[EC:4.2.1.2] | K01677 | HC | 3.528 | 0.0718 |
| E4.2.1.2AB,_fumB;_fumarate_hydratase_subunit_beta_[EC:4.2.1.2] | K01678 | HC | 3.533 | 0.0718 |
| praC,_xylH;_4-oxalocrotonate_tautomerase_[EC:5.3.2.6] | K01821 | AxSpA | 3.643 | 0.0718 |
| ATPVI,_ntpI,_atpI;_V/A-type_H+/Na+-transporting_ATPase_subunit_I | K02123 | HC | 3.692 | 0.0718 |
| ATPVK,_ntpK,_atpK;_V/A-type_H+/Na+-transporting_ATPase_subunit_K | K02124 | HC | 3.695 | 0.0718 |
| coxC,_ctaE;_cytochrome_c_oxidase_subunit_III_[EC:7.1.1.9] | K02276 | HC | 2.617 | 0.0718 |
| cpaF,_tadA;_pilus_assembly_protein_CpaF_[EC:7.4.2.8] | K02283 | HC | 3.547 | 0.0718 |
| motA;_chemotaxis_protein_MotA | K02556 | HC | 3.463 | 0.0718 |
| ubiF;_3-demethoxyubiquinol_3-hydroxylase_[EC:1.14.99.60] | K03184 | AxSpA | 3.013 | 0.0718 |
| cheW;_purine-binding_chemotaxis_protein_CheW | K03408 | HC | 3.339 | 0.0718 |
| cheD;_chemotaxis_protein_CheD_[EC:3.5.1.44] | K03411 | HC | 3.280 | 0.0718 |
| umuD;_DNA_polymerase_V_[EC:3.4.21.-] | K03503 | HC | 2.848 | 0.0718 |
| hrcA;_heat-inducible_transcriptional_repressor | K03705 | HC | 3.364 | 0.0718 |
| glmU;_bifunctional_UDP-N-acetylglucosamine_pyrophosphorylase_/_glucosamine-1-phosphate_N-acetyltransferase_[EC:2.7.7.23_2.3.1.157] | K04042 | AxSpA | 3.639 | 0.0718 |
| truC;_tRNA_pseudouridine65_synthase_[EC:5.4.99.26] | K06175 | AxSpA | 3.677 | 0.0718 |
| spoVG;_stage_V_sporulation_protein_G | K06412 | HC | 3.527 | 0.0718 |
| CHAC,_chaC;_glutathione-specific_gamma-glutamylcyclotransferase_[EC:4.3.2.7] | K07232 | HC | 2.284 | 0.0718 |
| tctC;_putative_tricarboxylic_transport_membrane_protein | K07795 | HC | 2.154 | 0.0718 |
| K09766;_uncharacterized_protein | K09766 | HC | 2.954 | 0.0718 |
| K09769;_uncharacterized_protein | K09769 | HC | 3.010 | 0.0718 |
| perR;_Fur_family_transcriptional_regulator,_peroxide_stress_response_regulator | K09825 | HC | 3.591 | 0.0718 |
| K09989;_uncharacterized_protein | K09989 | HC | 2.422 | 0.0718 |
| tcyB,_yecS;_L-cystine_transport_system_permease_protein | K10009 | AxSpA | 3.575 | 0.0718 |
| peb1B,_glnP,_glnM;_aspartate/glutamate/glutamine_transport_system_permease_protein | K10040 | AxSpA | 3.528 | 0.0718 |
| peb1C,_glnQ;_aspartate/glutamate/glutamine_transport_system_ATP-binding_protein_[EC:7.4.2.1] | K10041 | AxSpA | 3.227 | 0.0718 |
| tesA;_acyl-CoA_thioesterase_I_[EC:3.1.2.-_3.1.2.2_3.1.1.2_3.1.1.5] | K10804 | HC | 2.433 | 0.0718 |
| fryB;_fructose-like_PTS_system_EIIB_component_[EC:2.7.1.-] | K11202 | HC | 2.614 | 0.0718 |
| sugE;_quaternary_ammonium_compound-resistance_protein_SugE | K11741 | HC | 2.576 | 0.0718 |
| tolC;_outer_membrane_protein | K12340 | HC | 3.307 | 0.0718 |
| gpmI;_2,3-bisphosphoglycerate-independent_phosphoglycerate_mutase_[EC:5.4.2.12] | K15633 | HC | 3.616 | 0.0718 |
| K16149;_1,4-alpha-glucan_branching_enzyme_[EC:2.4.1.18] | K16149 | HC | 2.530 | 0.0718 |
| tcyL;_L-cystine_transport_system_permease_protein | K16958 | AxSpA | 3.321 | 0.0718 |
| K17215;_inositol_transport_system_ATP-binding_protein | K17215 | HC | 2.981 | 0.0718 |
| pgpC;_phosphatidylglycerophosphatase_C_[EC:3.1.3.27] | K18697 | AxSpA | 2.137 | 0.0718 |
| ubiI;_2-polyprenylphenol_6-hydroxylase_[EC:1.14.13.240] | K18800 | AxSpA | 3.004 | 0.0718 |
| dhbC;_glycerol_dehydratase_medium_subunit_[EC:4.2.1.30] | K06121 | AxSpA | 2.577 | 0.0732 |
| atzD;_cyanuric_acid_amidohydrolase_[EC:3.5.2.15] | K03383 | HC | 2.176 | 0.0740 |
| AOC3,_AOC2,_tynA;_primary-amine_oxidase_[EC:1.4.3.21] | K00276 | AxSpA | 2.105 | 0.0741 |
| napE;_periplasmic_nitrate_reductase_NapE | K02571 | AxSpA | 2.877 | 0.0755 |
| atzB;_hydroxydechloroatrazine_ethylaminohydrolase_[EC:3.5.4.43] | K03382 | HC | 2.130 | 0.0774 |
| badA;_benzoate-CoA_ligase_[EC:6.2.1.25] | K04110 | AxSpA | 2.335 | 0.0801 |
| boxA;_benzoyl-CoA_2,3-epoxidase_subunit_A_[EC:1.14.13.208] | K15511 | AxSpA | 2.381 | 0.0801 |
| boxB;_benzoyl-CoA_2,3-epoxidase_subunit_B_[EC:1.14.13.208] | K15512 | AxSpA | 2.325 | 0.0801 |
| boxC;_benzoyl-CoA-dihydrodiol_lyase_[EC:4.1.2.44] | K15513 | AxSpA | 2.325 | 0.0801 |
| nicB;_nicotinate_dehydrogenase_subunit_B_[EC:1.17.2.1] | K18030 | AxSpA | 2.373 | 0.0801 |
| uxuB;_fructuronate_reductase_[EC:1.1.1.57] | K00040 | HC | 3.083 | 0.0803 |
| soxA;_sarcosine_oxidase,_subunit_alpha_[EC:1.5.3.1] | K00302 | HC | 2.114 | 0.0803 |
| tmk,_DTYMK;_dTMP_kinase_[EC:2.7.4.9] | K00943 | AxSpA | 3.591 | 0.0803 |
| fnr;_CRP/FNR_family_transcriptional_regulator,_anaerobic_regulatory_protein | K01420 | AxSpA | 3.478 | 0.0803 |
| add,_ADA;_adenosine_deaminase_[EC:3.5.4.4] | K01488 | HC | 3.055 | 0.0803 |
| gshA;_glutamate--cysteine_ligase_[EC:6.3.2.2] | K01919 | AxSpA | 3.615 | 0.0803 |
| E6.3.5.1,_NADSYN1,_QNS1,_nadE;_NAD+_synthase_(glutamine-hydrolysing)_[EC:6.3.5.1] | K01950 | HC | 3.628 | 0.0803 |
| E6.4.1.4A;_3-methylcrotonyl-CoA_carboxylase_alpha_subunit_[EC:6.4.1.4] | K01968 | HC | 2.089 | 0.0803 |
| afuC,_fbpC;_iron(III)_transport_system_ATP-binding_protein_[EC:7.2.2.7] | K02010 | AxSpA | 3.691 | 0.0803 |
| ATPVA,_ntpA,_atpA;_V/A-type_H+/Na+-transporting_ATPase_subunit_A_[EC:7.1.2.2_7.2.2.1] | K02117 | HC | 3.655 | 0.0803 |
| ATPVB,_ntpB,_atpB;_V/A-type_H+/Na+-transporting_ATPase_subunit_B | K02118 | HC | 3.693 | 0.0803 |
| ATPVD,_ntpD,_atpD;_V/A-type_H+/Na+-transporting_ATPase_subunit_D | K02120 | HC | 3.693 | 0.0803 |
| bioC;_malonyl-CoA_O-methyltransferase_[EC:2.1.1.197] | K02169 | AxSpA | 3.576 | 0.0803 |
| flgG;_flagellar_basal-body_rod_protein_FlgG | K02392 | HC | 3.615 | 0.0803 |
| glpT;_MFS_transporter,_OPA_family,_glycerol-3-phosphate_transporter | K02445 | AxSpA | 3.618 | 0.0803 |
| mpl;_UDP-N-acetylmuramate:_L-alanyl-gamma-D-glutamyl-meso-diaminopimelate_ligase_[EC:6.3.2.45] | K02558 | AxSpA | 3.616 | 0.0803 |
| fruAb;_fructose_PTS_system_EIIB_component_[EC:2.7.1.202] | K02769 | HC | 2.832 | 0.0803 |
| rhaM;_L-rhamnose_mutarotase_[EC:5.1.3.32] | K03534 | HC | 3.212 | 0.0803 |
| rdgC;_recombination_associated_protein_RdgC | K03554 | AxSpA | 3.631 | 0.0803 |
| slyX;_SlyX_protein | K03745 | AxSpA | 3.626 | 0.0803 |
| pqiA;_paraquat-inducible_protein_A | K03808 | AxSpA | 3.621 | 0.0803 |
| pflX;_putative_pyruvate_formate_lyase_activating_enzyme_[EC:1.97.1.4] | K04070 | HC | 3.519 | 0.0803 |
| dusC;_tRNA-dihydrouridine_synthase_C_[EC:1.-.-.-] | K05541 | AxSpA | 3.627 | 0.0803 |
| abnA;_arabinan_endo-1,5-alpha-L-arabinosidase_[EC:3.2.1.99] | K06113 | HC | 3.025 | 0.0803 |
| nlpD;_lipoprotein_NlpD | K06194 | AxSpA | 3.673 | 0.0803 |
| cgeB;_spore_maturation_protein_CgeB | K06320 | HC | 3.118 | 0.0803 |
| K06960;_uncharacterized_protein | K06960 | HC | 3.329 | 0.0803 |
| nasT;_two-component_system,_response_regulator_/_RNA-binding_antiterminator | K07183 | HC | 3.506 | 0.0803 |
| K07270;_glycosyl_transferase,_family_25 | K07270 | AxSpA | 3.984 | 0.0803 |
| iorA;_isoquinoline_1-oxidoreductase_subunit_alpha_[EC:1.3.99.16] | K07302 | HC | 2.126 | 0.0803 |
| envZ;_two-component_system,_OmpR_family,_osmolarity_sensor_histidine_kinase_EnvZ_[EC:2.7.13.3] | K07638 | HC | 2.321 | 0.0803 |
| glnL,_ntrB;_two-component_system,_NtrC_family,_nitrogen_regulation_sensor_histidine_kinase_GlnL_[EC:2.7.13.3] | K07708 | HC | 2.327 | 0.0803 |
| tctB;_putative_tricarboxylic_transport_membrane_protein | K07794 | HC | 2.175 | 0.0803 |
| araJ;_MFS_transporter,_DHA1_family,_arabinose_polymer_utilization_protein | K08156 | HC | 2.588 | 0.0803 |
| caiD;_crotonobetainyl-CoA_hydratase_[EC:4.2.1.149] | K08299 | AxSpA | 2.118 | 0.0803 |
| K09794;_uncharacterized_protein | K09794 | AxSpA | 3.625 | 0.0803 |
| K09806;_uncharacterized_protein | K09806 | AxSpA | 3.532 | 0.0803 |
| K09923;_uncharacterized_protein | K09923 | AxSpA | 3.596 | 0.0803 |
| tcyC,_yecC;_L-cystine_transport_system_ATP-binding_protein_[EC:7.4.2.1] | K10010 | AxSpA | 3.559 | 0.0803 |
| potI;_putrescine_transport_system_permease_protein | K11074 | AxSpA | 3.433 | 0.0803 |
| potH;_putrescine_transport_system_permease_protein | K11075 | AxSpA | 3.433 | 0.0803 |
| yhdR;_aspartate_aminotransferase_[EC:2.6.1.1] | K11358 | HC | 3.345 | 0.0803 |
| arsH;_arsenical_resistance_protein_ArsH | K11811 | HC | 2.078 | 0.0803 |
| yiaY;_alcohol_dehydrogenase_[EC:1.1.1.1] | K13954 | HC | 3.276 | 0.0803 |
| imuB;_protein_ImuB | K14161 | HC | 2.354 | 0.0803 |
| ICP;_inhibitor_of_cysteine_peptidase | K14475 | HC | 2.008 | 0.0803 |
| apgM;_2,3-bisphosphoglycerate-independent_phosphoglycerate_mutase_[EC:5.4.2.12] | K15635 | HC | 3.492 | 0.0803 |
| czcC,_cusC,_cnrC;_outer_membrane_protein,_heavy_metal_efflux_system | K15725 | HC | 2.425 | 0.0803 |
| K15977;_putative_oxidoreductase | K15977 | AxSpA | 3.972 | 0.0803 |
| cydC;_ATP-binding_cassette,_subfamily_C,_bacterial_CydC | K16012 | AxSpA | 3.361 | 0.0803 |
| lrgA;_holin-like_protein | K05338 | AxSpA | 2.175 | 0.0827 |
| abgR;_LysR_family_transcriptional_regulator,_regulator_of_abg_operon | K14057 | AxSpA | 2.329 | 0.0830 |
| ACMSD;_aminocarboxymuconate-semialdehyde_decarboxylase_[EC:4.1.1.45] | K03392 | AxSpA | 2.679 | 0.0869 |
| GLYR;_glyoxylate/succinic_semialdehyde_reductase_[EC:1.1.1.79_1.1.1.-] | K18121 | AxSpA | 2.449 | 0.0870 |
| degS;_two-component_system,_NarL_family,_sensor_histidine_kinase_DegS_[EC:2.7.13.3] | K07777 | HC | 2.879 | 0.0896 |
| LDHD,_dld;_D-lactate_dehydrogenase_(cytochrome)_[EC:1.1.2.4] | K00102 | HC | 2.283 | 0.0896 |
| ACADM,_acd;_acyl-CoA_dehydrogenase_[EC:1.3.8.7] | K00249 | HC | 2.788 | 0.0896 |
| ubiG;_2-polyprenyl-6-hydroxyphenyl_methylase_/_3-demethylubiquinone-9_3-methyltransferase_[EC:2.1.1.222_2.1.1.64] | K00568 | AxSpA | 3.518 | 0.0896 |
| PGLS,_pgl,_devB;_6-phosphogluconolactonase_[EC:3.1.1.31] | K01057 | AxSpA | 3.626 | 0.0896 |
| cysQ,_MET22,_BPNT1;_3'(2'),_5'-bisphosphate_nucleotidase_[EC:3.1.3.7] | K01082 | AxSpA | 3.485 | 0.0896 |
| nagZ;_beta-N-acetylhexosaminidase_[EC:3.2.1.52] | K01207 | AxSpA | 3.504 | 0.0896 |
| glsA,_GLS;_glutaminase_[EC:3.5.1.2] | K01425 | HC | 2.936 | 0.0896 |
| apaH;_bis(5'-nucleosyl)-tetraphosphatase_(symmetrical)_[EC:3.6.1.41] | K01525 | AxSpA | 3.617 | 0.0896 |
| E4.2.1.2B,_fumC,_FH;_fumarate_hydratase,_class_II_[EC:4.2.1.2] | K01679 | AxSpA | 3.654 | 0.0896 |
| edd;_phosphogluconate_dehydratase_[EC:4.2.1.12] | K01690 | AxSpA | 3.416 | 0.0896 |
| glyQ;_glycyl-tRNA_synthetase_alpha_chain_[EC:6.1.1.14] | K01878 | AxSpA | 3.754 | 0.0896 |
| glyS;_glycyl-tRNA_synthetase_beta_chain_[EC:6.1.1.14] | K01879 | AxSpA | 3.754 | 0.0896 |
| paaK;_phenylacetate-CoA_ligase_[EC:6.2.1.30] | K01912 | HC | 3.652 | 0.0896 |
| bioD;_dethiobiotin_synthetase_[EC:6.3.3.3] | K01935 | AxSpA | 3.883 | 0.0896 |
| PCCB,_pccB;_propionyl-CoA_carboxylase_beta_chain_[EC:6.4.1.3_2.1.3.15] | K01966 | HC | 3.004 | 0.0896 |
| livH;_branched-chain_amino_acid_transport_system_permease_protein | K01997 | HC | 3.561 | 0.0896 |
| afuB,_fbpB;_iron(III)_transport_system_permease_protein | K02011 | AxSpA | 3.737 | 0.0896 |
| metI;_D-methionine_transport_system_permease_protein | K02072 | AxSpA | 3.726 | 0.0896 |
| bfd;_bacterioferritin-associated_ferredoxin | K02192 | AxSpA | 3.431 | 0.0896 |
| fliN;_flagellar_motor_switch_protein_FliN | K02417 | HC | 3.352 | 0.0896 |
| fliY,_tcyA;_L-cystine_transport_system_substrate-binding_protein | K02424 | v | 3.580 | 0.0896 |
| lolB;_outer_membrane_lipoprotein_LolB | K02494 | AS | 3.615 | 0.0896 |
| hemX;_uroporphyrin-III_C-methyltransferase_[EC:2.1.1.107] | K02496 | AS | 3.627 | 0.0896 |
| priB;_primosomal_replication_protein_N | K02686 | AS | 3.610 | 0.0896 |
| fruB;_fructose_PTS_system_EIIA_component_[EC:2.7.1.202] | K02768 | HC | 2.952 | 0.0896 |
| waaC,_rfaC;_heptosyltransferase_I_[EC:2.4.-.-] | K02841 | AxSpA | 3.723 | 0.0896 |
| ulaD,_sgaH,_sgbH;_3-dehydro-L-gulonate-6-phosphate_decarboxylase_[EC:4.1.1.85] | K03078 | AxSpA | 3.041 | 0.0896 |
| fruR2,_fruR;_DeoR_family_transcriptional_regulator,_fructose_operon_transcriptional_repressor | K03436 | HC | 3.348 | 0.0896 |
| fis;_Fis_family_transcriptional_regulator,_factor_for_inversion_stimulation_protein | K03557 | AxSpA | 3.614 | 0.0896 |
| metR;_LysR_family_transcriptional_regulator,_regulator_for_metE_and_metH | K03576 | AxSpA | 3.615 | 0.0896 |
| recC;_exodeoxyribonuclease_V_gamma_subunit_[EC:3.1.11.5] | K03583 | AxSpA | 3.615 | 0.0896 |
| ftsL;_cell_division_protein_FtsL | K03586 | AxSpA | 3.616 | 0.0896 |
| ftsA;_cell_division_protein_FtsA | K03590 | AxSpA | 3.811 | 0.0896 |
| sspA,_mglA;_stringent_starvation_protein_A | K03599 | AxSpA | 3.617 | 0.0896 |
| sspB;_stringent_starvation_protein_B | K03600 | AxSpA | 3.617 | 0.0896 |
| rep;_ATP-dependent_DNA_helicase_Rep_[EC:3.6.4.12] | K03656 | AxSpA | 3.616 | 0.0896 |
| ubiJ;_ubiquinone_biosynthesis_protein_UbiJ | K03690 | AxSpA | 3.064 | 0.0896 |
| rimJ;_[ribosomal_protein_S5]-alanine_N-acetyltransferase_[EC:2.3.1.267] | K03790 | HC | 3.541 | 0.0896 |
| ampD;_N-acetyl-anhydromuramoyl-L-alanine_amidase_[EC:3.5.1.28] | K03806 | AxSpA | 3.616 | 0.0896 |
| dsbC;_thiol:disulfide_interchange_protein_DsbC_[EC:5.3.4.1] | K03981 | AxSpA | 3.612 | 0.0896 |
| SOD2;_superoxide_dismutase,_Fe-Mn_family_[EC:1.15.1.1] | K04564 | AxSpA | 3.770 | 0.0896 |
| greB;_transcription_elongation_factor_GreB | K04760 | AxSpA | 3.616 | 0.0896 |
| pbpA;_penicillin-binding_protein_A | K05364 | HC | 3.280 | 0.0896 |
| macB;_macrolide_transport_system_ATP-binding/permease_protein_[EC:3.6.3.-] | K05685 | AxSpA | 3.611 | 0.0896 |
| LYSN;_2-aminoadipate_transaminase_[EC:2.6.1.-] | K05825 | HC | 3.257 | 0.0896 |
| clsC;_cardiolipin_synthase_C_[EC:2.7.8.-] | K06132 | AxSpA | 3.425 | 0.0896 |
| corC;_magnesium_and_cobalt_transporter | K06189 | AxSpA | 3.617 | 0.0896 |
| tlyA;_23S_rRNA_(cytidine1920-2'-O)/16S_rRNA_(cytidine1409-2'-O)-methyltransferase_[EC:2.1.1.226_2.1.1.227] | K06442 | HC | 3.331 | 0.0896 |
| nfdA;_N-substituted_formamide_deformylase_[EC:3.5.1.91] | K07047 | HC | 3.189 | 0.0896 |
| pbpG;_serine-type_D-Ala-D-Ala_endopeptidase_(penicillin-binding_protein_7)_[EC:3.4.21.-] | K07262 | AxSpA | 3.616 | 0.0896 |
| prmB;_ribosomal_protein_L3_glutamine_methyltransferase_[EC:2.1.1.298] | K07320 | AxSpA | 3.617 | 0.0896 |
| algH;_putative_transcriptional_regulator | K07735 | AxSpA | 3.547 | 0.0896 |
| sad;_succinate-semialdehyde_dehydrogenase_[EC:1.2.1.16_1.2.1.24] | K08324 | AxSpA | 3.431 | 0.0896 |
| bisC;_biotin/methionine_sulfoxide_reductase_[EC:1.-.-.-] | K08351 | AxSpA | 2.091 | 0.0896 |
| K09158;_uncharacterized_protein | K09158 | AxSpA | 3.618 | 0.0896 |
| K09762;_uncharacterized_protein | K09762 | HC | 3.276 | 0.0896 |
| K09801;_uncharacterized_protein | K09801 | AxSpA | 3.616 | 0.0896 |
| yjgA;_ribosome-associated_protein | K09889 | AxSpA | 3.617 | 0.0896 |
| hda;_DnaA-homolog_protein | K10763 | AxSpA | 3.616 | 0.0896 |
| yciA;_acyl-CoA_thioesterase_YciA_[EC:3.1.2.-] | K10806 | AxSpA | 3.608 | 0.0896 |
| rhlE;_ATP-dependent_RNA_helicase_RhlE_[EC:3.6.4.13] | K11927 | HC | 3.195 | 0.0896 |
| cysB;_LysR_family_transcriptional_regulator,_cys_regulon_transcriptional_activator | K13634 | AxSpA | 3.617 | 0.0896 |
| adhP;_alcohol_dehydrogenase,_propanol-preferring_[EC:1.1.1.1] | K13953 | AxSpA | 3.557 | 0.0896 |
| ttcA;_tRNA_2-thiocytidine_biosynthesis_protein_TtcA | K14058 | AxSpA | 3.618 | 0.0896 |
| legF,_ptmB;_CMP-N,N'-diacetyllegionaminic_acid_synthase_[EC:2.7.7.82] | K18431 | HC | 3.027 | 0.0896 |
| cptA;_toxin_CptA | K19168 | AxSpA | 3.431 | 0.0896 |
| ribT;_riboflavin_biosynthesis_RibT_protein | K02859 | AxSpA | 2.269 | 0.0897 |
| PHYH;_phytanoyl-CoA_hydroxylase_[EC:1.14.11.18] | K00477 | AxSpA | 2.407 | 0.0931 |
| lrgB;_holin-like_protein_LrgB | K05339 | AxSpA | 2.186 | 0.0931 |
| aftA;_galactan_5-O-arabinofuranosyltransferase_[EC:2.4.2.46] | K13686 | HC | 2.372 | 0.0945 |
| sotB;_MFS_transporter,_DHA1_family,_L-arabinose/isopropyl-beta-D-thiogalactopyranoside_export_protein | K08159 | AxSpA | 3.625 | 0.0945 |
| epr;_minor_extracellular_protease_Epr_[EC:3.4.21.-] | K13277 | HC | 2.831 | 0.0973 |
| pimF;_putative_glycosyltransferase_[EC:2.4.-.-] | K13670 | AxSpA | 2.439 | 0.0973 |
| cpt;_chloramphenicol_3-O_phosphotransferase_[EC:2.7.1.-] | K18554 | AxSpA | 2.446 | 0.0973 |
| blaVEB;_beta-lactamase_class_A_VEB_[EC:3.5.2.6] | K19097 | AxSpA | 2.468 | 0.0973 |
| K09966;_uncharacterized_protein | K09966 | HC | 2.088 | 0.0976 |
| epsJ;_glycosyltransferase_EpsJ_[EC:2.4.-.-] | K19427 | AxSpA | 2.237 | 0.0986 |
| pimC;_alpha-1,6-mannosyltransferase_[EC:2.4.1.-] | K14335 | AxSpA | 2.538 | 0.0986 |
| fadD32;_fatty_acid_CoA_ligase_FadD32 | K12428 | HC | 2.379 | 0.0996 |
| pks13;_polyketide_synthase_13 | K12437 | HC | 2.379 | 0.0996 |
| aftC;_arabinofuranan_3-O-arabinosyltransferase_[EC:2.4.2.47] | K16647 | HC | 2.379 | 0.0996 |
| hdhA;_7-alpha-hydroxysteroid_dehydrogenase_[EC:1.1.1.159] | K00076 | HC | 2.632 | 0.0998 |
| lldD;_L-lactate_dehydrogenase_(cytochrome)_[EC:1.1.2.3] | K00101 | AxSpA | 3.588 | 0.0998 |
| rsmC;_16S_rRNA_(guanine1207-N2)-methyltransferase_[EC:2.1.1.172] | K00564 | AxSpA | 3.545 | 0.0998 |
| hepST;_heptaprenyl_diphosphate_synthase_[EC:2.5.1.30] | K00805 | HC | 3.422 | 0.0998 |
| aspC;_aspartate_aminotransferase_[EC:2.6.1.1] | K00813 | AxSpA | 3.630 | 0.0998 |
| ptrA;_protease_III_[EC:3.4.24.55] | K01407 | AxSpA | 3.012 | 0.0998 |
| prlC;_oligopeptidase_A_[EC:3.4.24.70] | K01414 | AxSpA | 3.611 | 0.0998 |
| ACO,_acnA;_aconitate_hydratase_[EC:4.2.1.3] | K01681 | HC | 3.653 | 0.0998 |
| hemC,_HMBS;_hydroxymethylbilane_synthase_[EC:2.5.1.61] | K01749 | AxSpA | 3.490 | 0.0998 |
| ABC.PA.P;_polar_amino_acid_transport_system_permease_protein | K02029 | AxSpA | 3.989 | 0.0998 |
| thiP;_thiamine_transport_system_permease_protein | K02063 | AxSpA | 3.621 | 0.0998 |
| metQ;_D-methionine_transport_system_substrate-binding_protein | K02073 | AxSpA | 3.844 | 0.0998 |
| agaR;_DeoR_family_transcriptional_regulator,_aga_operon_transcriptional_repressor | K02081 | HC | 2.700 | 0.0998 |
| ulaR;_DeoR_family_transcriptional_regulator,_ulaG_and_ulaABCDEF_operon_transcriptional_repressor | K03477 | AxSpA | 3.011 | 0.0998 |
| sbcC,_rad50;_DNA_repair_protein_SbcC/Rad50 | K03546 | HC | 3.386 | 0.0998 |
| recB;_exodeoxyribonuclease_V_beta_subunit_[EC:3.1.11.5] | K03582 | AxSpA | 3.613 | 0.0998 |
| wcaJ;_putative_colanic_acid_biosysnthesis_UDP-glucose_lipid_carrier_transferase | K03606 | HC | 3.119 | 0.0998 |
| dsbB;_protein_dithiol:quinone_oxidoreductase_[EC:1.8.5.9] | K03611 | AxSpA | 3.613 | 0.0998 |
| lolA;_outer_membrane_lipoprotein_carrier_protein | K03634 | AxSpA | 3.604 | 0.0998 |
| dsbA;_protein_dithiol_oxidoreductase_(disulfide-forming)_[EC:1.8.4.15] | K03673 | AxSpA | 3.876 | 0.0998 |
| tenA;_thiaminase_(transcriptional_activator_TenA)_[EC:3.5.99.2] | K03707 | AxSpA | 3.415 | 0.0998 |
| dltD;_D-alanine_transfer_protein | K03740 | AxSpA | 3.250 | 0.0998 |
| sanA;_SanA_protein | K03748 | AxSpA | 3.454 | 0.0998 |
| nrfG;_formate-dependent_nitrite_reductase_complex_subunit_NrfG | K04018 | AxSpA | 3.044 | 0.0998 |
| hscA;_molecular_chaperone_HscA | K04044 | AxSpA | 3.618 | 0.0998 |
| hscB,_HSCB,_HSC20;_molecular_chaperone_HscB | K04082 | AxSpA | 3.618 | 0.0998 |
| oxyR;_LysR_family_transcriptional_regulator,_hydrogen_peroxide-inducible_genes_activator | K04761 | AxSpA | 3.736 | 0.0998 |
| wzzE;_lipopolysaccharide_biosynthesis_protein_WzzE | K05790 | AxSpA | 3.011 | 0.0998 |
| creA;_CreA_protein | K05805 | AxSpA | 3.415 | 0.0998 |
| rluA;_tRNA_pseudouridine32_synthase_/_23S_rRNA_pseudouridine746_synthase_[EC:5.4.99.28_5.4.99.29] | K06177 | AxSpA | 3.944 | 0.0998 |
| bamE,_smpA;_outer_membrane_protein_assembly_factor_BamE | K06186 | AxSpA | 3.616 | 0.0998 |
| cyaY;_CyaY_protein | K06202 | AxSpA | 3.619 | 0.0998 |
| iolR;_DeoR_family_transcriptional_regulator,_myo-inositol_catabolism_operon_repressor | K06608 | HC | 2.347 | 0.0998 |
| PITRM1,_PreP,_CYM1;_presequence_protease_[EC:3.4.24.-] | K06972 | HC | 3.310 | 0.0998 |
| K07030;_uncharacterized_protein | K07030 | HC | 3.311 | 0.0998 |
| mlaB;_phospholipid_transport_system_transporter-binding_protein | K07122 | AxSpA | 3.616 | 0.0998 |
| mipA,_ompV;_MipA_family_protein | K07274 | AxSpA | 2.605 | 0.0998 |
| iorB;_isoquinoline_1-oxidoreductase_subunit_beta_[EC:1.3.99.16] | K07303 | HC | 2.120 | 0.0998 |
| bcr,_tcaB;_MFS_transporter,_DHA1_family,_multidrug_resistance_protein | K07552 | AxSpA | 3.754 | 0.0998 |
| torY;_trimethylamine-N-oxide_reductase_(cytochrome_c),_cytochrome_c-type_subunit_TorY | K07821 | AxSpA | 3.040 | 0.0998 |
| entS;_MFS_transporter,_ENTS_family,_enterobactin_(siderophore)_exporter | K08225 | AxSpA | 2.064 | 0.0998 |
| gatY-kbaY;_tagatose_1,6-diphosphate_aldolase_GatY/KbaY_[EC:4.1.2.40] | K08302 | HC | 2.930 | 0.0998 |
| mltA;_membrane-bound_lytic_murein_transglycosylase_A_[EC:4.2.2.-] | K08304 | AxSpA | 3.610 | 0.0998 |
| K09775;_uncharacterized_protein | K09775 | HC | 3.250 | 0.0998 |
| K09781;_uncharacterized_protein | K09781 | AxSpA | 3.619 | 0.0998 |
| virK;_uncharacterized_protein | K09824 | AxSpA | 3.734 | 0.0998 |
| K09857;_uncharacterized_protein | K09857 | AxSpA | 3.413 | 0.0998 |
| E2.6.1.83;_LL-diaminopimelate_aminotransferase_[EC:2.6.1.83] | K10206 | HC | 3.712 | 0.0998 |
| fwdE,_fmdE;_formylmethanofuran_dehydrogenase_subunit_E_[EC:1.2.7.12] | K11261 | HC | 2.215 | 0.0998 |
| iscA;_iron-sulfur_cluster_assembly_protein | K13628 | AxSpA | 3.631 | 0.0998 |
| gsiA;_glutathione_transport_system_ATP-binding_protein | K13892 | AxSpA | 2.101 | 0.0998 |
| mpaA;_murein_peptide_amidase_A | K14054 | AxSpA | 2.074 | 0.0998 |
| dnaE2;_error-prone_DNA_polymerase_[EC:2.7.7.7] | K14162 | HC | 2.375 | 0.0998 |
| cbrT;_energy-coupling_factor_transport_system_substrate-specific_component | K16927 | HC | 2.745 | 0.0998 |
| alsE;_D-allulose-6-phosphate_3-epimerase_[EC:5.1.3.-] | K17195 | HC | 2.316 | 0.0998 |
| vcaM;_ATP-binding_cassette,_subfamily_B,_multidrug_efflux_pump | K18893 | AxSpA | 3.620 | 0.0998 |
| yccA;_modulator_of_FtsH_protease | K19416 | AxSpA | 3.612 | 0.0998 |
| nicA;_nicotinate_dehydrogenase_subunit_A_[EC:1.17.2.1] | K18029 | AxSpA | 2.269 | 0.0999 |

| **Saliva_AxSpA vs HC_IgA-_a0.1_w0.05_l2_minc.default.res.sig.txt** | | | | |
| --- | --- | --- | --- | --- |
| **Level D (KO) _Description** | **KO** | **Class with highest mean** | **Log LDA score** | **p-value (KW for class)** |
| E3.2.1.58;_glucan_1,3-beta-glucosidase_[EC:3.2.1.58] | K01210 | HC | 2.362 | 0.0012 |
| pydC;_beta-ureidopropionase_/_N-carbamoyl-L-amino-acid_hydrolase_[EC:3.5.1.6_3.5.1.87] | K06016 | HC | 3.481 | 0.0020 |
| hpdB;_4-hydroxyphenylacetate_decarboxylase_large_subunit_[EC:4.1.1.83] | K18427 | HC | 2.279 | 0.0027 |
| ELP3,_KAT9;_elongator_complex_protein_3_[EC:2.3.1.48] | K07739 | HC | 2.334 | 0.0028 |
| LYSN;_2-aminoadipate_transaminase_[EC:2.6.1.-] | K05825 | HC | 3.599 | 0.0029 |
| pksJ;_polyketide_synthase_PksJ | K13611 | HC | 2.429 | 0.0029 |
| nox2;_NADH_oxidase_(H2O-forming)_[EC:1.6.3.4] | K17869 | HC | 2.420 | 0.0033 |
| pcaC;_4-carboxymuconolactone_decarboxylase_[EC:4.1.1.44] | K01607 | HC | 3.685 | 0.0034 |
| K09726;_uncharacterized_protein | K09726 | HC | 2.602 | 0.0043 |
| livG;_branched-chain_amino_acid_transport_system_ATP-binding_protein | K01995 | HC | 3.743 | 0.0047 |
| mocA;_molybdenum_cofactor_cytidylyltransferase_[EC:2.7.7.76] | K07141 | HC | 3.550 | 0.0047 |
| E3.3.1.1,_ahcY;_adenosylhomocysteinase_[EC:3.3.1.1] | K01251 | HC | 3.563 | 0.0055 |
| livF;_branched-chain_amino_acid_transport_system_ATP-binding_protein | K01996 | HC | 3.766 | 0.0055 |
| livM;_branched-chain_amino_acid_transport_system_permease_protein | K01998 | HC | 3.775 | 0.0055 |
| treS;_maltose_alpha-D-glucosyltransferase_/_alpha-amylase_[EC:5.4.99.16_3.2.1.1] | K05343 | HC | 2.872 | 0.0055 |
| hisI;_phosphoribosyl-AMP_cyclohydrolase_[EC:3.5.4.19] | K01496 | HC | 3.452 | 0.0064 |
| hisB;_imidazoleglycerol-phosphate_dehydratase_[EC:4.2.1.19] | K01693 | HC | 3.664 | 0.0064 |
| livH;_branched-chain_amino_acid_transport_system_permease_protein | K01997 | HC | 3.693 | 0.0064 |
| amt,_AMT,_MEP;_ammonium_transporter,_Amt_family | K03320 | HC | 3.677 | 0.0064 |
| xdhC;_xanthine_dehydrogenase_accessory_factor | K07402 | HC | 3.972 | 0.0064 |
| splA;_transcriptional_regulator_of_the_spore_photoproduct_lyase_operon | K06315 | AxSpA | 2.700 | 0.0066 |
| cfa;_cyclopropane-fatty-acyl-phospholipid_synthase_[EC:2.1.1.79] | K00574 | HC | 3.496 | 0.0087 |
| cobL-cbiET;_precorrin-6B_C5,15-methyltransferase_/_cobalt-precorrin-6B_C5,C15-methyltransferase_[EC:2.1.1.132_2.1.1.289_2.1.1.196] | K00595 | HC | 3.289 | 0.0087 |
| dat;_D-alanine_transaminase_[EC:2.6.1.21] | K00824 | HC | 3.437 | 0.0087 |
| E4.2.1.2AB,_fumB;_fumarate_hydratase_subunit_beta_[EC:4.2.1.2] | K01678 | HC | 3.634 | 0.0087 |
| ABC.MN.S;_manganese/iron_transport_system_substrate-binding_protein | K09818 | HC | 2.318 | 0.0090 |
| E4.2.1.2AA,_fumA;_fumarate_hydratase_subunit_alpha_[EC:4.2.1.2] | K01677 | HC | 3.634 | 0.0101 |
| mtnA;_methylthioribose-1-phosphate_isomerase_[EC:5.3.1.23] | K08963 | HC | 3.498 | 0.0101 |
| ispDF;_2-C-methyl-D-erythritol_4-phosphate_cytidylyltransferase_/_2-C-methyl-D-erythritol_2,4-cyclodiphosphate_synthase_[EC:2.7.7.60_4.6.1.12] | K12506 | HC | 3.543 | 0.0101 |
| croR;_3-hydroxybutyryl-CoA_dehydratase_[EC:4.2.1.55] | K17865 | HC | 2.935 | 0.0101 |
| aac6-I,_aacA7;_aminoglycoside_6'-N-acetyltransferase_I_[EC:2.3.1.82] | K18816 | HC | 2.621 | 0.0101 |
| GLYR;_glyoxylate/succinic_semialdehyde_reductase_[EC:1.1.1.79_1.1.1.-] | K18121 | AxSpA | 2.511 | 0.0114 |
| glcD;_glycolate_oxidase_[EC:1.1.3.15] | K00104 | HC | 3.333 | 0.0117 |
| DPEP;_membrane_dipeptidase_[EC:3.4.13.19] | K01273 | HC | 3.515 | 0.0117 |
| livK;_branched-chain_amino_acid_transport_system_substrate-binding_protein | K01999 | HC | 3.823 | 0.0117 |
| hisZ;_ATP_phosphoribosyltransferase_regulatory_subunit | K02502 | HC | 3.671 | 0.0117 |
| flhB2;_flagellar_biosynthesis_protein | K04061 | HC | 3.019 | 0.0117 |
| larC;_pyridinium-3,5-bisthiocarboxylic_acid_mononucleotide_nickel_chelatase_[EC:4.99.1.12] | K09121 | HC | 3.511 | 0.0117 |
| lysK;_lysyl-tRNA_synthetase,_class_I_[EC:6.1.1.6] | K04566 | HC | 2.239 | 0.0135 |
| ntrX;_two-component_system,_NtrC_family,_nitrogen_regulation_response_regulator_NtrX | K13599 | HC | 2.056 | 0.0135 |
| NRT,_narK,_nrtP,_nasA;_MFS_transporter,_NNP_family,_nitrate/nitrite_transporter | K02575 | HC | 3.439 | 0.0136 |
| ugtP;_processive_1,2-diacylglycerol_beta-glucosyltransferase_[EC:2.4.1.315] | K03429 | HC | 3.494 | 0.0136 |
| dfx;_superoxide_reductase_[EC:1.15.1.2] | K05919 | HC | 3.691 | 0.0136 |
| larE;_pyridinium-3,5-biscarboxylic_acid_mononucleotide_sulfurtransferase_[EC:4.4.1.37] | K06864 | HC | 3.464 | 0.0136 |
| larB;_pyridinium-3,5-biscarboxylic_acid_mononucleotide_synthase_[EC:2.5.1.143] | K06898 | HC | 3.463 | 0.0136 |
| K09777;_uncharacterized_protein | K09777 | HC | 3.576 | 0.0136 |
| xylH;_D-xylose_transport_system_permease_protein | K10544 | AxSpA | 3.027 | 0.0136 |
| srlR,_gutR;_DeoR_family_transcriptional_regulator,_glucitol_operon_repressor | K02468 | AxSpA | 3.026 | 0.0144 |
| mtlR;_mannitol_operon_repressor | K02562 | AxSpA | 3.028 | 0.0144 |
| wzyE,_rffT;_enterobacterial_common_antigen_polymerase_[EC:2.4.1.-] | K02853 | AxSpA | 3.028 | 0.0144 |
| wecF,_rffT;_dTDP-N-acetylfucosamine:lipid_II_N-acetylfucosaminyltransferase_[EC:2.4.1.325] | K12582 | AxSpA | 3.028 | 0.0144 |
| rffC,_wecD;_dTDP-4-amino-4,6-dideoxy-D-galactose_acyltransferase_[EC:2.3.1.210] | K16704 | AxSpA | 3.028 | 0.0144 |
| sirB;_sirohydrochlorin_ferrochelatase_[EC:4.99.1.4] | K03794 | AxSpA | 2.778 | 0.0148 |
| comP;_two-component_system,_NarL_family,_sensor_histidine_kinase_ComP_[EC:2.7.13.3] | K07680 | AxSpA | 2.443 | 0.0148 |
| comA;_two-component_system,_NarL_family,_competent_response_regulator_ComA | K07691 | AxSpA | 2.731 | 0.0148 |
| GLUD1_2,_gdhA;_glutamate_dehydrogenase_(NAD(P)+)_[EC:1.4.1.3] | K00261 | HC | 3.418 | 0.0156 |
| hisE;_phosphoribosyl-ATP_pyrophosphohydrolase_[EC:3.6.1.31] | K01523 | HC | 3.439 | 0.0156 |
| rbcL,_cbbL;_ribulose-bisphosphate_carboxylase_large_chain_[EC:4.1.1.39] | K01601 | HC | 3.418 | 0.0156 |
| pnbA;_para-nitrobenzyl_esterase_[EC:3.1.1.-] | K03929 | HC | 2.408 | 0.0156 |
| cdgJ;_c-di-GMP_phosphodiesterase_[EC:3.1.4.52] | K07181 | HC | 2.686 | 0.0156 |
| thuG,_sugB;_trehalose/maltose_transport_system_permease_protein | K10238 | HC | 2.481 | 0.0172 |
| ltnD;_L-threonate_2-dehydrogenase_[EC:1.1.1.411] | K08319 | AxSpA | 3.024 | 0.0176 |
| mcp;_methyl-accepting_chemotaxis_protein | K03406 | HC | 3.942 | 0.0180 |
| tsr;_methyl-accepting_chemotaxis_protein_I,_serine_sensor_receptor | K05874 | AxSpA | 2.176 | 0.0180 |
| K07099;_uncharacterized_protein | K07099 | HC | 3.568 | 0.0180 |
| TC.SMR3;_small_multidrug_resistance_family-3_protein | K09771 | HC | 3.449 | 0.0180 |
| fbaB;_fructose-bisphosphate_aldolase,_class_I_[EC:4.1.2.13] | K11645 | HC | 2.763 | 0.0180 |
| pct;_propionate_CoA-transferase_[EC:2.8.3.1] | K01026 | HC | 2.879 | 0.0192 |
| SASP-A,_sspA;_small_acid-soluble_spore_protein_A_(major_alpha-type_SASP) | K06418 | AxSpA | 2.431 | 0.0204 |
| raxA;_membrane_fusion_protein | K13408 | AxSpA | 3.026 | 0.0206 |
| GLU,_gltS;_glutamate_synthase_(ferredoxin)_[EC:1.4.7.1] | K00284 | HC | 3.543 | 0.0206 |
| pdp;_pyrimidine-nucleoside_phosphorylase_[EC:2.4.2.2] | K00756 | HC | 3.552 | 0.0206 |
| deoB;_phosphopentomutase_[EC:5.4.2.7] | K01839 | HC | 3.553 | 0.0206 |
| flgL;_flagellar_hook-associated_protein_3_FlgL | K02397 | HC | 2.984 | 0.0206 |
| yabN;_tetrapyrrole_methylase_family_protein_/_MazG_family_protein | K02499 | HC | 3.562 | 0.0206 |
| paaI;_acyl-CoA_thioesterase_[EC:3.1.2.-] | K02614 | HC | 3.519 | 0.0206 |
| chlI,_bchI;_magnesium_chelatase_subunit_I_[EC:6.6.1.1] | K03405 | HC | 3.393 | 0.0206 |
| cheD;_chemotaxis_protein_CheD_[EC:3.5.1.44] | K03411 | HC | 3.028 | 0.0206 |
| spoVB;_stage_V_sporulation_protein_B | K06409 | HC | 3.871 | 0.0206 |
| PITRM1,_PreP,_CYM1;_presequence_protease_[EC:3.4.24.-] | K06972 | HC | 3.564 | 0.0206 |
| ABC-2.LPSE.P;_lipopolysaccharide_transport_system_permease_protein | K09690 | HC | 3.005 | 0.0206 |
| K09807;_uncharacterized_protein | K09807 | HC | 3.475 | 0.0206 |
| mtaD;_5-methylthioadenosine/S-adenosylhomocysteine_deaminase_[EC:3.5.4.31_3.5.4.28] | K12960 | HC | 3.538 | 0.0206 |
| uxuR;_GntR_family_transcriptional_regulator,_uxu_operon_transcriptional_repressor | K13637 | AxSpA | 3.051 | 0.0206 |
| fliB;_lysine-N-methylase_[EC:2.1.1.-] | K18475 | HC | 3.595 | 0.0206 |
| mcsB;_protein_arginine_kinase_[EC:2.7.14.1] | K19405 | HC | 3.494 | 0.0206 |
| nudJ;_phosphatase_NudJ_[EC:3.6.1.-] | K12152 | AxSpA | 3.026 | 0.0219 |
| rusA;_crossover_junction_endodeoxyribonuclease_RusA_[EC:3.1.22.4] | K01160 | AxSpA | 3.094 | 0.0235 |
| prlF,_sohA;_antitoxin_PrlF | K19156 | AxSpA | 2.019 | 0.0235 |
| agaR;_DeoR_family_transcriptional_regulator,_aga_operon_transcriptional_repressor | K02081 | HC | 2.645 | 0.0236 |
| flhB;_flagellar_biosynthesis_protein_FlhB | K02401 | HC | 3.075 | 0.0236 |
| fliF;_flagellar_M-ring_protein_FliF | K02409 | HC | 3.003 | 0.0236 |
| spoVD;_stage_V_sporulation_protein_D_(sporulation-specific_penicillin-binding_protein) | K08384 | HC | 3.715 | 0.0236 |
| E2.6.1.83;_LL-diaminopimelate_aminotransferase_[EC:2.6.1.83] | K10206 | HC | 3.725 | 0.0236 |
| murEF;_MurE/MurF_fusion_protein_[EC:6.3.2.13_6.3.2.10] | K15792 | HC | 2.847 | 0.0236 |
| thiT;_thiamine_transporter | K16789 | HC | 3.544 | 0.0236 |
| mcsA;_protein_arginine_kinase_activator | K19411 | HC | 3.490 | 0.0236 |
| citR;_LysR_family_transcriptional_regulator,_repressor_for_citA | K19242 | HC | 2.492 | 0.0261 |
| yhaV;_toxin_YhaV_[EC:3.1.-.-] | K19155 | AxSpA | 2.033 | 0.0269 |
| rfbF,_rhlC;_rhamnosyltransferase_[EC:2.4.1.-] | K12990 | HC | 2.268 | 0.0270 |
| panE,_apbA;_2-dehydropantoate_2-reductase_[EC:1.1.1.169] | K00077 | HC | 3.428 | 0.0270 |
| TST,_MPST,_sseA;_thiosulfate/3-mercaptopyruvate_sulfurtransferase_[EC:2.8.1.1_2.8.1.2] | K01011 | HC | 3.361 | 0.0270 |
| flgE;_flagellar_hook_protein_FlgE | K02390 | HC | 3.097 | 0.0270 |
| flgG;_flagellar_basal-body_rod_protein_FlgG | K02392 | HC | 3.315 | 0.0270 |
| flgJ;_peptidoglycan_hydrolase_FlgJ | K02395 | HC | 2.746 | 0.0270 |
| fliD;_flagellar_hook-associated_protein_2 | K02407 | HC | 3.092 | 0.0270 |
| fliN;_flagellar_motor_switch_protein_FliN | K02417 | HC | 3.106 | 0.0270 |
| cheA;_two-component_system,_chemotaxis_family,_sensor_kinase_CheA_[EC:2.7.13.3] | K03407 | HC | 3.077 | 0.0270 |
| umuC;_DNA_polymerase_V | K03502 | HC | 3.702 | 0.0270 |
| coxS;_aerobic_carbon-monoxide_dehydrogenase_small_subunit_[EC:1.2.5.3] | K03518 | HC | 3.568 | 0.0270 |
| tagA,_tarA;_N-acetylglucosaminyldiphosphoundecaprenol_N-acetyl-beta-D-mannosaminyltransferase_[EC:2.4.1.187] | K05946 | HC | 3.494 | 0.0270 |
| dinD;_DNA-damage-inducible_protein_D | K14623 | HC | 3.370 | 0.0270 |
| addA;_ATP-dependent_helicase/nuclease_subunit_A_[EC:3.1.-.-_3.6.4.12] | K16898 | HC | 3.624 | 0.0270 |
| addB;_ATP-dependent_helicase/nuclease_subunit_B_[EC:3.1.-.-_3.6.4.12] | K16899 | HC | 3.609 | 0.0270 |
| gltC;_LysR_family_transcriptional_regulator,_transcription_activator_of_glutamate_synthase_operon | K09681 | AxSpA | 2.429 | 0.0283 |
| torA;_trimethylamine-N-oxide_reductase_(cytochrome_c)_[EC:1.7.2.3] | K07811 | AxSpA | 3.024 | 0.0285 |
| flgB;_flagellar_basal-body_rod_protein_FlgB | K02387 | HC | 3.043 | 0.0308 |
| flgD;_flagellar_basal-body_rod_modification_protein_FlgD | K02389 | HC | 2.997 | 0.0308 |
| flgK;_flagellar_hook-associated_protein_1 | K02396 | HC | 3.140 | 0.0308 |
| fliG;_flagellar_motor_switch_protein_FliG | K02410 | HC | 3.091 | 0.0308 |
| fliH;_flagellar_assembly_protein_FliH | K02411 | HC | 2.918 | 0.0308 |
| fliS;_flagellar_secretion_chaperone_FliS | K02422 | HC | 3.176 | 0.0308 |
| nifH;_nitrogenase_iron_protein_NifH | K02588 | HC | 2.958 | 0.0308 |
| phoH2;_PhoH-like_ATPase | K07175 | HC | 2.659 | 0.0308 |
| K07571;_S1_RNA_binding_domain_protein | K07571 | HC | 3.517 | 0.0308 |
| queE;_7-carboxy-7-deazaguanine_synthase_[EC:4.3.99.3] | K10026 | HC | 3.456 | 0.0308 |
| E2.1.3.1-5S;_methylmalonyl-CoA_carboxyltransferase_5S_subunit_[EC:2.1.3.1] | K03416 | HC | 2.535 | 0.0317 |
| E2.1.3.1-1.3S;_methylmalonyl-CoA_carboxyltransferase_1.3S_subunit_[EC:2.1.3.1] | K17490 | HC | 2.535 | 0.0317 |
| degU;_two-component_system,_NarL_family,_response_regulator_DegU | K07692 | AxSpA | 2.632 | 0.0323 |
| AKR1A1,_adh;_alcohol_dehydrogenase_(NADP+)_[EC:1.1.1.2] | K00002 | HC | 2.559 | 0.0324 |
| ccr;_crotonyl-CoA_carboxylase/reductase_[EC:1.3.1.85] | K14446 | HC | 2.062 | 0.0334 |
| K09124;_uncharacterized_protein | K09124 | HC | 2.149 | 0.0339 |
| E3.4.11.14;_cytosol_alanyl_aminopeptidase_[EC:3.4.11.14] | K01263 | AxSpA | 2.867 | 0.0343 |
| ubiF;_3-demethoxyubiquinol_3-hydroxylase_[EC:1.14.99.60] | K03184 | AxSpA | 3.021 | 0.0349 |
| ttuD;_hydroxypyruvate_reductase_[EC:1.1.1.81] | K00050 | HC | 2.788 | 0.0350 |
| ACAT,_atoB;_acetyl-CoA_C-acetyltransferase_[EC:2.3.1.9] | K00626 | HC | 3.468 | 0.0350 |
| HK;_hexokinase_[EC:2.7.1.1] | K00844 | HC | 2.809 | 0.0350 |
| speD,_AMD1;_S-adenosylmethionine_decarboxylase_[EC:4.1.1.50] | K01611 | HC | 3.771 | 0.0350 |
| flgC;_flagellar_basal-body_rod_protein_FlgC | K02388 | HC | 3.076 | 0.0350 |
| fliA,_whiG;_RNA_polymerase_sigma_factor_FliA | K02405 | HC | 3.072 | 0.0350 |
| fliC,_hag;_flagellin | K02406 | HC | 3.372 | 0.0350 |
| fliI;_flagellum-specific_ATP_synthase_[EC:7.4.2.8] | K02412 | HC | 3.075 | 0.0350 |
| fliM;_flagellar_motor_switch_protein_FliM | K02416 | HC | 3.074 | 0.0350 |
| fliP;_flagellar_biosynthesis_protein_FliP | K02419 | HC | 3.075 | 0.0350 |
| fliQ;_flagellar_biosynthesis_protein_FliQ | K02420 | HC | 3.075 | 0.0350 |
| fliR;_flagellar_biosynthesis_protein_FliR | K02421 | HC | 3.071 | 0.0350 |
| ctsR;_transcriptional_regulator_of_stress_and_heat_shock_response | K03708 | HC | 3.459 | 0.0350 |
| K07089;_uncharacterized_protein | K07089 | HC | 3.596 | 0.0350 |
| dgoT;_MFS_transporter,_ACS_family,_D-galactonate_transporter | K08194 | HC | 2.443 | 0.0350 |
| E2.7.7.65;_diguanylate_cyclase_[EC:2.7.7.65] | K13069 | AxSpA | 2.209 | 0.0350 |
| oxc;_oxalyl-CoA_decarboxylase_[EC:4.1.1.8] | K01577 | AxSpA | 2.076 | 0.0366 |
| glcP;_MFS_transporter,_FHS_family,_glucose/mannose:H+_symporter | K08174 | AxSpA | 2.773 | 0.0366 |
| dppE;_dipeptide_transport_system_substrate-binding_protein | K16199 | AxSpA | 2.778 | 0.0366 |
| dppB1;_dipeptide_transport_system_permease_protein | K16200 | AxSpA | 2.779 | 0.0366 |
| tuaH;_teichuronic_acid_biosynthesis_glycosyltransferase_TuaH_[EC:2.4.-.-] | K16699 | AxSpA | 2.773 | 0.0366 |
| tuaF;_teichuronic_acid_biosynthesis_protein_TuaF | K16706 | AxSpA | 2.782 | 0.0366 |
| pucL;_urate_oxidase_/_2-oxo-4-hydroxy-4-carboxy-5-ureidoimidazoline_decarboxylase_[EC:1.7.3.3_4.1.1.97] | K16838 | AxSpA | 2.794 | 0.0366 |
| csxA;_exo-1,4-beta-D-glucosaminidase_[EC:3.2.1.165] | K15855 | HC | 2.224 | 0.0390 |
| E2.7.1.76,_dak;_deoxyadenosine_kinase_[EC:2.7.1.76] | K10353 | AxSpA | 2.795 | 0.0395 |
| cooS,_acsA;_anaerobic_carbon-monoxide_dehydrogenase_catalytic_subunit_[EC:1.2.7.4] | K00198 | HC | 3.413 | 0.0396 |
| aprB;_adenylylsulfate_reductase,_subunit_B_[EC:1.8.99.2] | K00395 | HC | 2.519 | 0.0396 |
| purNH;_phosphoribosylglycinamide/phosphoribosylaminoimidazolecarboxamide_formyltransferase_[EC:2.1.2.2_2.1.2.3] | K01492 | HC | 2.664 | 0.0396 |
| wcaJ;_putative_colanic_acid_biosysnthesis_UDP-glucose_lipid_carrier_transferase | K03606 | HC | 2.827 | 0.0396 |
| moaB;_molybdopterin_adenylyltransferase_[EC:2.7.7.75] | K03638 | HC | 3.451 | 0.0396 |
| RP-L7A,_rplGB;_large_subunit_ribosomal_protein_L7A | K07590 | HC | 3.461 | 0.0396 |
| aroG,_aroA;_3-deoxy-7-phosphoheptulonate_synthase_/_chorismate_mutase_[EC:2.5.1.54_5.4.99.5] | K13853 | HC | 2.133 | 0.0396 |
| aarC,_cat1;_succinyl-CoA:acetate_CoA-transferase_[EC:2.8.3.18] | K18118 | HC | 3.375 | 0.0396 |
| menI,_DHNAT;_1,4-dihydroxy-2-naphthoyl-CoA_hydrolase_[EC:3.1.2.28] | K19222 | AxSpA | 2.219 | 0.0396 |
| K11476,_gntR;_GntR_family_transcriptional_regulator,_gluconate_operon_transcriptional_repressor | K11476 | AxSpA | 2.466 | 0.0415 |
| fabL;_enoyl-[acyl-carrier_protein]_reductase_III_[EC:1.3.1.104] | K10780 | AxSpA | 2.620 | 0.0420 |
| pgpC;_phosphatidylglycerophosphatase_C_[EC:3.1.3.27] | K18697 | AxSpA | 2.097 | 0.0422 |
| aph3-III;_aminoglycoside_3'-phosphotransferase_III_[EC:2.7.1.95] | K19299 | HC | 2.055 | 0.0442 |
| hemDX;_uroporphyrinogen_III_methyltransferase_/_synthase_[EC:2.1.1.107_4.2.1.75] | K13543 | HC | 2.517 | 0.0444 |
| dndD;_DNA_sulfur_modification_protein_DndD | K19171 | HC | 2.225 | 0.0447 |
| fliJ;_flagellar_protein_FliJ | K02413 | HC | 2.962 | 0.0449 |
| nifE;_nitrogenase_molybdenum-cofactor_synthesis_protein_NifE | K02587 | HC | 2.583 | 0.0449 |
| cheY;_two-component_system,_chemotaxis_family,_chemotaxis_protein_CheY | K03413 | HC | 3.298 | 0.0449 |
| spoVS;_stage_V_sporulation_protein_S | K06416 | HC | 2.662 | 0.0449 |
| trmK;_tRNA_(adenine22-N1)-methyltransferase_[EC:2.1.1.217] | K06967 | HC | 3.607 | 0.0449 |
| fsr;_MFS_transporter,_FSR_family,_fosmidomycin_resistance_protein | K08223 | HC | 3.767 | 0.0449 |
| ttrC;_tetrathionate_reductase_subunit_C | K08359 | AxSpA | 3.009 | 0.0449 |
| K13652;_AraC_family_transcriptional_regulator | K13652 | HC | 2.313 | 0.0449 |
| dnaE2;_error-prone_DNA_polymerase_[EC:2.7.7.7] | K14162 | HC | 2.612 | 0.0449 |
| dacA;_diadenylate_cyclase_[EC:2.7.7.85] | K18672 | HC | 3.606 | 0.0449 |
| esxA,_esat6;_6_kDa_early_secretory_antigenic_target | K14956 | HC | 2.787 | 0.0470 |
| pimB;_phosphatidyl-myo-inositol_dimannoside_synthase_[EC:2.4.1.346] | K13668 | HC | 2.383 | 0.0476 |
| mcl;_malyl-CoA/(S)-citramalyl-CoA_lyase_[EC:4.1.3.24_4.1.3.25] | K08691 | HC | 2.161 | 0.0484 |
| ipdC;_indolepyruvate_decarboxylase_[EC:4.1.1.74] | K04103 | AxSpA | 2.093 | 0.0493 |
| ptrA;_protease_III_[EC:3.4.24.55] | K01407 | AxSpA | 3.018 | 0.0505 |
| malM;_maltose_operon_periplasmic_protein | K05775 | AxSpA | 3.017 | 0.0505 |
| wzzE;_lipopolysaccharide_biosynthesis_protein_WzzE | K05790 | AxSpA | 3.018 | 0.0505 |
| malF;_maltose/maltodextrin_transport_system_permease_protein | K10109 | AxSpA | 3.018 | 0.0505 |
| malG;_maltose/maltodextrin_transport_system_permease_protein | K10110 | AxSpA | 3.018 | 0.0505 |
| hcaE,_hcaA1;_3-phenylpropionate/trans-cinnamate_dioxygenase_subunit_alpha_[EC:1.14.12.19] | K05708 | AxSpA | 2.044 | 0.0506 |
| hcaF,_hcaA2;_3-phenylpropionate/trans-cinnamate_dioxygenase_subunit_beta_[EC:1.14.12.19] | K05709 | AxSpA | 2.041 | 0.0506 |
| hcaB;_2,3-dihydroxy-2,3-dihydrophenylpropionate_dehydrogenase_[EC:1.3.1.87] | K05711 | AxSpA | 2.044 | 0.0506 |
| spsF;_spore_coat_polysaccharide_biosynthesis_protein_SpsF | K07257 | HC | 2.141 | 0.0506 |
| argC;_N-acetyl-gamma-glutamyl-phosphate_reductase_[EC:1.2.1.38] | K00145 | HC | 3.550 | 0.0506 |
| lldP,_lctP;_L-lactate_permease | K00427 | AxSpA | 2.062 | 0.0506 |
| ACR3,_arsB;_arsenite_transporter | K03325 | HC | 2.782 | 0.0506 |
| gudP;_MFS_transporter,_ACS_family,_glucarate_transporter | K03535 | HC | 2.407 | 0.0506 |
| pflX;_putative_pyruvate_formate_lyase_activating_enzyme_[EC:1.97.1.4] | K04070 | HC | 3.231 | 0.0506 |
| bofA;_inhibitor_of_the_pro-sigma_K_processing_machinery | K06317 | HC | 3.034 | 0.0506 |
| oxlT;_MFS_transporter,_OFA_family,_oxalate/formate_antiporter | K08177 | HC | 3.610 | 0.0506 |
| ABC-2.LPSE.A;_lipopolysaccharide_transport_system_ATP-binding_protein | K09691 | HC | 2.948 | 0.0506 |
| leuE;_leucine_efflux_protein | K11250 | AxSpA | 2.245 | 0.0506 |
| K15383;_MtN3_and_saliva_related_transmembrane_protein | K15383 | HC | 2.496 | 0.0506 |
| mdtO;_multidrug_resistance_protein_MdtO | K15547 | AxSpA | 2.031 | 0.0506 |
| mdtN;_membrane_fusion_protein,_multidrug_efflux_system | K15549 | AxSpA | 2.025 | 0.0506 |
| ecm;_ethylmalonyl-CoA_mutase_[EC:5.4.99.63] | K14447 | HC | 2.046 | 0.0514 |
| E2.1.3.1-12S;_methylmalonyl-CoA_carboxyltransferase_12S_subunit_[EC:2.1.3.1] | K17489 | HC | 2.174 | 0.0529 |
| spo0F;_two-component_system,_response_regulator,_stage_0_sporulation_protein_F | K02490 | AxSpA | 2.722 | 0.0552 |
| malE;_maltose/maltodextrin_transport_system_substrate-binding_protein | K10108 | AxSpA | 3.016 | 0.0569 |
| cybC;_soluble_cytochrome_b562 | K15536 | AxSpA | 3.018 | 0.0569 |
| mhpC;_2-hydroxy-6-oxonona-2,4-dienedioate_hydrolase_[EC:3.7.1.14] | K05714 | AxSpA | 2.020 | 0.0570 |
| hcaR;_LysR_family_transcriptional_regulator,_hca_operon_transcriptional_activator | K05817 | AxSpA | 2.002 | 0.0570 |
| caiE;_carnitine_operon_protein_CaiE | K08279 | AxSpA | 2.038 | 0.0570 |
| steA,_tetA46;_ATP-binding_cassette,_subfamily_B,_tetracycline_resistant_protein | K18216 | HC | 2.439 | 0.0570 |
| steB,_tetB46;_ATP-binding_cassette,_subfamily_B,_tetracycline_resistant_protein | K18217 | HC | 2.439 | 0.0570 |
| iorB;_indolepyruvate_ferredoxin_oxidoreductase,_beta_subunit_[EC:1.2.7.8] | K00180 | HC | 3.140 | 0.0570 |
| aprA;_adenylylsulfate_reductase,_subunit_A_[EC:1.8.99.2] | K00394 | HC | 2.497 | 0.0570 |
| ACO,_acnA;_aconitate_hydratase_[EC:4.2.1.3] | K01681 | HC | 3.415 | 0.0570 |
| PC,_pyc;_pyruvate_carboxylase_[EC:6.4.1.1] | K01958 | HC | 3.455 | 0.0570 |
| nrfG;_formate-dependent_nitrite_reductase_complex_subunit_NrfG | K04018 | AxSpA | 3.040 | 0.0570 |
| feoA;_ferrous_iron_transport_protein_A | K04758 | HC | 3.849 | 0.0570 |
| mhpB;_2,3-dihydroxyphenylpropionate_1,2-dioxygenase_[EC:1.13.11.16] | K05713 | AxSpA | 2.012 | 0.0570 |
| K06962;_uncharacterized_protein | K06962 | HC | 3.440 | 0.0570 |
| entS;_MFS_transporter,_ENTS_family,_enterobactin_(siderophore)_exporter | K08225 | AxSpA | 2.034 | 0.0570 |
| K10120,_msmE;_fructooligosaccharide_transport_system_substrate-binding_protein | K10120 | HC | 2.473 | 0.0570 |
| K10121,_msmF;_fructooligosaccharide_transport_system_permease_protein | K10121 | HC | 2.479 | 0.0570 |
| K10122,_msmG;_fructooligosaccharide_transport_system_permease_protein | K10122 | HC | 2.479 | 0.0570 |
| frlC;_fructoselysine_3-epimerase_[EC:5.1.3.41] | K10709 | HC | 2.319 | 0.0570 |
| K10907;_aminotransferase_[EC:2.6.1.-] | K10907 | HC | 3.424 | 0.0570 |
| yhdR;_aspartate_aminotransferase_[EC:2.6.1.1] | K11358 | HC | 3.120 | 0.0570 |
| imuB;_protein_ImuB | K14161 | HC | 2.595 | 0.0570 |
| sdmt;_sarcosine/dimethylglycine_N-methyltransferase_[EC:2.1.1.157] | K18897 | HC | 2.199 | 0.0612 |
| cadB;_cadaverine:lysine_antiporter | K03757 | AxSpA | 2.006 | 0.0624 |
| ABC.MR;_putative_ABC_transport_system_ATP-binding_protein | K02021 | HC | 2.555 | 0.0638 |
| ulaR;_DeoR_family_transcriptional_regulator,_ulaG_and_ulaABCDEF_operon_transcriptional_repressor | K03477 | AxSpA | 3.014 | 0.0639 |
| mdtH;_MFS_transporter,_DHA1_family,_multidrug_resistance_protein | K08162 | AxSpA | 2.190 | 0.0640 |
| fosB;_metallothiol_transferase_[EC:2.5.1.-] | K11210 | HC | 2.288 | 0.0640 |
| yesW;_rhamnogalacturonan_endolyase_[EC:4.2.2.23] | K18197 | AxSpA | 2.644 | 0.0640 |
| neuA,_nnaC;_N-acylneuraminate_cytidylyltransferase_[EC:2.7.7.43] | K00983 | AxSpA | 2.932 | 0.0641 |
| allC;_allantoate_deiminase_[EC:3.5.3.9] | K02083 | AxSpA | 3.042 | 0.0641 |
| ulaD,_sgaH,_sgbH;_3-dehydro-L-gulonate-6-phosphate_decarboxylase_[EC:4.1.1.85] | K03078 | AxSpA | 3.038 | 0.0641 |
| virB6,_lvhB6;_type_IV_secretion_system_protein_VirB6 | K03201 | AxSpA | 2.262 | 0.0641 |
| TC.AAT;_amino_acid_transporter,_AAT_family | K03293 | HC | 3.376 | 0.0641 |
| phnW;_2-aminoethylphosphonate-pyruvate_transaminase_[EC:2.6.1.37] | K03430 | HC | 2.440 | 0.0641 |
| PTH2;_peptidyl-tRNA_hydrolase,_PTH2_family_[EC:3.1.1.29] | K04794 | HC | 2.551 | 0.0641 |
| E2.4.1.4;_amylosucrase_[EC:2.4.1.4] | K05341 | AxSpA | 2.422 | 0.0641 |
| exoX;_exodeoxyribonuclease_X_[EC:3.1.11.-] | K10857 | AxSpA | 2.011 | 0.0641 |
| ssuB;_sulfonate_transport_system_ATP-binding_protein_[EC:7.6.2.14] | K15555 | AxSpA | 2.942 | 0.0641 |
| cbrT;_energy-coupling_factor_transport_system_substrate-specific_component | K16927 | HC | 2.486 | 0.0641 |
| ihk;_two-component_system,_OmpR_family,_sensor_kinase_Ihk_[EC:2.7.13.3] | K18986 | HC | 2.459 | 0.0641 |
| fldB;_flavodoxin_II | K03840 | AxSpA | 2.189 | 0.0678 |
| nifHD1,_nifI1;_nitrogen_regulatory_protein_PII_1 | K02589 | HC | 2.287 | 0.0716 |
| nifHD2,_nifI2;_nitrogen_regulatory_protein_PII_2 | K02590 | HC | 2.287 | 0.0716 |
| nrfF;_formate-dependent_nitrite_reductase_complex_subunit_NrfF | K04017 | AxSpA | 3.016 | 0.0716 |
| caiD;_crotonobetainyl-CoA_hydratase_[EC:4.2.1.149] | K08299 | AxSpA | 2.080 | 0.0716 |
| agaB;_galactosamine_PTS_system_EIIB_component_[EC:2.7.1.-] | K10984 | AxSpA | 2.033 | 0.0716 |
| agaC;_galactosamine_PTS_system_EIIC_component | K10985 | AxSpA | 2.038 | 0.0716 |
| agaD;_galactosamine_PTS_system_EIID_component | K10986 | AxSpA | 2.038 | 0.0716 |
| mccA;_cystathionine_beta-synthase_(O-acetyl-L-serine)_[EC:2.5.1.134] | K17216 | AxSpA | 2.135 | 0.0717 |
| mccB;_cystathionine_gamma-lyase_/_homocysteine_desulfhydrase_[EC:4.4.1.1_4.4.1.2] | K17217 | AxSpA | 2.135 | 0.0717 |
| hisD;_histidinol_dehydrogenase_[EC:1.1.1.23] | K00013 | HC | 3.572 | 0.0718 |
| SCD,_desC;_stearoyl-CoA_desaturase_(Delta-9_desaturase)_[EC:1.14.19.1] | K00507 | HC | 2.525 | 0.0718 |
| argD;_acetylornithine/N-succinyldiaminopimelate_aminotransferase_[EC:2.6.1.11_2.6.1.17] | K00821 | HC | 3.486 | 0.0718 |
| lyxK;_L-xylulokinase_[EC:2.7.1.53] | K00880 | AxSpA | 3.055 | 0.0718 |
| paaK;_phenylacetate-CoA_ligase_[EC:6.2.1.30] | K01912 | HC | 3.168 | 0.0718 |
| fucR;_DeoR_family_transcriptional_regulator,_L-fucose_operon_activator | K02430 | AxSpA | 3.053 | 0.0718 |
| srlA;_glucitol/sorbitol_PTS_system_EIIC_component | K02783 | AxSpA | 3.358 | 0.0718 |
| bglG1;_transcriptional_antiterminator | K03493 | HC | 2.453 | 0.0718 |
| flhG,_fleN;_flagellar_biosynthesis_protein_FlhG | K04562 | HC | 2.807 | 0.0718 |
| repA;_regulatory_protein_RepA | K07505 | HC | 2.394 | 0.0718 |
| K07576;_metallo-beta-lactamase_family_protein | K07576 | HC | 3.194 | 0.0718 |
| ner,_nlp,_sfsB;_Ner_family_transcriptional_regulator | K07724 | AxSpA | 2.452 | 0.0718 |
| glnP;_glutamine_transport_system_permease_protein | K10037 | AxSpA | 2.045 | 0.0718 |
| fliW;_flagellar_assembly_factor_FliW | K13626 | HC | 2.942 | 0.0718 |
| aroKB;_shikimate_kinase_/_3-dehydroquinate_synthase_[EC:2.7.1.71_4.2.3.4] | K13829 | HC | 2.175 | 0.0718 |
| lldR;_GntR_family_transcriptional_regulator,_L-lactate_dehydrogenase_operon_regulator | K14348 | AxSpA | 2.092 | 0.0718 |
| nac;_LysR_family_transcriptional_regulator,_nitrogen_assimilation_regulatory_protein | K19338 | AxSpA | 2.005 | 0.0718 |
| FDH;_formate_dehydrogenase_[EC:1.17.1.9] | K00122 | HC | 2.111 | 0.0730 |
| meh;_3-methylfumaryl-CoA_hydratase_[EC:4.2.1.153] | K09709 | HC | 2.210 | 0.0757 |
| cobIJ;_precorrin-2_C20-methyltransferase_/_precorrin-3B_C17-methyltransferase_[EC:2.1.1.130_2.1.1.131] | K13540 | HC | 2.582 | 0.0759 |
| hcaC;_3-phenylpropionate/trans-cinnamate_dioxygenase_ferredoxin_component | K05710 | AxSpA | 2.040 | 0.0759 |
| NDUFAF7;_NADH_dehydrogenase_[ubiquinone]_1_alpha_subcomplex_assembly_factor_7 | K18164 | HC | 2.193 | 0.0761 |
| mcd;_(2S)-methylsuccinyl-CoA_dehydrogenase_[EC:1.3.8.12] | K14448 | HC | 2.042 | 0.0774 |
| pagP,_crcA;_lipid_IVA_palmitoyltransferase_[EC:2.3.1.251] | K12973 | AxSpA | 2.029 | 0.0798 |
| epsH;_glycosyltransferase_EpsH_[EC:2.4.-.-] | K19425 | AxSpA | 2.355 | 0.0801 |
| fadK;_acyl-CoA_synthetase_[EC:6.2.1.-] | K12507 | AxSpA | 2.026 | 0.0803 |
| iorA;_indolepyruvate_ferredoxin_oxidoreductase,_alpha_subunit_[EC:1.2.7.8] | K00179 | HC | 3.050 | 0.0803 |
| GALT29A;_beta-1,6-galactosyltransferase_[EC:2.4.1.-] | K00786 | HC | 2.446 | 0.0803 |
| argB;_acetylglutamate_kinase_[EC:2.7.2.8] | K00930 | HC | 3.543 | 0.0803 |
| thrH;_phosphoserine_/_homoserine_phosphotransferase_[EC:3.1.3.3_2.7.1.39] | K02203 | HC | 2.863 | 0.0803 |
| dnaC;_DNA_replication_protein_DnaC | K02315 | HC | 3.693 | 0.0803 |
| hisF;_imidazole_glycerol-phosphate_synthase_subunit_HisF_[EC:4.3.2.10] | K02500 | HC | 3.599 | 0.0803 |
| mhpD;_2-keto-4-pentenoate_hydratase_[EC:4.2.1.80] | K02554 | AxSpA | 2.038 | 0.0803 |
| clpL;_ATP-dependent_Clp_protease_ATP-binding_subunit_ClpL | K04086 | HC | 3.363 | 0.0803 |
| mhpT;_MFS_transporter,_AAHS_family,_3-hydroxyphenylpropionic_acid_transporter | K05819 | AxSpA | 2.034 | 0.0803 |
| tar;_methyl-accepting_chemotaxis_protein_II,_aspartate_sensor_receptor | K05875 | AxSpA | 2.052 | 0.0803 |
| dexA;_dextranase_[EC:3.2.1.11] | K05988 | HC | 2.985 | 0.0803 |
| nfeD;_membrane-bound_serine_protease_(ClpP_class) | K07403 | HC | 3.418 | 0.0803 |
| torY;_trimethylamine-N-oxide_reductase_(cytochrome_c),_cytochrome_c-type_subunit_TorY | K07821 | AxSpA | 3.036 | 0.0803 |
| K09384;_uncharacterized_protein | K09384 | HC | 3.249 | 0.0803 |
| rfbN;_rhamnosyltransferase_[EC:2.4.1.-] | K12992 | HC | 2.741 | 0.0803 |
| pleD;_two-component_system,_cell_cycle_response_regulator_[EC:2.7.7.65] | K02488 | HC | 2.055 | 0.0846 |
| ycnJ;_copper_transport_protein | K14166 | AxSpA | 2.075 | 0.0847 |
| tipF;_cyclic-di-GMP_phosphodiesterase,_flagellum_assembly_factor_TipF | K13593 | HC | 2.060 | 0.0850 |
| mch,_mcd;_2-methylfumaryl-CoA_hydratase_[EC:4.2.1.148] | K14449 | HC | 2.054 | 0.0850 |
| salK;_two-component_system,_NarL_family,_secretion_system_sensor_histidine_kinase_SalK | K14988 | HC | 2.872 | 0.0877 |
| narH,_narY,_nxrB;_nitrate_reductase_/_nitrite_oxidoreductase,_beta_subunit_[EC:1.7.5.1_1.7.99.-] | K00371 | HC | 3.317 | 0.0896 |
| narI,_narV;_nitrate_reductase_gamma_subunit_[EC:1.7.5.1_1.7.99.-] | K00374 | HC | 3.316 | 0.0896 |
| E3.4.16.4;_D-alanyl-D-alanine_carboxypeptidase_[EC:3.4.16.4] | K01286 | HC | 3.168 | 0.0896 |
| ABC.MR.TX;_HlyD_family_secretion_protein | K02022 | HC | 2.137 | 0.0896 |
| hisH;_imidazole_glycerol-phosphate_synthase_subunit_HisH_[EC:4.3.2.10] | K02501 | HC | 3.551 | 0.0896 |
| TC.PST;_polysaccharide_transporter,_PST_family | K03328 | HC | 3.410 | 0.0896 |
| cheC;_chemotaxis_protein_CheC | K03410 | HC | 2.894 | 0.0896 |
| cotH;_spore_coat_protein_H | K06330 | HC | 2.475 | 0.0896 |
| agrB;_accessory_gene_regulator_B | K07813 | HC | 3.000 | 0.0896 |
| hcxA;_hydroxycarboxylate_dehydrogenase_A_[EC:1.1.1.-] | K08317 | AxSpA | 2.526 | 0.0896 |
| rbsC;_ribose_transport_system_permease_protein | K10440 | HC | 3.462 | 0.0896 |
| glcC;_GntR_family_transcriptional_regulator,_glc_operon_transcriptional_activator | K11474 | AxSpA | 2.036 | 0.0896 |
| bgaB,_lacA;_beta-galactosidase_[EC:3.2.1.23] | K12308 | HC | 3.138 | 0.0896 |
| rgpA;_rhamnosyltransferase_[EC:2.4.1.-] | K12996 | HC | 2.490 | 0.0896 |
| irr;_two-component_system,_OmpR_family,_response_regulator_Irr | K18987 | HC | 2.444 | 0.0896 |
| isdG,_isdI;_heme_oxygenase_(staphylobilin-producing)_[EC:1.14.99.48] | K07145 | AxSpA | 2.082 | 0.0934 |
| PM20D1;_carboxypeptidase_PM20D1_[EC:3.4.17.-] | K13049 | HC | 2.671 | 0.0945 |
| budC;_meso-butanediol_dehydrogenase_/_(S,S)-butanediol_dehydrogenase_/_diacetyl_reductase_[EC:1.1.1.-_1.1.1.76_1.1.1.304] | K18009 | AxSpA | 2.331 | 0.0965 |
| hemAT;_heam-based_aerotactic_trancducer | K06595 | AxSpA | 2.567 | 0.0973 |
| K07013;_uncharacterized_protein | K07013 | HC | 2.149 | 0.0977 |
| ligB;_protocatechuate_4,5-dioxygenase,_beta_chain_[EC:1.13.11.8] | K04101 | AxSpA | 2.370 | 0.0997 |
| fucO;_lactaldehyde_reductase_[EC:1.1.1.77] | K00048 | HC | 3.364 | 0.0998 |
| ATE1;_arginyl-tRNA---protein_transferase_[EC:2.3.2.8] | K00685 | HC | 2.596 | 0.0998 |
| nfo;_deoxyribonuclease_IV_[EC:3.1.21.2] | K01151 | HC | 3.508 | 0.0998 |
| flhF;_flagellar_biosynthesis_protein_FlhF | K02404 | HC | 2.757 | 0.0998 |
| sigI;_RNA_polymerase_sigma_factor | K03093 | AxSpA | 2.206 | 0.0998 |
| pat,_bar;_phosphinothricin_acetyltransferase_[EC:2.3.1.183] | K03823 | HC | 3.529 | 0.0998 |
| flaG;_flagellar_protein_FlaG | K06603 | HC | 2.689 | 0.0998 |
| tylC,_oleB,_carA,_srmB;_macrolide_transport_system_ATP-binding/permease_protein | K18230 | HC | 2.149 | 0.0998 |
| fabM;_trans-2-decenoyl-[acyl-carrier_protein]_isomerase_[EC:5.3.3.14] | K18474 | HC | 3.363 | 0.0998 |
